# Perinatal outcomes among pregnant women with HIV initiating antiretroviral therapy preconception and antenatally

**DOI:** 10.1097/QAD.0000000000004104

**Published:** 2025-01-09

**Authors:** Pippa Boering, Claudia Murray, Clara Portwood, Molly Hey, Lucy Thompson, Katharina Beck, Imogen Cowdell, Harriet Sexton, Mary Kumarendran, Zoe Brandon, Shona Kirtley, Joris Hemelaar

**Affiliations:** a Infectious Disease Epidemiology Unit, Nuffield Department of Population Health; b Centre for Statistics in Medicine, Nuffield Department of Orthopaedics, Rheumatology and Musculoskeletal Sciences, University of Oxford, Oxford, UK.

**Keywords:** antiretroviral therapy, HIV, low birthweight, pregnancy, preterm birth, small for gestational age

## Abstract

**Objective:**

Increasingly, pregnant women with HIV (WHIV) initiate antiretroviral therapy (ART) before conception. We assessed the risk of adverse perinatal outcomes among pregnant WHIV initiating ART preconception or antenatally, compared with women without HIV or ART-naive WHIV.

**Design:**

Systematic review and meta-analysis

**Methods:**

We searched PubMed, EMBASE, CINAHL, and Global Health for studies published between 1 January 1980 and 14 July 2023. We assessed the association of preconception/antenatal ART initiation with preterm birth (PTB), very PTB (VPTB), spontaneous PTB (sPTB), low birthweight (LBW), very LBW (VLBW), small for gestational age (SGA), very SGA (VSGA), stillbirth and neonatal death (NND). Data were analysed using random effects meta-analyses. Quality assessments, subgroup and sensitivity analyses were conducted. PROSPERO registration: CRD42021248987.

**Results:**

Thirty-one cohort studies were eligible, including 199 156 women in 19 countries. WHIV with preconception ART were associated with increased risk of PTB [risk ratio (RR) 1.55; 95% confidence interval (CI) 1.27–1.90], VPTB (RR 2.14, 95% CI 1.02–4.47), LBW (RR 2.19, 95% CI 1.32–3.63), VLBW (RR 3.34, 95% CI 1.08–10.35), SGA (RR 1.92, 95% CI 1.01–3.66), and VSGA (RR 2.79, 95% CI 1.04–7.47), compared with women without HIV. WHIV with antenatal ART were associated with increased risk of PTB (RR 1.35, 95% CI 1.15–1.58), LBW (RR 2.16, 95% CI 1.39–3.34), VLBW (RR 1.97, 95% CI 1.01–3.84), SGA (RR 1.77, 95% CI 1.10–2.84), and VSGA (RR 1.21, 95% CI 1.09–1.33), compared with women without HIV. Compared to ART-naive WHIV, WHIV with preconception or antenatal ART were associated with increased risk of SGA (preconception: RR 1.40, 95% CI 1.12–1.73; antenatal: RR 1.39, 95% CI 1.11–1.74) and VSGA (preconception: RR 2.44, 95% CI 1.63–3.66; antenatal: RR 2.24, 95% CI 1.48–3.40).

**Conclusion:**

Among WHIV, both preconception and antenatal initiation of ART are associated with increased risks of adverse perinatal outcomes, compared to women without HIV and ART-naive WHIV.

## Introduction

The global burden of HIV among women remains significant, with 15.5 million women of reproductive age currently living with HIV. 1.2 million women with HIV (WHIV) are pregnant every year, the vast majority (90%) in sub-Saharan Africa [1]. Since 2013, the WHO recommends that all pregnant WHIV should receive antenatal antiretroviral therapy (ART) to reduce the risk of vertical HIV transmission and improve maternal health [2]. This guidance was updated in 2015, recommending immediate initiation of ART for all WHIV, leading to a drastic increase in the number of pregnant WHIV receiving ART, from 7% in 2010 to 84% in 2023 [3,4]. Increasingly, WHIV start ART preconception, which may impact the risk of adverse perinatal outcomes, including preterm birth (PTB), low birthweight (LBW), and small for gestational age (SGA) [5].

PTB and SGA are major contributors to neonatal and child morbidity and mortality, with an estimated 13.4 million babies born preterm and 23.3 million babies born SGA annually [6–9]. Reducing neonatal and child mortality is a key global health priority, as highlighted by the United Nations Sustainable Development Goal 3 (target 3.2), which aims to reduce preventable deaths of newborns and children aged younger than 5 years [8–10].

Sub-Saharan Africa has the highest burden of neonatal and child morbidity and mortality globally, as well as the highest rates of HIV infection [8,11,12]. ART-naive WHIV who are pregnant experience an increased risk of PTB, LBW, SGA, and stillbirth compared to women without HIV [13]. A previous meta-analysis showed that pregnant WHIV who receive ART remain at increased risk of adverse perinatal outcomes, including PTB, spontaneous PTB (sPTB), LBW, term LBW, SGA, and very SGA (VSGA), compared with women without HIV [14]. WHIV receiving ART also have an increased risk of SGA and VSGA compared with ART-naive WHIV [14].

Two meta-analyses of cohort studies reported that preconception ART initiation was associated with an increased risk of PTB, very PTB (VPTB), and LBW, compared with antenatal ART initiation [15,16]. However, based on the available evidence, it is unclear whether timing of ART initiation (preconception or antenatally) reduces the risk of adverse perinatal outcomes of WHIV to the level of women without HIV. To fill this evidence gap, we conducted a systematic review and meta-analysis of observational studies reporting adverse perinatal outcomes among WHIV with preconception or antenatal ART initiation, compared with women without HIV or ART-naive WHIV.

## Methods

### Search strategy

The systematic review and meta-analysis were conducted according to Cochrane guidelines as described in our protocol (PROSPERO, CRD42021248987). A comprehensive literature search strategy was developed by a specialist librarian (S.K.) and adapted to PubMed, CINAHL (Ebscohost), Global Health (Ovid), and EMBASE (Ovid). The search included studies published between 1 January 1980 and 14 July 2023. Free text and controlled vocabulary search terms for ‘HIV’, ‘antiretroviral therapy’, and ‘pregnancy outcome’ were used. The full search terms are included in Appendix pp3–5. Both full-texts and abstracts were considered, and no restrictions on methodology, country, or language were applied. All retrieved citations were imported into EndNote reference manager (EndNote X21; Clarivate Analytics, Philadelphia, Pennsylvania, USA) and deduplicated.

### Study selection and eligibility criteria

Studies containing data on the association of WHIV with preconception or antenatal ART initiation, compared with women without HIV or ART-naive WHIV, with predefined adverse perinatal outcomes were eligible. Titles and abstracts of retrieved citations were screened and full text manuscripts of selected citations were obtained and assessed by at least two independent investigators (P.B., C.M., C.P., M.H., L.T., K.B., I.C., H.S., M.K., and Z.B.) against the eligibility criteria. Inclusion criteria were study design (prospective and retrospective cohort studies), population (pregnant women), exposure (preconception or antenatal ART initiation among WHIV), and comparator (women without HIV or ART-naive WHIV) and adverse perinatal outcomes: PTB (birth <37^+0^ weeks gestation) [9]; VPTB (birth <32^+0^ weeks gestation) [9]; sPTB (spontaneous birth <37 weeks); LBW (<2500 g); [6,7] very LBW (VLBW, <1500 g) [6,7]; SGA (birthweight for gestational age <10^th^ centile) or VSGA (birthweight for gestational age <3^rd^ centile) according to the reference chart used at the study site [17], stillbirth (newborn without any signs of life with birthweight ≥1000 g, gestational age ≥24^+0^ weeks or body length ≥35 cm) [13] and neonatal death (NND; infant death in first 28 days of life) [11]. Perinatal outcome data were not included if outcomes were undefined or not defined in line with our definitions. ART exposure was defined as receiving any combination of at least three antiretroviral drugs during pregnancy for at least 30 days. Country income status was based on World Bank country income classification at the time when the study was conducted [18]. References of included studies were assessed for additional studies. Any ambiguities were resolved by the senior investigator (J.H.).

### Data extraction

At least two investigators (P.B., C.M., C.P., M.H., L.T., K.B., I.C., H.S., M.K., and Z.B.) extracted data on study and population characteristics, ART regimens and timing of ART initiation and pregnancy outcomes from eligible studies. Outcome frequencies were extracted for each exposure comparison and perinatal outcome. Details on methods used to adjust for confounding, including regression analysis, risk factor analysis and matching, were extracted. Reported unadjusted and adjusted risk ratios (RRs), odds ratios (ORs), and 95% confidence intervals (CIs) of perinatal outcomes according to exposure comparisons were also extracted. Extracted data were reviewed by the senior investigator (J.H.).

### Quality assessment

Quality assessment was conducted using an adapted Newcastle–Ottawa scale by at least two investigators (P.B., C.M., C.P., M.H., L.T., K.B., I.C., H.S., M.K. and Z.B.) and reviewed by the senior investigator (J.H.). Nine criteria were assessed across three domains: the selection of study participants (maximum four points), comparability of groups (maximum of two points) and assessment of outcomes (maximum three points). Studies were defined as ‘good’, ‘average’ or ‘poor’ quality according to predefined criteria (Appendix pp6–8).

### Statistical analysis

Outcome frequencies were used to calculate RR and corresponding 95% CIs to assess the risk of adverse perinatal outcomes among WHIV initiating ART preconception or antenatally compared to women without HIV or ART-naive WHIV in each study. If two or more studies reported data for the same exposure comparison and perinatal outcome (e.g. PTB among WHIV receiving preconception ART compared to women without HIV), a meta-analysis was conducted. For all meta-analyses, a random-effects model was used to calculate a weighted summary effect estimate (RR) and 95% CI and were presented in forest plots. The *I*^*2*^ statistic was calculated to quantify heterogeneity because of clinical and methodological variability between studies. Subgroup analyses were conducted to assess the impact of country income status and study quality. Sensitivity analyses were conducted to investigate the impact of the adjustment for confounders on the association between timing of ART initiation for WHIV and perinatal outcomes in individual studies. Funnel plots were used to assess small study effects. For meta-analyses containing 10 or more studies, the Peters’ test was used to further assess small study effects. Statistical analyses were conducted with STATA version 18 (College Station, Texas, USA). The systematic review is reported according to Preferred Reporting Items for Systematic Reviews and Meta-Analyses (PRISMA) guidelines [19].

### Role of funding sources

This study received no funding. The corresponding author had full access to all the data in the study and had final responsibility for the decision to submit for publication.

## Results

**Fig. 1:**
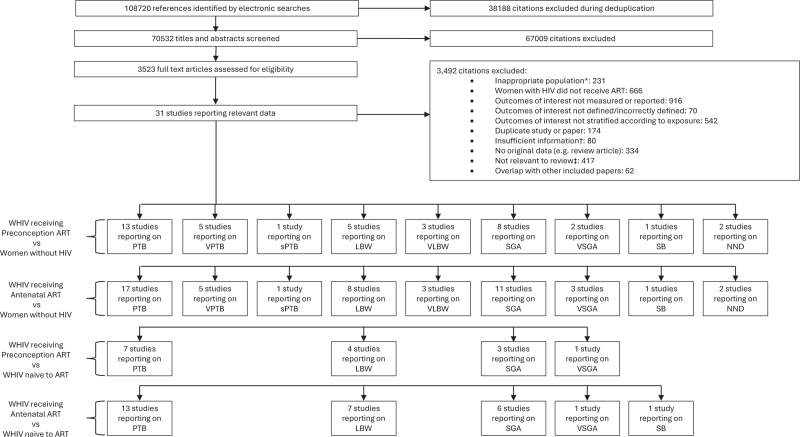
Study selection.

^∗^For example, women with HIV were not pregnant. ^†^For example, article did not provide relevant outcome data. ^‡^For example, assisted reproductive technology. ART, antiretroviral therapy (triple drug therapy); LBW, low birthweight; NND, neonatal death; PTB, preterm birth; SB, stillbirth; SGA, small for gestational age; sPTB, spontaneous preterm birth; VLBW, very low birthweight; VPTB, very preterm birth; VSGA, very small for gestational age; WHIV, women with HIV.

Our literature search yielded 108 720 citations, of which 31 studies were included [20–50]. The numbers of studies reporting each perinatal outcome for the comparisons of WHIV with preconception or antenatal initiation of ART with women without HIV or ART-naive WHIV are shown in Fig. 1.

The characteristics of each included study are summarized in Table 1. Fifteen prospective (48%) and 16 (52%) retrospective studies reported data from 199 156 women in 19 countries (Table 1). Twenty-five studies (81%) including 182 847 (92%) women took place in low-and-middle-income countries (LMICs), whereas five studies (19%) with 16 309 (8%) women took place in high-income countries (HICs). Twenty-four studies (77%) reported methods used to assess potential confounding factors including regression analysis, risk factor analysis, and matching (Table 1, Appendix pp13–16). Five studies reported 20 analyses adjusting for confounders, none of which resulted in a change in the significance of the effect estimate (Appendix pp44–45). Quality assessment identified one (3%) good-quality, 18 (58%) average-quality and 12 (39%) poor-quality studies (Table 1, Appendix pp9–12). Study quality was similar of studies conducted in LMICs (4% good, 56% average, 40% poor) and in HICs (67% average, 33% poor).

**Table 1:** Characteristics of studies included in the systematic review and meta-analysis.

Study: first author (year) [ref]	Country	Country income status^a^	Cohort study design	Recruitment period	Number of women analysed	Population characteristics	Method to correct for confounders	Method to estimate gestational age	Quality assessment
Adam (2016) [20]	Sudan	Middle income	Retrospective	January 2009 to December 2013	78	Women recruited from maternity hospital, urban and rural setting	Risk factor analysis	Unspecified	Average
Bailey (2013) [21]	Ukraine	Middle income	Retrospective	2008 to 2010	3535	First born twin included, all hospital deliveries, 14.7% history of IDU	None	LNMP and ultrasound (unspecified)	Poor
Bengtson (2020) [22]	South Africa	Middle income	Prospective	March 2013 to August 2015	1116	Twins excluded, women recruited from antenatal care clinics in Gugulethu Cape Town, urban setting, 17.2% alcohol use	None	Ultrasound (unspecified), LMP or fundal height	Poor
Chen (2012) [23]	Botswana	Middle income	Retrospective	1 May 2009 to 30 April 2011	33 148	First born twin included, all hospital deliveries, 5.3% alcohol use, 1.7% smoking	Regression analysis, risk factor analysis	LNMP, SFH or ultrasound (unspecified)	Average
Dadabhai (2019) [24]	Malawi	Low income	Prospective	January 2016 to September 2017	1299	Twins excluded, 96% of deliveries occurred in healthcare facilities, urban setting	Regression analysis	Ballard score and LMP	Average
Djeha (2019) [25]	Canada	High income	Prospective	January 2003 to December 2016	159	Urban setting, 9.4% smoking	None	First trimester ultrasound or LMP	Average
Ekouevi (2008) [26]	Cote d’Ivoire	Low income	Prospective	March 2001 to July 2003 and August 2003 to August 2007	358	Twins excluded, recruited from antenatal clinics, urban setting	Regression analysis	Unspecified	Average
Goetghebuer (2019) [27]	Belgium	High income	Prospective	December 2010 to November 2013	255	Women recruited from hospital antenatal clinic, urban setting, 9.2% smoking, 10.1% alcohol	Risk factor analysis	Ballard score	Average
Hu (2019) [28]	China	Middle income	Prospective	October 2009 to May 2018	585	Twins excluded, recruited through integrated prevention of MTCT programme, urban setting	Regression, risk factor analysis	First or second trimester ultrasound, if unable to assess ultrasound LMP used	Average
Kowalska (2003) [29]	Poland	Middle income	Prospective	January 1995 to February 2003	102	Twins included, recruited from an outpatient HIV clinic, 47.1% IDU	Risk factor analysis	Unspecified	Poor
Li (2016) [30]	Tanzania	Low income	Prospective	November 2004 to September 2011	3314	Recruited from hospitals, health centres and dispensaries, urban setting	Risk factor analysis	LNMP and SFH	Poor
Li (2020) [31]	China	Middle income	Prospective	October 2014 to September 2017	1449	Twins excluded, women enrolled from midwifery hospitals	Regression, risk factor analysis	LMP or ultrasound (unspecified)	Average
Malaba (2017) [32]	South Africa	Middle income	Prospective	April 2013 to August 2015	1793	Twins excluded, large community-based public sector primary care facility	Regression, risk factor analysis	LNMP and SFH	Average
Malaba (2021) [33]	South Africa	Middle income	Prospective	April 2015 to October 2016	3952	Twins excluded, enrolled at first antenatal clinic in Cape town	Regression analysis	LMP, SFH and/or ultrasound (unspecified)	Average
Marazzi (2011) [34]	Malawi and Mozambique	Low income	Retrospective	July 2005 to June 2009	3273	Twins included, women recruited from DREAM centres	Regression analysis	LNMP and clinical exam (unspecified)	Average
Moodley (2016) [35]	South Africa	Middle income	Retrospective	July 2011 to December 2011 and January 2014 to June 2014	9847	Twins excluded, data abstracted from maternity registers of a regional hospital in Durban, South Africa	Regression analysis, risk factor analysis	LNMP and/or ultrasound (unspecified)	Average
Olagbuji (2010) [36]	Nigeria	Middle income	Prospective	January 2007 to December 2008	406	Twins excluded, recruited from a tertiary referral centre, all women delivered in a healthcare facility	Risk factor analysis	Unspecified	Poor
Ramokolo (2017) [37]	South Africa	Middle income	Retrospective	October 2012 to May 2013	8778	Twins excluded, recruited from primary health facilities	Risk factor analysis	LNMP	Average
Rempis (2017) [38]	Uganda	Low income	Retrospective	February 2013 to December 2013	412	Twins excluded, all delivered in a private referral hospital	Risk factor analysis	Unspecified	Poor
Rubin (2011) [39]	Switzerland	High income	Prospective	1984 to 2007	1040	Twins excluded, 22% smoking, 26% IDU	None	Unspecified	Poor
Santosa (2019) [40]	South Africa	Middle income	Prospective	28 May 2013 to 20 July 2016	633	Twins excluded, women recruited from hospital in urban setting, 98.7% hospital deliveries, 6.4% smoking, 8.2% alcohol	Regression, risk factor analysis	Ultrasound <14 weeks	Good
Saums (2019) [41]	USA	High income	Retrospective	2011 to 2018	3729	Women recruited from hospital, urban setting, all hospital deliveries, 11.5% smoking, 2.9% alcohol, 13.4% IDU	Risk factor analysis	Unspecified	Average
Sebitloane (2017) [42]	South Africa	Middle income	Retrospective	1 April 2011 to 30 April 2014	1461	Twins excluded, women recruited and delivered at a regional hospital, urban setting	None	Unspecified	Poor
Short (2014) [43]	United Kingdom	High income	Retrospective	1996 to 2010	331	Twins included, 13.0% smoking, recruited from an HIV antenatal clinic, all women delivered in a tertiary hospital, urban setting	None	Unspecified	Poor
Silverman (2010) [44]	Zambia	Low income	Retrospective	Unspecified	1238	Twins included	Risk factor analysis	Unspecified	Poor
Snijdewind (2018) [45]	Netherlands	High income	Retrospective	January 1997 to February 2015	10 795	Twins excluded, women recruited from 26 nationwide sites, 10.8% smoking, 11.7% alcohol use, 0.6% IDU,	Regression, risk factor analysis	LNMP and/or ultrasound (unspecified)	Average
Tan (2023) [46]	China	Middle income	Retrospective	January 2004 to December 2021	1010	Twins excluded	Regression analysis	Unspecified	Average
Tiam (2019) [47]	Lesotho	Middle income	Prospective	June 2014 to February 2016	1594	91.6% delivered in a health facility, enrolment in 14 mixed setting study centres across 3 districts	None	LMP	Poor
Yu (2012) [48]	China	Middle income	Retrospective	June 2006 to July 2010	194	Twins excluded, 8.8% IDU	Risk factor analysis	Unspecified	Poor
Zash (2017) [49]	Botswana	Middle income	Retrospective	15 August 2014 to 15 August 2016	46 267	Twins excluded, 6.3% alcohol consumption or smoking, obstetric records extracted at 8 national government hospitals	Regression analysis	LNMP and/or ultrasound (unspecified) or fundal height	Average
Zash (2018) [50]	Botswana	Middle income	Retrospective	15 August 2014 to 15 August 2016	57 005	Twins excluded, women recruited from 8 government hospitals, all hospital deliveries, 8.3% alcohol or smoking in pregnancy	Regression analysis	LNMP	Average

Details on the inclusion of twins, recruitment centre, urban/rural setting, deliveries at home/hospital, smoking, alcohol use, and IDU were sought and reported here if provided by each study. IDU, illicit drug use; LMP, last menstrual period; LNMP, last normal menstrual period; MTCT, mother-to-child transmission; SFH, symphysio-fundal height.

a Based on the World Bank country income classification at the time of the study.

Data on ART regimens, timing of ART initiation, comparator groups and perinatal outcomes reported by each study are summarized in Table 2. Twenty (65%) studies reported data for preconception ART initiation and 30 (97%) studies reported data for antenatal ART initiation. Nineteen studies (61%) included data from women without HIV and 16 (52%) studies reported data from ART-naive WHIV. Fourteen studies (45%) reported outcome data for WHIV receiving predominantly nonnucleoside reverse transcriptase inhibitor(NNRTI)-based ART, 4 (13%) studies reported data for WHIV receiving predominantly protease inhibitor(PI)-based ART and 13 (42%) reported data for WHIV receiving a mixture of ART regimens.

**Table 2:** Antiretroviral therapy regimens, timing of antiretroviral therapy initiation and comparators, and perinatal outcomes of included studies.

Study: first author (year) [ref]	ART regimens	WHIV with preconception ART vs. women without HIV	WHIV with antenatal ART vs. women without HIV	WHIV with preconception ART vs. ART-naive WHIV	WHIV with antenatal ART vs. ART-naive WHIV	Perinatal outcomes
Adam (2016) [20]	100% ZDV-3TC-based ART (third drug unspecified)	No	Yes	No	No	PTB
Bailey (2013) [21]	91% PI-based ART (ZDV-3TC-LPV/r) 9% unspecified ART	No	No	Yes	Yes	PTB
Bengtson (2020) [22]	100% NNRTI-based ART (TDF-FTC/3TC-EFV)	No	Yes	No	No	PTB, SGA, VSGA
Chen (2012) [23]	87% NNRTI-based ART (ZDV-3TC-NVP), 9% PI-based ART (ZDV-3TC-LPV/r), 4% unspecified ART	Yes	Yes	Yes	Yes	PTB, SGA, NND
Dadabhai (2019) [24]	100% NNRTI-based ART (TDF-3TC-EFV)	Yes	Yes	No	No	PTB, LBW, preterm LBW, SGA, VSGA
Djeha (2019) [25]	86% PI-based, 14% unspecified ART	No	No	No	Yes	PTB, SGA
Ekouevi (2008) [26]	87% ZDV-3TC-NVP, 13% d4T- 3TC-NVP	No	No	Yes	Yes	LBW
Goetghebuer (2019) [27]	67% PI-based ART, 21% NNRTI-based ART, 9% NRTI-based ART, 4% unspecified ART	Yes	Yes	No	No	PTB, LBW
Hu (2019) [28]	59% PI-based ART, 41% NNRTI-based ART	No	No	No	Yes	PTB, SGA
Kowalska (2003) [29]	39% PI-based ART, 61% non-PI-based ART	No	No	Yes	Yes	PTB
Li (2016) [30]	94% NNRTI-based ART (85.1% ZDV-3TC-NVP, 11.7% d4T-3TC-NVP, 3.2% ZDV-3TC-EFV), 6% unspecified ART	No	No	Yes	Yes	PTB, LBW, SGA, VSGA
Li (2020) [31]	100% unspecified ART	No	Yes	No	Yes	PTB, LBW, SGA, SB
Malaba (2017) [32]	71.6% NNRTI-based ART (75% TDF-3TC-EFV, 25% TDF-3TC-NVP), 2.3% PI-based ART, 26.1% unspecified ART	Yes	Yes	No	No	PTB, VPTB, LBW, VLBW, SGA
Malaba (2021) [33]	95% TDF/3TC/EFV, 3% other NNRTI-based ART, 1% PI-based ART, 1% unspecified ART	Yes	Yes	No	No	PTB, VPTB, sPTB, SGA
Marazzi (2011) [34]	55% ZDV- based ART, 45% d4T-based ART	No	No	No	Yes	PTB
Moodley (2016) [35]	100% NNRTI-based ART (65% EFV-TDF-FTC, 35% d4T-3TC-NVP)	No	Yes	No	Yes	PTB, LBW, SGA
Olagbuji (2010) [36]	100% NNRTI-based ART (ZDV-3TC-NVP)	No	Yes	No	No	LBW
Ramokolo (2017) [37]	100% NNRTI-based ART (TDF-3TC/FTC-NVP)	Yes	Yes	Yes	Yes	PTB, LBW, SGA
Rempis (2017) [38]	100% NNRTI-based ART (TDF-3TC-EFV)	Yes	Yes	No	No	PTB, SGA
Rubin (2011) [39]	84% PI-based ART, 16% non-PI-based ART	No	No	Yes	Yes	PTB, VPTB
Santosa (2019) [40]	97.5% NNRTI ART (91.7% TDF-FTC/3TC-EFV, 4.2% other EFV-based ART, 1.6% NVP-based ART) 2.5% PI-based ART	Yes	Yes	No	No	PTB, VPTB, LBW, VLBW, SGA, VSGA, SB, NND
Saums (2019) [41]	54.7% PI-based ART, 34.3% INSTI-based ART, 10.9% NNRTI-based ART	Yes	Yes	No	No	PTB
Sebitloane (2017) [42]	100% NNRTI-based ART,	Yes	Yes	No	No	PTB
Short (2014) [43]	17.8% PI or NVP-based ART, 1.5% NNRTI-based ART, 80.7% unspecified ART	No	No	Yes	Yes	PTB
Silverman (2010) [44]	100% PI-based ART (ZDV-3TC-LPV/r)	No	No	No	Yes	LBW
Snijdewind (2018) [45]	66.7% PI-based ART, 31.5% NNRTI-based ART, 1.8% other ART	Yes	Yes	No	No	PTB, VPTB, LBW, VLBW, SGA
Tan (2023) [46]	59.6% NNRTI-based ART, 31.2% PI-based ART	No	No	Yes	Yes	PTB, LBW, SB
Tiam (2019) [47]	98.6% NNRTI-based ART, 2.4% unspecified ART	Yes	Yes	No	No	PTB, LBW, VLBW
Yu (2012) [48]	100% NNRTI-based ART (ZDV/d4T-3TC-NVP)	No	No	No	Yes	PTB, LBW
Zash (2017) [49]	87.3% NNRTI-based ART, 3.8% PI-based ART, 8.8% unspecified ART	Yes	No	No	No	PTB, VPTB, SGA, VSGA
Zash (2018) [50]	72.7% NNRTI-based ART (TDF-FTC-EFV), 27.3% INSTI-based ART (TDF-FTC-DTG)	No	Yes	No	No	PTB, VPTB, SGA, VSGA

ART, antiretroviral therapy; INSTI, integrase strand transfer inhibitor; NNRTI, nonnucleoside reverse transcriptase inhibitor; PI, protease inhibitor. Drug abbreviations: 3TC, lamivudine; D4T, stavudine; DTG, dolutegravir; EFV, efavirenz; FTC, emtricitabine; LPV/r, lopinavir/ritonavir; NVP, nevirapine; TDF, tenofovir disoproxil fumarate; ZDV, zidovudine. Outcome abbreviations: LBW, low birthweight; NND, neonatal death; PTB, preterm birth; SB, stillbirth; SGA, small for gestational age; sPTB, spontaneous preterm birth; VLBW, very low birthweight; VPTB, very preterm birth; VSGA, very small for gestational age.

Perinatal outcomes for WHIV initiating ART preconception or antenatally were compared to women without HIV or ART-naive WHIV (Fig. 2 , Appendix pp16–29). Subgroup analyses were conducted according to country income status (Appendix pp30–36) and study quality (Appendix pp37–43).

**Fig. 2:**
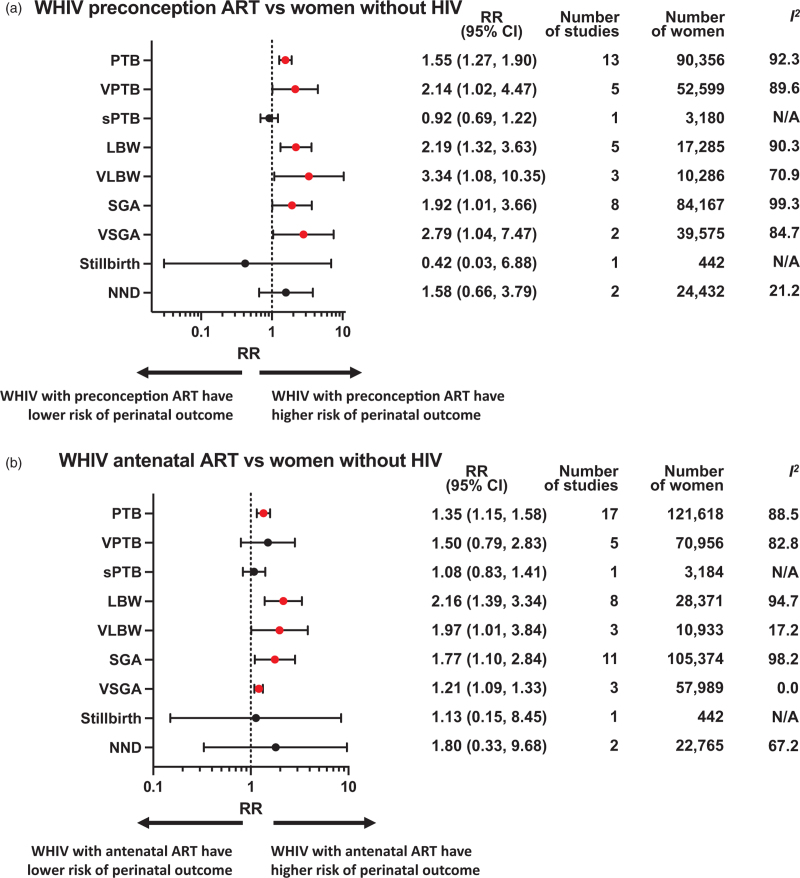
Perinatal outcomes of women with HIV receiving preconception or antenatal ART compared to HIV-negative women or antiretroviral therapy-naive women with HIV.

Adverse perinatal outcomes associated with WHIV receiving preconception ART compared to women without HIV (a), WHIV receiving antenatal ART compared to women without HIV (b), WHIV receiving preconception ART compared to ART-naive WHIV (c), WHIV receiving antenatal ART compared to ART-naive WHIV (d). Results of individual studies or, when two or more studies were available, random-effects meta-analyses are presented. Risk ratios (RR) and 95% confidence intervals (CI), numbers of studies and women included in the analysis of each perinatal outcome, and *I*^*2*^ value for heterogeneity are displayed. Statistically significant (*P* < 0.05) effect estimates are presented with red dots and nonsignificant effects with black dots. Forest plots of the meta-analyses for each perinatal outcome can be found in Appendix pp16-29. ART, antiretroviral therapy (triple drug therapy); LBW, low birthweight; NND, neonatal death; PTB, preterm birth; SGA, small for gestational age; sPTB, spontaneous preterm birth; VLBW, very low birthweight; VPTB, very preterm birth; VSGA, very small for gestational age; WHIV, women with HIV.

**Fig. 2 (Continued):**
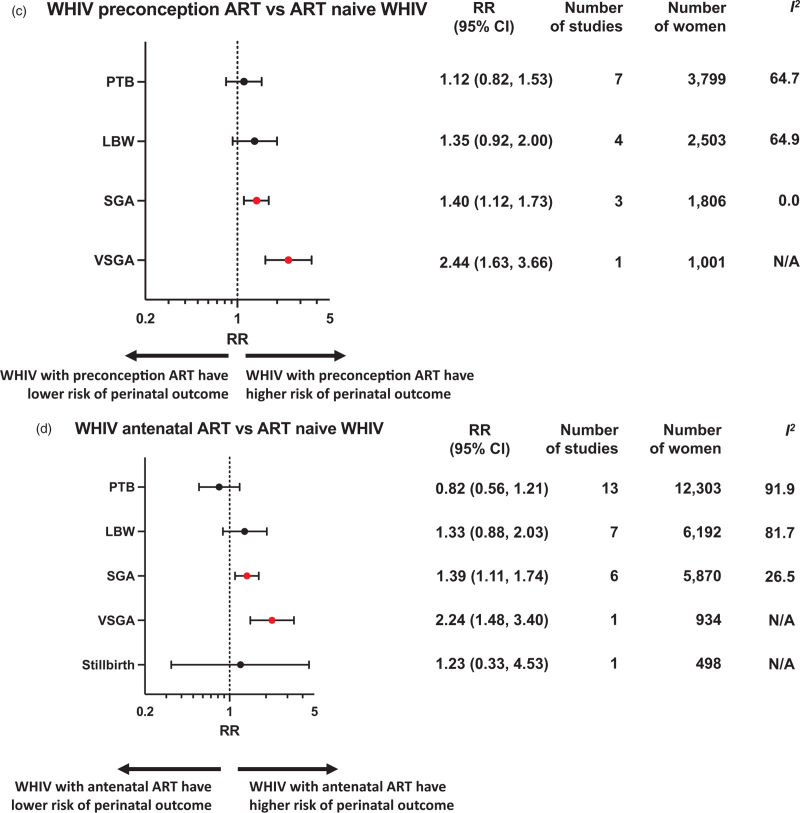
Perinatal outcomes of women with HIV receiving preconception or antenatal ART compared to HIV-negative women or antiretroviral therapy-naive women with HIV.

Adverse perinatal outcomes associated with WHIV receiving preconception ART compared to women without HIV (a), WHIV receiving antenatal ART compared to women without HIV (b), WHIV receiving preconception ART compared to ART-naive WHIV (c), WHIV receiving antenatal ART compared to ART-naive WHIV (d). Results of individual studies or, when two or more studies were available, random-effects meta-analyses are presented. Risk ratios (RR) and 95% confidence intervals (CI), numbers of studies and women included in the analysis of each perinatal outcome, and *I*^*2*^ value for heterogeneity are displayed. Statistically significant (*P* < 0.05) effect estimates are presented with red dots and nonsignificant effects with black dots. Forest plots of the meta-analyses for each perinatal outcome can be found in Appendix pp16-29. ART, antiretroviral therapy (triple drug therapy); LBW, low birthweight; NND, neonatal death; PTB, preterm birth; SGA, small for gestational age; sPTB, spontaneous preterm birth; VLBW, very low birthweight; VPTB, very preterm birth; VSGA, very small for gestational age; WHIV, women with HIV.

### Preterm birth

A meta-analysis of 13 studies, including 90 356 women, showed that WHIV who initiated ART preconception were associated with an increased risk of PTB when compared to women without HIV (RR 1.55, 95% CI 1.27–1.90; *I*^*2*^ = 92.3%) (Fig. 2 a). Women who initiated ART antenatally also had an increased risk of PTB when compared to women without HIV in a meta-analysis of 17 studies including 121 618 women (RR 1.35, 95% CI 1.15–1.58; *I*^*2*^ = 88.5%) (Fig. 2 b). Subgroup analysis for studies conducted in LMICs found an increased risk of PTB among WHIV with both preconception (RR 1.42, 95% CI 1.23–1.64) and antenatal (RR 1.24, 95% CI 1.10–1.39) ART initiation, compared to women without HIV (Appendix pp30–36). These finding were significant in average-quality and poor-quality studies (Appendix pp37–43). Peters’ test for small study affect was not significant (*P* = 0.3448) for preconception ART initiation but was significant for antenatal ART initiation (*P* = 0.001).

There was no difference in the risk of PTB for WHIV who initiated ART preconception (seven studies including 3799 women) or during the antenatal period (13 studies including 12 303 women), when compared to ART-naive WHIV (Fig. 2 c and d).

### Very preterm birth

WHIV who initiated ART preconception were associated with an increased risk of VPTB compared with women without HIV (RR 2.14, 95% CI 1.02–4.47; *I*^*2*^ = 89.6%) in a meta-analysis of five studies including 52 599 women (Fig. 2 a). Subgroup analysis by country income status for WHIV with preconception ART demonstrated a significant increase in risk of VPTB in LMICs (RR 1.34, 95% CI 1.18–1.52) (Appendix pp30–36). No association with VPTB was found for WHIV with antenatal ART initiation, compared to women without HIV (Fig. 2 b).

No studies compared the risk of VPTB for WHIV who initiated ART preconception or antenatally, compared to ART-naive WHIV (Fig. 2 c and d).

### Spontaneous preterm birth

A single good-quality study from an LMIC found no increased risk of sPTB for WHIV who initiated ART preconception (RR 0.92, 95% CI 0.69–1.22) or antenatally (RR 1.08, 95% CI 0.83–1.41) when compared to women without HIV (Fig. 2 a and b).

No studies compared the risk of sPTB for WHIV with preconception or antenatal ART initiation, compared to ART-naive WHIV (Fig. 2 c and d).

### Low birthweight

A meta-analysis of five studies, including 17 285 women, found an increased risk of LBW for WHIV receiving preconception ART when compared to women without HIV (RR 2.19, 95% CI 1.32–3.63; *I*^*2*^ = 90.3%) (Fig. 2 a). For WHIV initiating ART antenatally, a meta-analysis of eight studies including 28 371 women, also found an increased risk of LBW compared to women without HIV (RR 2.16, 95% CI 1.39–3.34; *I*^*2*^ = 94.7%) (Fig. 2 b). Subgroup analysis of studies conducted in LMICs showed an increased risk of LBW among WHIV receiving both preconception (RR 1.41, 95% CI 1.18- 1.69) and antenatal (RR 1.86, 95% CI 1.12–3.07) ART initiation compared to women without HIV (Appendix pp30–36).

When compared to ART-naive WHIV, we found no difference in risk of LBW for WHIV who initiated ART preconception or antenatally (Fig. 2 c and d).

### Very low birthweight

A meta-analysis of three studies, including 10 286 women, found an increased risk of VLBW for WHIV with preconception (RR 3.34, 95% CI 1.08–10.35; *I*^*2*^ = 70.9%) and antenatal (RR 1.97, 95% CI 1.01–3.84; *I*^*2*^ = 17.2%) initiation of ART, compared with women without HIV (Fig. 2 a and b).

No studies were found assessing the risk of VLBW for WHIV receiving preconception or antenatal ART, compared with ART-naive WHIV.

### Small for gestational age

A meta-analysis of eight studies, including 84 167 women, found that WHIV receiving preconception ART were associated with an increased risk of SGA compared to women without HIV (RR 1.92, 95% CI 1.01–3.66; *I*^*2*^ = 99.3%). A meta-analysis of 11 studies with 105 374 women also found that WHIV with antenatal ART initiation were associated with an increased risk of SGA compared to women without HIV (RR 1.77, 95% CI 1.10–2.84; *I*^*2*^ = 98.2%) (Fig. 2 a and b).

Subgroup analysis of studies conducted in LMICs demonstrated an increased risk of SGA for WHIV receiving preconception ART (RR 1.51, 95% CI 1.20–1.89) and antenatal ART (RR 1.43, 95% CI 1.16–1.77), compared to women without HIV (Appendix pp30–36). Analysis of good-quality studies found an increased risk of SGA for WHIV with preconception ART (RR 1.83, 95% CI 1.02–3.28) but not antenatal ART, compared to women without HIV (Appendix pp37–43).

When compared to ART-naive WHIV, women receiving both preconception ART (three studies including 1806 women; 1.40, 1.12–1.73; *I*^*2*^ = 0%) and antenatal ART (six studies including 5870 women; 1.39, 1.11–1.74; *I*^*2*^ = 26.5%) were associated with an increased risk of SGA (Fig. 2 c and d).

Subgroup analysis of studies conducted in LMICs demonstrated an increased risk of SGA for WHIV with both preconception ART initiation (RR 1.40, 95% CI 1.13–1.74) and antenatal ART initiation (RR 1.39, 95% CI 1.10–1.79), compared to ART-naive WHIV (Appendix pp30–36).

### Very small for gestational age

A meta-analysis of two studies, including 39 575 women in LMICs, found an increased risk of VSGA for WHIV receiving preconception ART, compared to women without HIV (RR 2.79, 95% CI 1.04–7.47; *I*^*2*^ = 84.7%) (Fig. 2 a). Women who initiated ART antenatally also had an increased risk of VSGA compared to women without HIV in a meta-analysis of three studies with 57 989 women in LMICs (RR 1.21, 95% CI 1.09–1.33; *I*^*2*^ = 0%) (Fig. 2 b).

Analysis of a single, poor-quality study conducted in a LMIC found that the risk of VSGA was increased for WHIV who initiated ART preconception compared to ART-naive WHIV (RR 2.44, 95% CI 1.63–3.66) (Fig. 2 c). Likewise, analysis of a single study with 934 women found that WHIV who initiated ART antenatally were associated with an increased risk of VSGA compared to ART-naive WHIV ((RR 2.24, 95% CI 1.48–3.40) (Fig. 2 d).

### Stillbirth

There was no difference in risk of stillbirth for WHIV who initiated ART preconception or antenatally, when compared to women without HIV, in a single, small study (*n* = 442) (Fig. 2 a and b). WHIV with antenatal initiation of ART were also not associated with a difference in risk of stillbirth compared to ART-naive WHIV (Fig. 2 d).

### Neonatal death

Analysis of two studies, including 24 432 women, showed no difference in risk of NND for WHIV receiving preconception or antenatal ART, compared to women without HIV (Fig. 2 a and b).

No studies assessed the risk of NND for WHIV receiving antenatal and preconception ART, compared to ART-naive WHIV.

## Discussion

This systematic review and meta-analysis found that WHIV who initiated ART preconception or antenatally are associated with an increased risk of PTB, LBW, VLBW, SGA and VSGA, compared to HIV-negative women. WHIV who initiated ART preconception are also associated with an increased risk of VPTB, compared women without HIV. When compared to ART-naive WHIV, WHIV who initiated ART preconception or antenatally are associated with an increased risk of SGA and VSGA.

The results of our analyses are consistent with previous meta-analyses analysing the associations of ART in WHIV with adverse perinatal outcomes [14,15,51]. The meta-analysis conducted by Portwood *et al.*[14] in 2022 found that pregnant WHIV who received ART were associated with an increased risk of PTB, LBW, SGA and VSGA compared to women without HIV. A previous meta-analysis found that WHIV receiving preconception ART had a higher risk of PTB than WHIV receiving antenatal ART; our analysis showed that WHIV receiving either preconception or antenatal ART remain at increased risk of PTB when compared to women without HIV, although the effect estimate for preconception ART initiation was higher than for antenatal ART initiation [15]. Finally, our results are in line with previous meta-analyses, which found that WHIV receiving ART were associated with increased risks of SGA and VSGA compared to ART-naive WHIV [14].

The impact of the different ART regimens used in the included studies is uncertain. A previous meta-analysis found that WHIV who received PI-based ART had an increased risk of SGA and VSGA, but no other outcomes, compared to NNRTI-based ART [52,53]. No differences in perinatal outcomes were found in other comparisons of PI-based ART, INSTI-based ART, and NNRTI-based ART [52,53]. Moreover, another meta-analysis reported that risks of adverse perinatal outcomes were elevated among WHIV compared to women without HIV regardless of drug class [54].

The majority of the studies included in our review contain data from women receiving NNRTI-based ART (45%) or mixed ART regimens (42%). Four studies were composed of WHIV receiving predominantly PI-based ART, and these only affected the comparisons with ART-naive WHIV. Only two studies included a minority of WHIV who received INST-based ART (the current first-line regimen), which were compared to women without HIV. The limited available data on perinatal outcomes according to timing of ART initiation of different clearly defined ART regimens precluded a subgroup analysis according to ART regimen.

Our study has several strengths. Our systematic review and meta-analysis included 199 156 women in 19 countries and is the first study to compare WHIV receiving either preconception and antenatal ART with either women without HIV or ART-naive WHIV. The comprehensive literature search strategy used in the systematic review ensured all relevant studies were included in this meta-analysis. Most of the included studies were from LMICs, which enhances external validity of our findings. Indeed, most of the results found in our overall analyses were confirmed in the subgroup of studies conducted in LMICs. Our study was conducted in line with Cochrane and PRISMA guidelines. All outcome variables were defined *a priori* and strictly applied to reduce misclassification bias. We conducted several subgroup and sensitivity analyses, and adjustment for potential confounders did not change the significance of associations.

This study had some limitations. This systematic review and meta-analysis assessed observational data, rather than data from randomized controlled trials (RCTs), and as such has a higher risk of bias and confounding. We did not include RCTs in our study as these recruit participants during pregnancy, not preconception, and do not have HIV-negative or ART-naive comparator arms. Cohort studies provide valuable insight into more pragmatic initiation and use of ART regimens experienced by WHIV. However, timing of ART initiation may lead to selection bias, as women who start ART antenatally will have less time to experience adverse birth outcomes than WHIV who started ART preconception [55]. This may explain why the risk of PTB was slightly higher for WHIV who initiated ART preconception compared to WHIV who initiated ART antenatally. Furthermore, WHIV who start ART late in pregnancy may differ from women who start ART preconception, including differences in access to care and maternal health, which may introduce confounding.

The pathophysiology underlying adverse perinatal outcomes of WHIV receiving ART are not clearly understood. Pregnancy is a complicated immunological process, which may be impacted by CD4^+^ depletion and chronic immune activation associated with HIV infection [56]. This may be due to the residual impact of the rapid depletion of innate lymphoid cells during acute HIV infection, which may not be reversed by ART [57]. In contrast to women without HIV, CD4^+^ cells continue to decline during the third trimester and postpartum in WHIV who do not receive ART [58]. Antenatal ART initiation has been shown to result in improvement of CD4^+^ cell count and CD4^+^/CD8^+^ ratios in WHIV [59]. Moreover, WHIV with a low CD4^+^/CD8^+^ ratio at the beginning of pregnancy were associated with an increased risk of PTB and were less likely to have undetectable HIV-RNA levels at the end of pregnancy [59]. A further hypothesis suggests imbalances in peripheral mucosal-associated invariant T cells [60,61]. Finally, studies have shown that antenatal exposure to PI-based ART was associated with uteroplacental and decidual dysfunction, decreased progesterone levels and alterations in oestradiol and prolactin during pregnancy, which correlate with adverse birth outcomes [62–64].

There are clear benefits of immediate initiation of ART for the prevention of vertical HIV transmission and the promotion of maternal health for WHIV of childbearing potential and who are pregnant. Our study shows that WHIV initiating ART preconception or antenatally remain at increased risk of a broad range of adverse perinatal outcomes compared to women without HIV. There is an urgent need for more well conducted prospective observational studies of perinatal outcomes among pregnant WHIV receiving different ART drugs and regimens. This is particularly important for individual INSTI-based ART drugs and regimens, including dolutegravir, raltegravir, bictegravir and elvitegravir, as well as long-acting injectable cabotegravir, and novel therapies such as first-in-class HIV-1 capsid inhibitor lenacapavir, and monoclonal antibodies [65]. It is essential that studies collect and report detailed information about ART regimens, timing of ART initiation, and outcomes, and correct for potential confounders. Finally, more research is needed to better understand the underlying mechanisms, which may lead to adverse outcomes in order to develop preventive and therapeutic interventions.

## Acknowledgements

Contributors: P.B., C.M., C.P., M.H., L.T., K.B., I.C., H.S., M.K. and Z.B. screened the literature search results for relevant manuscripts and assessed their eligibility, verified and extracted data and conducted methodological quality assessments. P.B. selected relevant studies, conducted the meta-analyses, subgroup and sensitivity analyses, interpreted the data and wrote the first draft of the manuscript. S.K. designed and conducted the literature search. J.H. conceived, designed and coordinated the study, developed the systematic review protocol, assisted with the literature search, assessment of eligibility of manuscripts, data extraction, and methodological quality assessment, designed the meta-analysis plan, interpreted the data and wrote the manuscript. All authors had full access to all the data in the study and had final responsibility for the decision to submit for publication.

### Conflicts of interest

There are no conflicts of interest.

## Supplementary Material

Supplemental Digital Content
