# Supplementary material for: Perinatal outcomes among pregnant women with HIV initiating antiretroviral therapy preconception and antenatally
Source: AIDS. 2025 Jan 9;39(5):584–96. doi: 10.1097/QAD.0000000000004104 (PMC11902611; doi:10.1097/QAD.0000000000004104)

Appendix

Perinatal outcomes among pregnant women living with HIV initiating antiretroviral therapy preconception and antenatally: systematic review and meta-analysis.

Table of Contents

Appendix 1 ..... **3**
Literature search strategies 3

Appendix 2 ..... **6**
Quality assessment of studies 6

Appendix 2.1. Adapted Newcastle-Ottawa quality assessment tool **6**

Appendix 2.2 ..... **8**
Classification of studies according to quality assessment 8

Appendix 2.3 ..... **9**
Quality assessment of studies included in the systematic review and meta-analysis 9

Appendix 2.4 ..... **13**
Confounding factors adjusted for in included studies 13

Appendix 3 ..... **16**
Random-effects meta-analyses of risk of perinatal outcomes associated with timing of initiation of ART for women living with HIV. 16

Appendix 3.1 ..... **16**
Women living with HIV receiving preconception ART vs HIV- negative women 16

Appendix 3.2 ..... **21**
Women living with HIV receiving antenatal ART vs HIV- women 21

Appendix 3.3 ..... **25**
Women living with HIV receiving preconception ART vs women living with HIV naïve to ART 25

Appendix 3.4 ..... **27**
Women living with HIV receiving antenatal ART vs women living with HIV naïve to ART 27

Appendix 4 ..... ***30***

Subgroup analyses **30**

Appendix 4.1 ..... **30**
Subgroup analysis by country income status: risk ratios 30

Appendix 4.2 ..... **32**
Subgroup analysis by country income status: number of studies and women analysed 32

Appendix 4.3 ..... **34**
Subgroup analysis by country income status: I^2 values 34

Appendix 4.4 ..... **36**
Subgroup analysis by country income status: test of subgroup difference 36

Appendix 4.5 ..... **37**
Subgroup analysis by study quality: risk ratios 37

Appendix 4.6 ..... **39**
Subgroup analysis by study quality: Number of studies and women analysed 39

Appendix 4.7 ..... **41**
Subgroup analysis by study quality: I^2 values 41

Appendix 4.8 ..... **43**
Subgroup analysis by study quality: test of subgroup difference (interaction) 43

Appendix 5: Sensitivity analyses ***44***

Appendix 6: Funnel plots ***46***
Appendix 6.1: Women living with HIV receiving preconception ART vs HIV-negative women 46
Appendix 6.2: Women living with HIV receiving antenatal ART vs HIV-negative women 49
Appendix 6.3: Women living with HIV receiving preconception ART vs women living with HIV naïve to ART 53

Appendix 6.4: Women living with HIV receiving antenatal ART vs women living with HIV naïve to ART 55

Appendix 1

Literature search strategies

Search strategy for “pregnancy outcomes” AND “HIV” OR “ARVs”.

Database and platform: MEDLINE (Ovid MEDLINE® Epub Ahead of Print, In-Process & Other Non-Indexed Citations, Ovid MEDLINE® Daily and Ovid MEDLINE®) 1946 to present (via OVID)

Last search date: 14 July 2023

1. Pregnancy Outcome/ or exp Pregnancy Complications, Infectious/

2. ((pregnancy or gestational or fetal or foetal or obstetric$) adj1 (outcome$ or complication$ or consequence$ or characteristic$ or event$ or result$ or problem$ or morbidit$ or sequelae)).ti,ab.

3. ((labor or labour or birth or delivery or neonate or newborn or "new-born" or "new born") adj1 (outcome$ or complication$ or consequence$ or characteristic$ or event$ or result$ or problem$ or morbidit$ or sequelae)).ti,ab.

4. ((infant or reproductive or prelabour or prelabor or "pre-labour" or "pre-labor" or intrauterine or "intra-uterine") adj1 (outcome$ or complication$ or consequence$ or characteristic$ or event$ or result$ or problem$ or morbidit$ or sequelae)).ti,ab.

5. ((antenatal or "ante-natal" or prenatal or "pre-natal" or perinatal or "peri-natal" or neonatal or "neo-natal" or postnatal or "post-natal") adj1 (outcome$ or complication$ or consequence$ or characteristic$ or event$ or result$ or problem$ or morbidit$ or sequelae)).ti,ab.

6. ((antepartum or "ante-partum" or intrapartum or "intra-partum" or peripartum or "peri-partum" or postpartum or "post-partum") adj1 (outcome$ or complication$ or consequence$ or characteristic$ or event$ or result$ or problem$ or morbidit$ or sequelae)).ti,ab.

7. Premature Birth/ or exp Fetal Membranes, Premature Rupture/ or Obstetric Labor, Premature/ or Infant, Extremely Premature/ or Infant, Premature/

8. (prematurity or "gestational age at birth" or "gestational age at delivery" or PTB or PTBs or VPTB or VPTBs or "pre-terms" or preterms or PTL or PTLs or VPTL or VPTLs or PTD or PTDs or VPTD or VPTDs or PROM or PPROM).ti,ab.

9. (("pre-term" or preterm or premature) adj2 (labour$ or labor$ or infant or deliver$ or birth$)).ti,ab.

10. ((preterm or "pre-term" or premature) adj1 rupture adj3 membrane$).ti,ab.

11. Fetal Growth Retardation/ or Infant, Low Birth Weight/ or Infant, Very Low Birth Weight/ or Infant, Extremely Low Birth Weight/ or Infant, Small for Gestational Age/

12. ((intrauterine or "intra-uterine" or fetal or foetal) adj1 growth adj1 (restrict$ or retardation)).ti,ab.

13. (SGA or SFGA or IUGR or FGR or "small for gestational age" or "small-for-gestational-age" or "small-for-gestational age" or "small for gestation" or "small-for-gestation").ti,ab.

14. (VSGA or "very-small-for-gestational-age" or "very-small-for-gestational age" or SFD or "small for dates" or "small-for-dates" or "weight for dates" or "weight for gestational age" or "weight for age at delivery" or "weight at delivery").ti,ab.

15. ("birthweight for dates" or "birthweight for gestational age" or "birthweight for age at delivery" or "birth weight for dates" or "birth weight for gestational age" or "birth weight for age at delivery" or "birth-weight for dates" or "birth-weight for gestational age" or "birth-weight for age at delivery").ti,ab.

16. (LBW or "low BW" or "low birth weight" or "low birth-weight" or "low-birth weight" or "low-birth-weight" or "low birthweight" or "low-birthweight" or "lower BW" or "lower birth weight" or "lower birth-weight" or "lower-birth weight" or "lower-birth-weight" or "lower birthweight" or "lower-birthweight").ti,ab.

17. ("reduced birth weight" or "reduced birthweight" or "reduced birth-weight" or VLBW or "very-low birthweight" or "very-low birth weight" or "very-low birth-weight" or "very-low-birthweight" or "very-low-birth-weight" or ELBW or "extremely-low birthweight" or "extremely-low birth weight" or "extremely-low birth-weight" or "extremely-low-birthweight" or "extremely-low-birth-weight").ti,ab.

18. Stillbirth/ or Fetal Death/

19. (stillbirth$ or "still birth$" or stillborn$ or "still born$" or abortion$ or miscarriage$).ti,ab.

20. ((pregnancy or gestational or fetal or foetal or obstetric$ or labor or labour or birth) adj1 (death$ or loss$ or demise$ mortalit$)).ti,ab.

21. ((delivery or neonate or newborn or "new-born" or "new born" or infant or reproductive or prelabour or

prelabor or "pre-labour" or "pre-labor") adj1 (death$ or loss$ or demise$ mortalit$)).ti,ab.

22. ((intrauterine or "intra-uterine" or antenatal or ante-natal or prenatal or "pre-natal" or perinatal or "peri-natal" or neonatal or "neo-natal" or postnatal or "post-natal" or antepartum or "ante-partum" or intrapartum or "intra-partum" or peripartum or "peri-partum" or postpartum or "post-partum") adj1 (death$ or loss$ or demise$ mortalit$)).ti,ab.

23. 1 or 2 or 3 or 4 or 5 or 6 or 7 or 8 or 9 or 10 or 11 or 12 or 13 or 14 or 15 or 16 or 17 or 18 or 19 or 20 or 21 or 22

24. HIV/ or HIV Seropositivity/ or HIV Infections/ or HIV-2/ or HIV-1/ or AIDS Serodiagnosis/ or Acquired Immunodeficiency Syndrome/ or AIDS Arteritis, Central Nervous System/ or AIDS-Associated Nephropathy/ or AIDS Dementia Complex/ or AIDS-Related Opportunistic Infections/ or Lymphoma, AIDS-Related/

25. (HIV or "HIV/AIDS" or PLHIV or PLWHA or WLHIV or WLWHA or "HIV-1" or "HIV-type-1" or "HTLV III" or "HTLV-III" or "HTLV type III" or "HTLV-type-III" or LAV or "HTLV-III-LAV" or "LAV-HTLV-III" or "HIV-2" or "HIV-type-2" or "HIV-II" or "HTLV-IV" or "LAV-2").ti,ab.

26. ("HIV-positive" or "HIV-1-positive" or "HIV-2-positive" or "HIV-infected" or "HIV-1-infected" or "HIV-type-1-infected" or "HTLV III-infected" or "HTLV-III-infected" or "HTLV type III-infected" or "HTLV-type-III-infected" or "LAV-infected").ti,ab.

27. ("HTLV-III-LAV-infected" or "LAV-HTLV-III-infected" or "HIV-2-infected" or "HIV-type-2-infected" or "HIV-II-infected" or "HTLV-IV-infected" or "LAV-2-infected" or "HIV-infection$" or "HIV-1-infection$" or "HIV-type-1-infection$" or "HTLV III-infection$" or "HTLV-III-infection$" or "HTLV type III-infection$" or "HTLV-type-III-infection$").ti,ab.

28. ("LAV-infection$" or "HTLV-III-LAV-infection$" or "LAV-HTLV-III-infection$" or "HIV-2-infection$" or "HIV-type-2-infection$" or "HIV-II-infection$" or "HTLV-IV-infection$" or "LAV-2-infection$" or "Human Immunodeficiency Virus$" or "Human Immune Deficiency Virus$").ti,ab.

29. ("Human T Cell Lymphotropic Virus Type III" or "Human T-Cell Lymphotropic Virus Type III" or "Human T Lymphotropic Virus Type III" or "Human T-Lymphotropic Virus Type III" or "Human T Lymphotropic Virus Type IV" or "Human T-Lymphotropic Virus Type IV" or "Human T Cell Leukemia Virus Type III" or "Human T-Cell Leukemia Virus Type III").ti,ab.

30. ("Lymphadenopathy-Associated Virus$" or "Lymphadenopathy Associated Virus$" or AIDS or "Acquired Immune Deficiency Syndrome" or "Acquired Immunodeficiency Syndrome").ti,ab.

31. 24 or 25 or 26 or 27 or 28 or 29 or 30

32. exp Anti-HIV Agents/ or exp HIV Fusion Inhibitors/ or exp HIV Integrase Inhibitors/ or exp HIV Protease Inhibitors/ or HIV Reverse Transcriptase/ or Reverse Transcriptase Inhibitors/

33. ((antiretroviral or "anti-retroviral" or antiviral$ or "anti-viral$" or "anti-HIV" or "anti-HIV-1") adj1 (treatment$ or therap$ or regimen$ or drug$ or agent$)).ti,ab.

34. (("anti-HIV-2" or "anti-AIDS") adj1 (treatment$ or therap$ or regimen$ or drug$ or agent$)).ti,ab.

35. (HAART or "HAART-exposed" or "HAART-treated" or "Mega-HAART" or ARV or ARVs or cARV or cARVs or "ARV-exposed" or "ARV-treated" or "combination-ARV" or "combination-ARVs" or "combined-ARV" or "combined-ARVs").ti,ab.

36. (ART or "Multi-ART" or "Triple-ART" or cART or "ART-exposed" or "ART-treated" or "combination-ART" or "combined-ART" or "sc-ART" or "short-course-antiretroviral therap$" or "short-course-anti-retroviral therap$").ti,ab.

37. (combin$ adj (treatment$ or therap$ or regimen$ or drug$ agent$)).ti,ab.

38. (monotherap$ or "mono-therap$" or "dual therap$" or "dual drug therap$" or bitherap$).ti,ab.

39. (PI or PIs or "PI-based" or "boosted-PI" or "PI-containing" or "PI-therap$" or "PI-treatment$" or "PI-regimen$" or "Ritonavir-boosted" or "protease inhibitor$" or NRTI or NRTIs or "NRTI-based" or "NRTI-containing" or "NRTI-therap$" or "NRTI-treatment$" or "NRTI-regimen$" or "nucleoside reverse transcriptase inhibitor$" or "nucleoside analog reverse transcriptase inhibitor$").ti,ab.

40. (NNRTI or NNRTIs or "NNRTI-based" or "NNRTI-containing" or "NNRTI-therap$" or "NNRTI-treatment$" or "NNRTI-regimen$" or "non nucleoside reverse transcriptase inhibitor$" or "non-nucleoside reverse transcriptase inhibitor$" or "nonnucleoside reverse transcriptase inhibitor$" or "non nucleoside analog reverse transcriptase inhibitor$" or "non-nucleoside analog reverse transcriptase inhibitor$" or "nonnucleoside analog reverse transcriptase inhibitor$").ti,ab.

41. (NtRTI or NtRTIs or "NtRTI-based" or "NtRTI-containing" or "NtRTI-therap$" or "NtRTI-treatment$" or "NtRTI-regimen$" or "nucleotide reverse transcriptase inhibitor$" or "nucleotide analog reverse transcriptase inhibitor$" or "fusion inhibitor$" or "CCR5 receptor antagonist$" or "integrase inhibitor$" or "maturation inhibitor$" or "entry inhibitor$").ti,ab.

42. Didanosine/ or Delavirdine/ or Emtricitabine/ or Lamivudine/ or Nevirapine/ or Rilpivirine/ or Stavudine/ or Tenofovir/ or Zidovudine/ or Atazanavir Sulfate/ or Darunavir/ or Ritonavir/ or Lopinavir/ or Nelfinavir/ or Saquinavir/ or Enfuvirtide/ or Maraviroc/ or Raltegravir Potassium/ or Indinavir/ or Zalcitabine/ or Cobicistat/

43. "Efavirenz, Emtricitabine, Tenofovir Disoproxil Fumarate Drug Combination"/ or "Emtricitabine, Rilpivirine, Tenofovir Drug Combination"/ or "Emtricitabine, Tenofovir Disoproxil Fumarate Drug Combination"/ or "Elvitegravir, Cobicistat, Emtricitabine, Tenofovir Disoproxil Fumarate Drug Combination"/

44. (Abacavir or ABC or Didanosine or ddI or Emtricitabine or FTC or Lamivudine or 3TC or Stavudine or d4T or Tenofovir or TFV or TDF or TAF or Zidovudine or AZT or ZDV or Delavirdine or DLV or Efavirenz or EFV or Etravirine or ETR or Nevirapine or NVP or Rilpivirine or RPV or Atazanavir or ATV or "Atazanavir/Ritonavir" or "ATV/r" or Darunavir or DRV or "Darunavir/Ritonavir" or "DRV/r" or Fosamprenavir or FPV or "Fosamprenavir/Ritonavir" or "FPV/r" or temsavir or TMR).ti,ab.

45. (Indinavir or IDV or "Indinavir/Ritonavir" or "IDV/r" or Lopinavir or LPV or "Lopinavir/Ritonavir" or "LPV/r" or Nelfinavir or NFV or "Nelfinavir/Ritonavir" or "NFV/r" or Ritonavir or RTV or Saquinavir or SQV or "Saquinavir/Ritonavir" or "SQV/r" or Tipranavir or TPV or "Tipranavir/Ritonavir" or "TPV/r" or Enfuvirtide or "T-20" or Maraviroc or MVC or Raltegravir or RAL or Elvitegravir or EVG or Zalcitabine or ddC or Combivir or Trizivir or Kaletra or Epzicom or Kivexa or Truvada or Atripla).ti,ab.

46. ("Integrase strand transfer inhibitor" or Dolutegravir or DTG or Tivicay or Isentress or Vitekta or "Formyl peptide receptor 1" or Fuzeon or FPR1 or ENF or Seizentry or Celsentri or Ziagen or Videx or Emtriva or Coviracil or Zerit or Viread or Vemlidy or Retrovir or Azidothymidine or "Diarylpyrimidine analogue" or Rescriptor or Sustiva or Intelence or TMC125 or DAPY or Viramune or Edurant or TMC278).ti,ab.

47. (Reyataz or APV or Agenerase or Prezista or Lexiva or Telzir or Crixivan or ABT-378 or Norvir or Viracept or AG1343 or Invirase or Fortovase or Aptivus or "Rilpivirine plus dolutegravir" or "RPV/DTG" or "Raltegravir plus lamivudine" or "RAL/3TC" or "Abacavir plus lamivudine plus dolutegravir" or "ABC/3TC/DTG" or "Emtricitabine plus tenofovir alafenamide" or "FTC/TAF" or "Emtricitabine plus rilpivirine plus tenofovir alafenamide" or "FTC/RPV/TAF" or "Atazanavir plus cobicistat" or "ATV/COBI" or "Darunavir plus cobicistat" or "DRV/COBI" or Juluca or Dutrebis or Stribild or Triumeq or Odefsey or Complera or Descovy or Genvoya or Evotaz or Prezcobix).ti,ab.

48. (Cobicistat or "cobicistat-boosted" or COBI or Rezolsta or QUAD or Epivir or Temixys or Cimduo or Selzentry or Doravirine or DOR or Pifeltro or "Ibalizumab-uiyk" or Hu5A8 or IBA or Ibalizumab or "TMB-355" or "TNX-355" or Trogarzo or Bictegravir or BIC or "Bictegravir, Emtricitabine, Tenofovir Alafenamide" or "bictegravir sodium/emtricitabine/tenofovir alafenamide fumarate" or "BIC/FTC/TAF" or Biktarvy or "tenofovir alafenamide fumarate" or "darunavir ethanolate, cobicistat, emtricitabine, tenofovir alafenamide fumarate" or "DRV/COBI/FTC/TAF").ti,ab.

49. (Symtuza or "Dolutegravir and lamivudine" or "dolutegravir sodium/lamivudine" or "DTG/3TC" or Dovato or "Doravirine, lamivudine, and tenofovir disoproxil fumarate" or "doravirine/lamivudine/tenofovir disoproxil fumarate" or "DOR/3TC/TDF" or Delstrigo or "Efavirenz, lamivudine, and tenofovir disoproxil fumarate" or "EFV/3TC/TDF" or Symfi or "Symfi Lo" or "Elvitegravir, cobicistat, emtricitabine, and tenofovir alafenamide fumarate" or "elvitegravir/cobicistat/emtricitabine/tenofovir alafenamide fumarate" or "EVG/COBI/FTC/TAF" or Genvoya or "TMC-114" or "TMC114" or Dideoxyinosine or Racivir or Heptovir or Hepitec).ti,ab.

50. (Zerut or Estavudina or Sanilvudine or Apropovir or Stocrin or Zrivada or Aluvia or Aluviran or Pentafuside or Symtuza or "Efavirenz, Lamivudine, Tenofovir Disoproxil Fumarate drug combination" or "Lopinavir-Ritonavir drug combination").ti,ab.

51. (NRTTI or NRTTIs or "NRTTI-based" or "NRTTI-containing" or "NRTTI-therap$" or "NRTTI-treatment$" or "NRTTI-regimen$" or "nucleoside reverse transcriptase translocation inhibitor" or islatravir or ISL or "MK-8591" or "portmanteau inhibitor$" or "capsid inhibitor$" or "CAI" or lenacapavir or "LEN" or "GS-CA1" or leronlimab or albuvirtide or Eviplera or "rilpivirine, emtricitabine and tenofovir disoproxil fumarate" or "attachment inhibitor" or "gp120 attachment inhibitor" or Rukobia or fostemsavir or "FTV" or INSTI or INSTIs or Vocabria or cabotegravir or "CAB" or cabenuva or Tybost or "INSTI-based" or "INSTI-containing" or "INSTI-therap$" or "INSTI-treatment$" or "INSTI-regimen$").ti,ab.

52. 32 or 33 or 34 or 35 or 36 or 37 or 38 or 39 or 40 or 41 or 42 or 43 or 44 or 45 or 46 or 47 or 48 or 49 or 50 or 51

53. 31 or 52
54. 23 and 53

Appendix 2

Quality assessment of studies

Appendix 2.1. **Adapted Newcastle-Ottawa quality assessment tool**
A study can be awarded a maximum of one point (for items indicated with an asterisk) for each numbered criterion within the "Selection" and "Outcome" categories.

Selection (maximum 4 points)

1) Representativeness of the exposed cohort.
   - a) Truly representative of the pregnant population in the community. *
   - b) Somewhat representative of the pregnant population in the community.
   - c) Selected group of users, e.g. nurses, volunteers, teenage mothers.
   - d) No description of the derivation of the cohort.

2) Selection of the comparator cohort.
   - a) The comparator cohort is drawn from the same community as the exposed cohort. *
   - b) The comparator cohort is drawn from a different source than the exposed cohort.
   - c) No description of the derivation of the comparator cohort.

3) Ascertainment of exposure.
   - a) ART intake monitored as part of study. *
   - b) ART intake confirmed from secure medical records (e.g. hospital records). *
   - c) Structured interview-participant reported ART intake.
   - d) Written self-report.
   - e) No description.

4) Demonstration that outcome of interest was not present at start of study.
   - a) Yes. *
   - b) No.

Comparability (maximum 2 points)

1) Comparability of cohorts on the basis of the analysis. In the analysis:
   - a) Study controls for BMI, smoking, parity, and maternal age. *
   - b) Study controls for one or more additional factors: e.g. prior history of adverse pregnancy outcome, maternal hypertension, anaemia, illicit drug or alcohol use in pregnancy. *
   - c) Confounding factors not controlled for.

Outcome (maximum 3 points)

1) Ascertainment of outcome.
   - a) Outcome was confirmed following clinical observation of outcome by clinician, midwife or trained birth attendant. *
   - b) Medical records. *
   - c) Self-report.
   - d) No description.

2) Method used to assess gestational age.
   - a) Gestational age was determined according to early ultrasound ($<$14 weeks). *
   - b) Gestational age was determined by: late ultrasound ($\geq$14 weeks' gestation) or last normal menstrual period or neonatal assessment, e.g. Ballard score, or a combination of these methods.
   - c) No description.

3) Follow up of cohorts

a) Complete follow up - all subjects accounted for. *

b) Subjects lost to follow up unlikely to introduce bias, i.e. < 20 % lost to follow up. *

c) Follow up rate < 80% (lost to follow-up > 20%).

d) No description.

Appendix 2.2

Classification of studies according to quality assessment

Good Quality	9 points – all requirements met
Average Quality	3 points in “Selection” and 3 points in “Outcome” sections
	≥2 points in the “Selection” and “Outcome” sections, as well as ≥1 point in the “Comparability” section.
Poor Quality	< 2 points in the “Selection” and/or “Outcome” sections.
	2 points in the “Selection” and “Outcome” sections, but no points in the “Comparability” section.

Appendix 2.3

Quality assessment of studies included in the systematic review and meta-analysis

Study	Representativeness of the exposed cohort	Selection of comparator cohort	Ascertainment of exposure	Demonstration that outcome of interest was not present at start of study	Comparability of cohorts on the basis of analysis	Ascertainment of outcome	Method used to assess gestational age	Follow up of cohorts	Total quality assessment
**Adam (2016)** [25]	Truly representative of the average pregnant female population in the community.*	Same community as exposed pop*	ART intake monitored as part of study*	No	Study controls for one or more additional factor*	Clinical observation*	Unspecified	Complete follow up*	Average
**Bailey (2013)** [26]	Truly representative*	Same community as exposed pop*	Medical records*	No	Confounding factors not controlled for	Medical records*	LNMP and ultrasound (unspecified)	<20% lost to follow up*	Poor
**Bengtson (2020)** [27]	Truly representative of the average pregnant female population in the community.*	Same community as exposed pop*	ART intake monitored as part of study*	Yes*	Confounding factors not controlled for	Clinical observation*	Ultrasound (unspecified), LMP or fundal height	Complete follow up*	Poor
**Chen (2012)**[28]	Truly representative*	Same community as exposed cohort*	Medical records*	No	Study controls for one or more additional confounding factors*	Medical records*	LNMP, SFH or ultrasound (unspecified)	<20% lost to follow up*	Average
**Dadabhai (2019)** [29]	Somewhat representative (CD4 >=350)	Same community as exposed pop*	Medical records*	Yes*	Controls for: maternal age, parity, BMI (not smoking)* Study controls for one or more additional confounding factors*	Clinical observation*	Ballard score and LMP	Complete follow up*	Average
**Djeha (2019)** [30]	Truly representative of the pregnant population*	Same community as exposed pop*	Medical records*	Yes*	Confounding factors not controlled for	Medical records*	First trimester ultrasound or LMP*	Complete follow up *	Average
**Ekouevi (2008)**[31]	Truly representative*	Same community as exposed pop*	ART intake monitored as part of study*	Yes*	Study controls for one or more additional confounding factors*	Clinical observation*	Unspecified	<20% lost to follow up*	Average
**Goetghebuer (2019)** [32]	Truly representative of the pregnant population*	The comparator cohort is drawn from the same community as the exposed population*	Medical records*	Yes*	Study controls for one or more additional confounding factors*	Medical records*	Ballard score	Complete follow up *	Average

**Hu (2019)** [33]	Truly representative of the average pregnant female population in the community.*	Same community as exposed pop*	Medical records*	Yes"	Study controls for one or more additional confounding factors*	Clinical observation*	First or second trimester ultrasound, if unable to assess ultrasound LMP used	Complete follow up*	Average
**Kowalska (2003)** [34]	No description	Same community as exposed pop*	ART intake monitored as part of study*	Yes*	Study controls for one or more additional confounding factors*	No description	Unspecified	Complete follow up*	Poor
**Li (2016)** [35]	truly representative*	Same community as exposed pop*	ART intake monitored as part of study*	Yes*	Study controls for one or more additional confounding factors*	No description	LNMP and SFH	Complete follow up*	Poor
**Li (2020)** [36]	Truly representative of the average pregnant female population in the community.*	Same community as exposed pop*	ART intake monitored as part of study*	Yes"	Study controls for one or more additional confounding factors*	Clinical observation*	LMP or ultrasound (unspecified)	<20% lost to follow up*	Average
**Malaba (2017)** [37]	Truly representative*	Same community as exposed pop*	Self-reported	Yes*	Study controls for one or more additional confounding factors*	Medical records*	LNMP and SFH	<20% lost to follow up*	Average
**Malaba (2021)** [38]	Truly representative*	Same community as exposed pop*	Medical records*	Yes*	Study controls for one or more additional confounding factors*	Medical records*	LMP, SFH and/or ultrasound (unspecified)	<20% lost to follow up*	Average
**Marazzi (2011** [39]	Somewhat representative	Same community as exposed pop*	Medical records*	No	Controls for CD4 count, viral load, initiation and duration of ART received.* additional factors: anaemia. *	Medical records*	LNMP and clinical exam (unspecified)	<20% lost to follow up*	Average
**Moodley (2016)** [40]	Truly representative*	Same community as exposed pop*	Medical records*	No	Study controls for one or more additional confounding factors*	Medical records*	LNMP and/or ultrasound (unspecified)	<20% lost to follow up*	Average
**Olagbuji (2010)** [41]	Somewhat representative (tertiary hospitals, and patients with AIDS and/or chronic medical disorders predating pregnancy excluded from study)	Same community as exposed pop*	No description	Yes*	Controls for: maternal age (no parity, BMI, smoking). Controls for additional factor: marriage*	Unspecified	Unspecified	No statement	Poor

**Ramokolo (2017)** [42]	Truly representative of the average pregnant female population in the community.*	Same community as exposed pop*	Self-reported	No	Study controls for one or more additional confounding factors*	Medical records*	LNMP	Complete follow up, all subjects accounted for*	Average
**Rempis (2017)** [43]	Somewat representative	Same community as exposed pop*	Written self-reported	No	Study controls for one or more additional confounding factors*	Medical records*	Unspecified	Subjects lost to follow up- unlikely to introduce bias, small number lost <20 % follow-up.*	Poor
**Rubin (2011)** [44]	Truly representative*	Same community as exposed pop*	ART intake monitored as part of study*	Yes*	Confounding factors not controlled for	No description	Unspecified	No statement	Poor
**Santosa (2019)** [45]	Truly representative of the pregnant population in the community*	Same community as the exposed population*	Medical records*	Yes*	Confounding factors corrected for **	Clinical observation*	Ultrasound <14 weeks*	Complete follow up*	Good
**Saums (2019)** [46]	Truly representative of the pregnant population* (each cohort representative of the average pregnant population from which they are drawn)	The comparator cohort is drawn from the same community as the exposed population*	Medical records*	No	Study controls for one or more additional confounding factors*	Medical records*	Unspecified	Complete follow up*	Average
**Sebitloane (2017)** [47]	Truly representative of the average pregnant female population in the community.*	Same community as exposed pop*	Medical records*	No	Confounding factors not controlled for	Medical records*	Unspecified	Complete follow up*	Poor
**Short (2014)** [48]	Truly representative*	Same community as exposed cohort*	Medical records*	No	Confounding factors not controlled for	Medical records*	Unspecified	Complete follow up*	Poor
**Silverman (2010)** [49]	No description	Different source population	No description	No	Study controls for one or more additional confounding factors*	No description	Unspecified	<20% lost to follow up*	Poor
**Snijdewind (2018)** [50]	Truly representative*	Same community as exposed pop*	Medical records*	No	Study controls for one or more additional confounding factors*	Medical records*	LNMP and/or ultrasound (unspecified)	Complete follow up*	Average

**Tan (2023)** [51]	Truly representative of the pregnant population*	The comparator cohort is drawn from the same community as the exposed population*	Medical records*	Yes*	Confounding factors not controlled for	Structured interviews with nurses and medical records*	Unspecified	<20% lost*	Average
**Tiam (2019)** [52]	Truly representative of the pregnant population in the community*	Same community as the exposed population*	Medical records*	Yes*	Study controls for one or more additional confounding factors*	Clinical observation*	LMP	<20% lost to follow up*	Poor
**Yu (2012)** [53]	Truly representative*	Same community as exposed pop*	ART intake monitored as part of study*	No	Study controls for one or more additional confounding factors*	No description	Unspecified	Complete follow up*	Poor
**Zash (2017)** [54]	Truly representative*	Same community as exposed cohort*	Medical records*	No	Study controls for one or more additional confounding factors*	Medical records*	LNMP and/or ultrasound (unspecified) or fundal height	Complete follow up*	Average
**Zash (2018)** [55]	Truly representative*	Same community as exposed cohort*	Medical records*	No	Study controls for one or more additional confounding factors*	Clinical observation*	LNMP	<20% lost to follow up*	Average

Abbreviations: ART= antiretroviral therapy, BMI= body mass index, LNMP= last normal menstrual period.

Appendix 2.4

Confounding factors adjusted for in included studies

Study	Methods to correct for confounding factors	**Regression analysis** Confounders corrected for:	**Risk factor analysis** Risk factors not significantly different between groups:	Matching
**Adam (2016)** [25]	Risk factor analysis	-	Age, urban residence, anaemia	-
**Bailey (2013)** [26]	None	None (regression analysis assesses risk of perinatal transmission and receipt of zidovudine monotherapy, not perinatal outcome)	No P-value reported for demographics	-
**Bengtson (2020)** [27]	None	-	No p values reported	-
**Chen (2012)**[28]	Regression analysis, risk factor analysis	Covariates with p<=0.05 and CD4 count in pregnancy, (<=200 cells, >200 cells, unknown)<br>SGA: multivariate analysis also included advanced maternal age, nulliparity, maternal hypertension in pregnancy and anaemia (anaemia excluded from analyses that included preconception HAART women).<br>PTB: maternal hypertension, anaemia.	Nationality, education, parity, antenatal care received, syphilis, alcohol, smoking, CD4 count,	No matching
**Dadabhai (2019)** [29]	Regression analysis	Maternal age, gravidity, previous pregnancy losses and adverse outcomes, maternal education (yrs of schooling), BMI at enrolment, anaemia, electricity	-	-
**Djeha (2019)** [30]	None	-	No P-value reported for demographics	-
**Ekouevi (2008)**[31]	Regression analysis	Adjusted for age, ART regime, CD4 count, WHO stage and BMI	Age, living with partner, income, education level	-
**Goetghebuer (2019)** [32]	Risk factor analysis	-	Education, literacy, occupation, smoking, alcohol, GBS positive swab	-
**Hu (2019)** [33]	Regression, risk factor analysis	Mothers' education, ethnicity, household registration, parity, gestational age at first AN care, number of AN care visits, mode of delivery	Mother's age, marital status	No matching
**Kowalska (2003)** [34]	Risk factor analysis	None	Maternal age, CD4 count, viral load, and hard drug use	No matching

**Li (2016)** [35]	Risk factor analysis	None	Age, year of delivery, diarrhoea, AIDS-defining illness, tuberculosis history,	No matching
**Li (2020)** [36]	Regression, risk factor analysis	Maternal age, residence, ethnicity, education level, occupation, gravidity, parity, severe anaemia, pregnant syphilis, HBV infection	Maternal age, urban/non-urban, ethnicity, parity, syphilis, HBV infection	-
**Malaba (2017)** [37]	Regression, risk factor analysis	Age, maternal height, parity, previous PTD, CD4 count and VL	Socio-economic status, height, CD4 count	No matching
**Malaba (2021)** [38]	Regression analysis	Maternal substance use, smoking	-	Infant age, sex and geocode
**Marazzi (2011**[39]	Regression analysis	Mantel-Haenszel adjustment = CD4 count. Multivariate analysis adjusted for baseline viral load, baseline CD4 cell count, baseline haemoglobin, baseline BMI, predelivery days in care, gestational age at the beginning of HAART	Demographics not stratified according to treatment groups	-
**Moodley (2016)** [40]	Regression analysis, risk factor analysis	HIV- vs HIV +, multivariable adjusted for: year age group, mode of delivery, HIV status<br>ART regiment comparison, multivariable adjusted for: year age group, mode of delivery, HIV status, CD4, ART regimen	Age, year, mode of delivery, CD4 count	No matching
**Olagbuji (2010)** [41]	Risk factor analysis	None	Maternal age (mean and age distribution), marital status	HIV positive women were 'appropriately matched' to HIV negative women but no details of criteria given - hence not counted as matched.
**Ramokolo (2017)** [42]	Risk factor analysis	Information not provided	Syphilis serology, tuberculosis, maternal age, parity, household food insecurity, infant gender, maternal education,	No matching
**Rempis (2017)** [43]	Risk factor analysis	None	Age, no of persons in household, income, socio-economic status, travel distance to hospital, referral, grand multiparity, hypertension, MIP reported, anaemia, ANC attendance, no of ANC visits	No matching
**Rubin (2011)** [44]	None	None	No demographic characteristics reported	No matching
**Santosa (2019)** [45]	Regression, risk factor analysis	Corrected for maternal age, smoking, alcohol consumption, pre-pregnancy BMI, parity and history of adverse perinatal outcomes	Between HIV positive and HIV negative: Married/cohabiting, occupation, smoked during pregnancy, alcohol during pregnancy, pre-pregnancy BMI, history of prior adverse perinatal outcomes, gestational age at enrolment	-

**Saums (2019)** [46]	Risk factor analysis	-	Parity, History of diabetes, hypertension, Diabetes, Obesity. IDU	-
**Sebitloane (2017)** [47]	None	None	None	No matching
**Short (2014)** [48]	None	None	None	No matching
**Silverman (2010)** [49]	Risk factor analysis	None	CD4 count, anaemia, multiple birth, febrile illness, hypertension, diabetes, malaria	No matching
**Snijdewind (2018)** [50]	Regression, risk factor analysis	SGA (preconception v antenatal initiation) cART regimen, region of origin, parity LBW+VLBW (preconception v antenatal): Smoking, IDU, parity, gestational age at birth PTB: (preconception v antenatal initiation): cART regimen, maternal age, CD4+ count at delivery, trough CD4+ cell count, region of origin, smoking, IDU VPTB (preconception vs antenatal initiation): type of drug regimen, maternal age at birth, region of origin, smoking, illicit drug use, CD4+ cell count and delivery mode	(HIV- vs HIV+) Regions of origin, smoking, alcohol, IDU, Mode of delivery,	No
**Tan (2023)** [51]	Regression analysis	Maternal age, gravidity, HBV or HCV co-infection, the HIV transmission mode, the timing of HIV diagnosis, CD4 count at delivery, smoking and alcohol intake, and year of pregnancy	not conducted	No matching
**Tiam (2019)** [52]	None	-	-	-
**Yu (2012)** [53]	Risk factor analysis	Multivariate logistic regression performed does not include ART treatment in the model.	Age, nationality, weight, gravidity, HIV transmission route, parity, mode of delivery, viral load, cotrimoxazole, haemoglobin, CD4 count,	No matching
**Zash (2017)** [54]	Regression analysis	Maternal age, gravidity, education	-	-
**Zash (2018)** [55]	Regression analysis	Maternal age, gravida, and educational attainment	-	-

Abbreviations: ALT = alanine transaminase, ART= antiretroviral therapy, ARV = anti-retroviral drugs, BMI= body mass index, CDC= Centers for Disease Control and Prevention, GBS= group B streptococcus, HAART= highly active antiretroviral therapy, HBV= hepatitis B virus, HCV= hepatitis C virus, HDL = high density lipoprotein, HIV= human immunodeficiency virus, IDU= illicit drug use, IVDU = intravenous drug use, NNRTI = non-nucleoside reverse transcriptase inhibitor , LDL = low density lipoprotein, PTB=preterm birth, PTD = preterm delivery, SGA = small for gestational age, STD= sexually transmitted disease.

Appendix 3

Random-effects meta-analyses of risk of perinatal outcomes associated with timing of initiation of ART for women living with HIV.

Forest plots showing random-effects meta-analyses of risk of perinatal outcomes associated with WLHIV receiving preconception ART vs HIV-negative women, WLHIV receiving antenatal ART vs HIV-negative women, WLHIV receiving preconception ART vs WLHIV naïve to ART, and WLHIV receiving antenatal ART vs WLHIV naïve to ART. Risk ratios (RRs) and 95% confidence intervals (CIs) of individual studies and summary estimates are shown. All forest plots are based on unadjusted outcome frequencies of perinatal outcomes.

Appendix 3.1

Women living with HIV receiving preconception ART vs HIV-negative women

Figure 3.1.1 Preterm birth in women living with HIV receiving preconception ART vs HIV-negative women

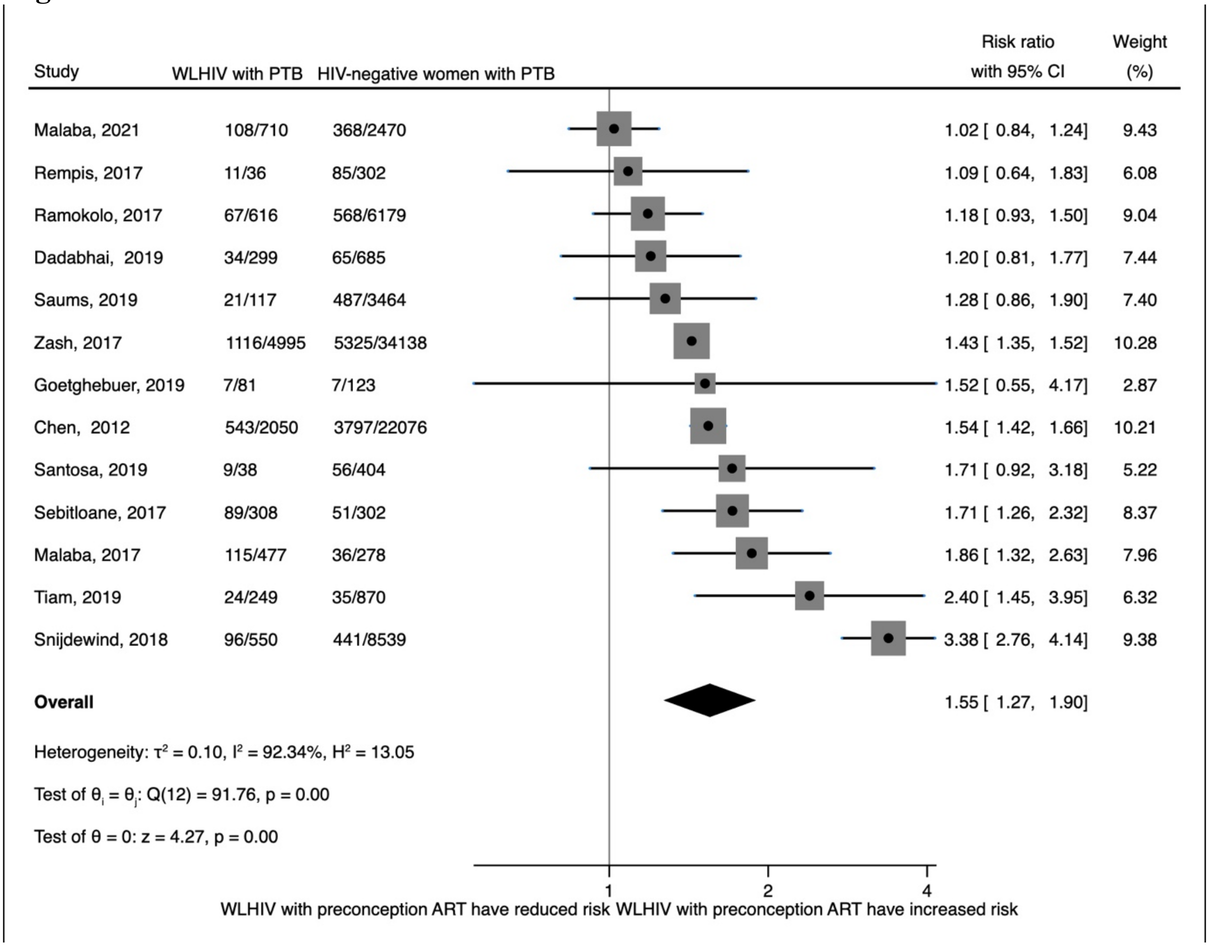

Peters' test for small study effects: p-value= 0.3448

Figure 3.1.2 Very preterm birth in women living with HIV receiving preconception ART vs HIV- negative women

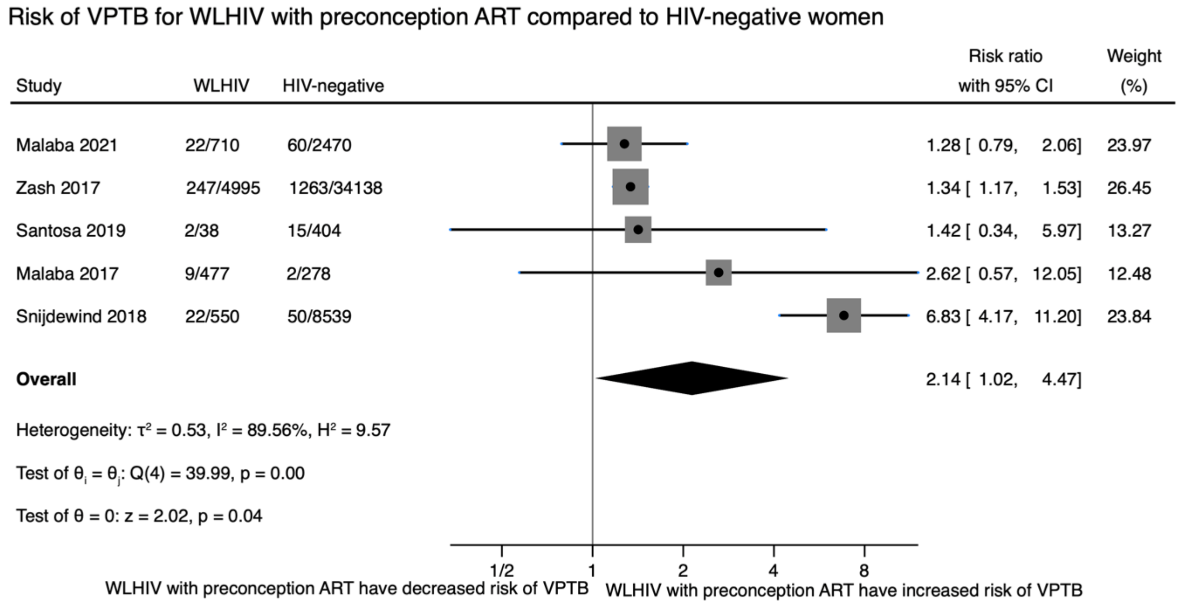

Figure 3.1.3 Low birthweight in women living with HIV receiving preconception ART vs HIV- negative women

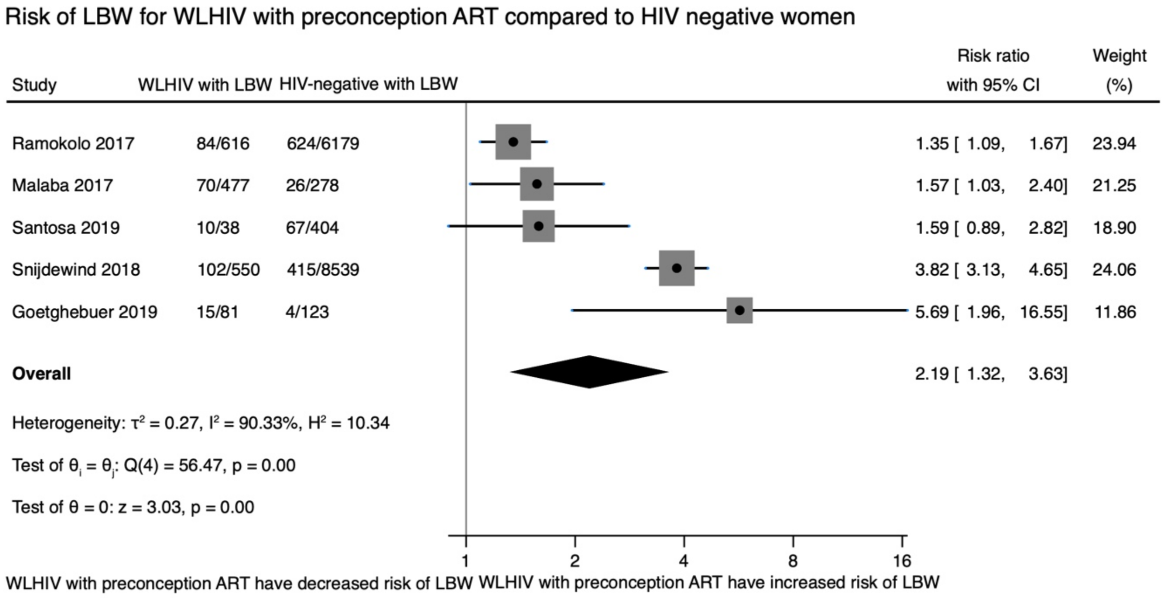

Figure 3.1.4 Very low birthweight in women living with HIV receiving preconception ART vs HIV- negative women

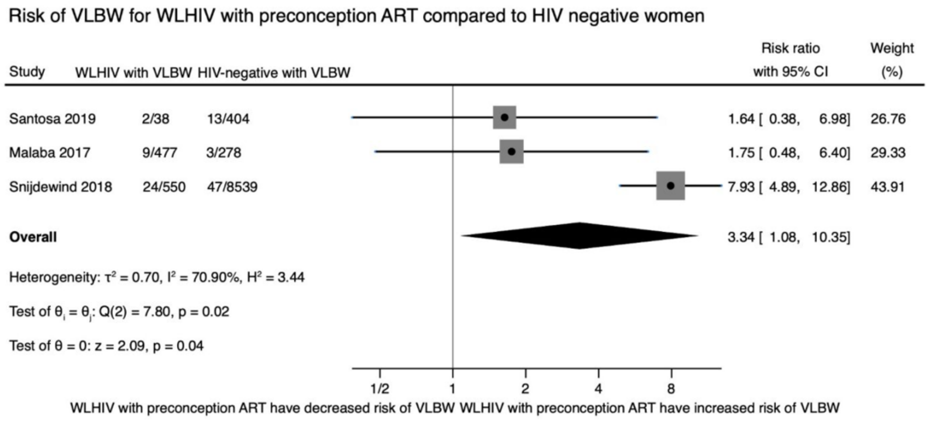

Figure 3.1.5 Small for gestational age in women living with HIV receiving preconception ART vs HIV- negative women

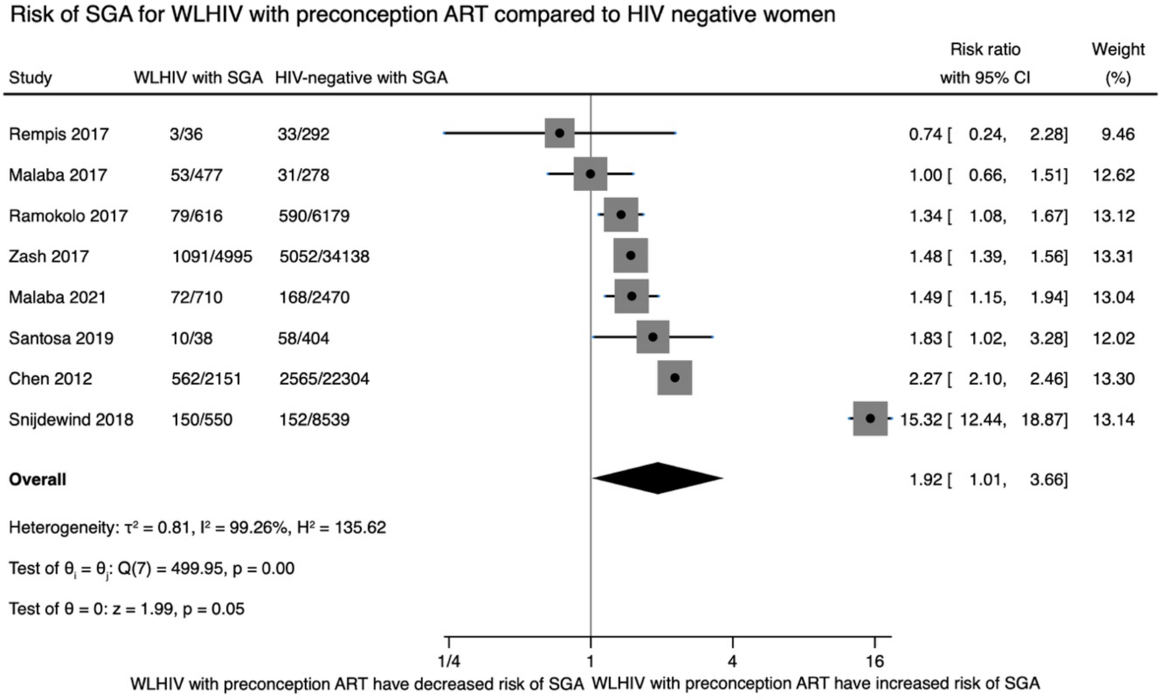

Figure 3.1.6 Very small for gestational age in women living with HIV receiving preconception ART vs HIV- negative women

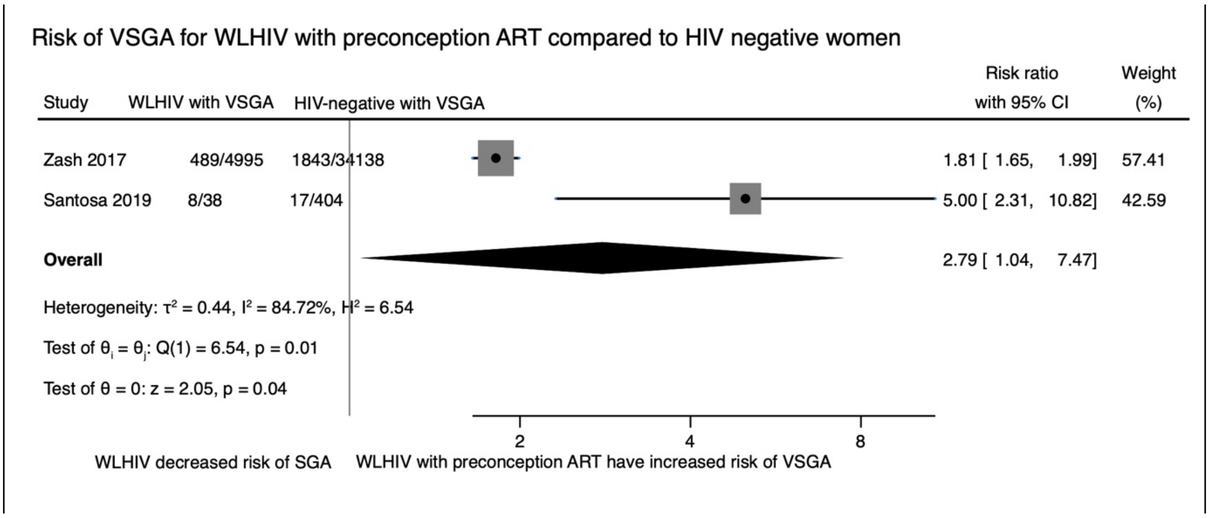

Figure 3.1.7 Neonatal death in women living with HIV receiving preconception ART vs HIV-negative women

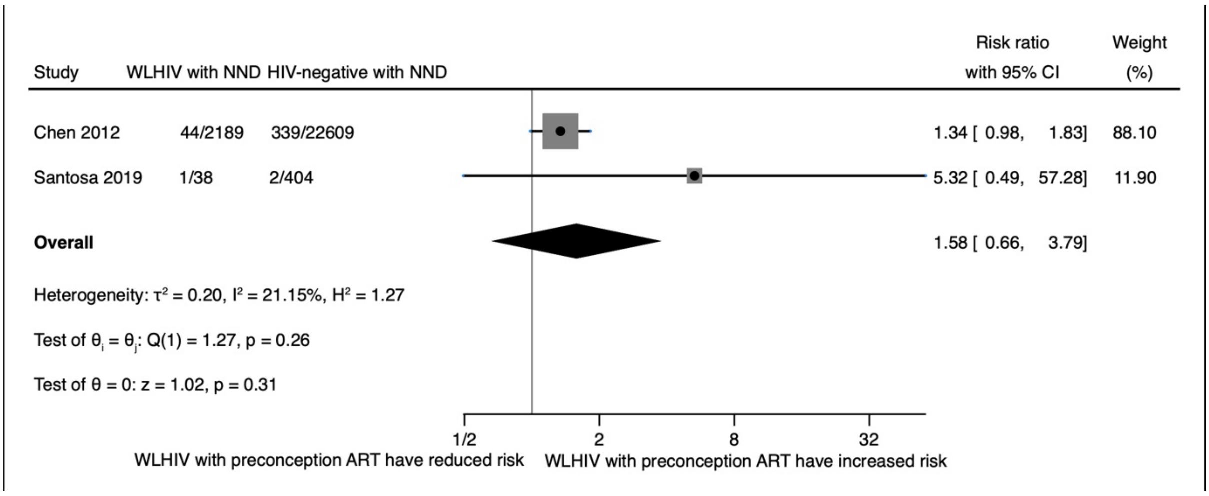

Figure 3.1.8 Spontaneous Preterm Birth and Stillbirth in women living with HIV receiving preconception ART vs HIV-negative women

Outcome	Study	RR	95% CI	P-value	WLHIV preconception	HIV-negative
sPTB	Malaba 2021	0.92	0.69, 122	P=0.55	54/710	205/2470
SB	Santosa 2019	0.42	0.03, 6.88	P=0.54	0/38	12/404

Appendix 3.2

Women living with HIV receiving antenatal ART vs HIV-negative women

Figure 3.2.1 Preterm birth in women living with HIV receiving antenatal ART vs HIV-negative women

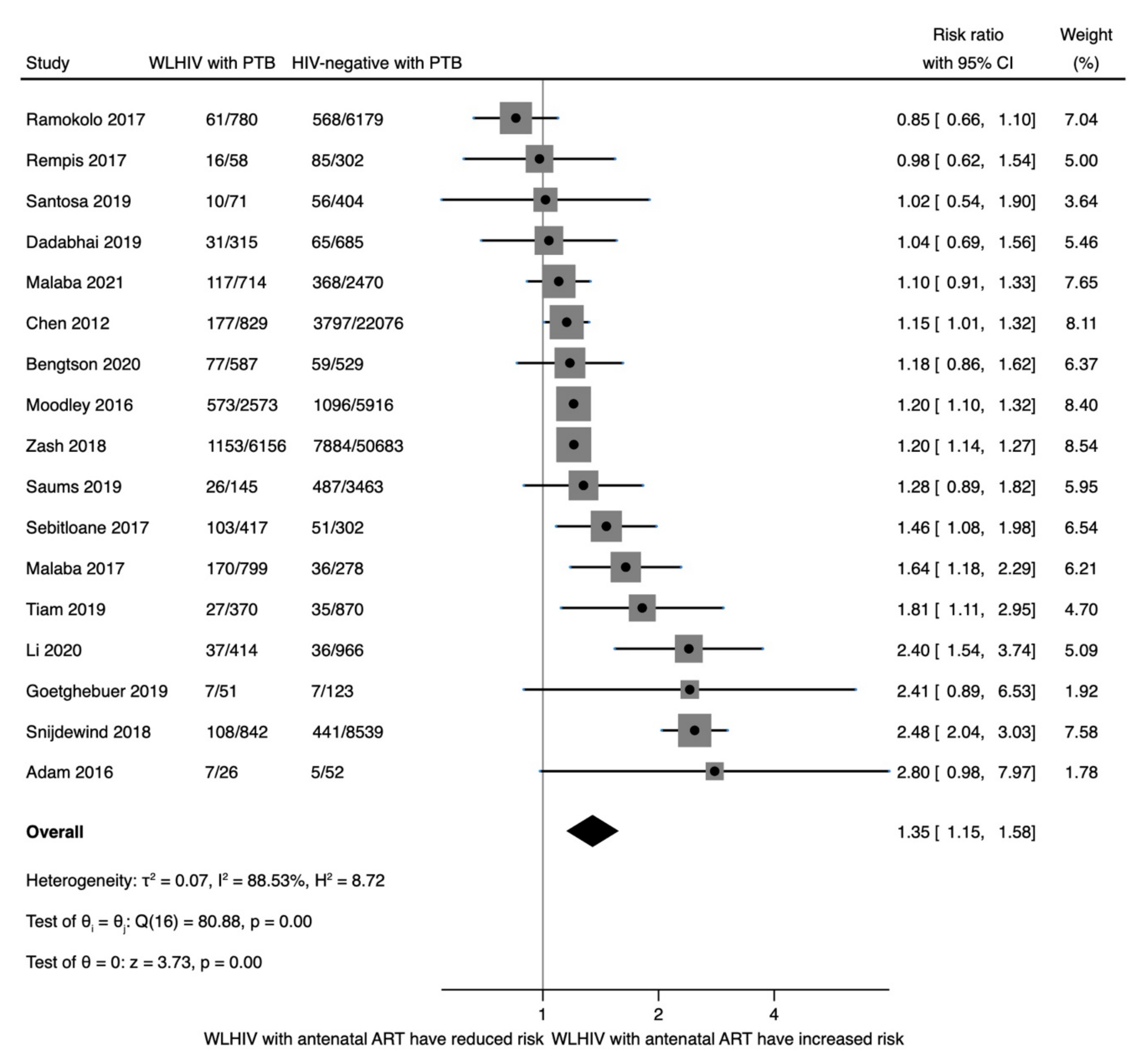

Peters' test for small study effects: p-value= 0.001

Figure 3.2.2 Very preterm birth in women living with HIV receiving antenatal ART vs HIV-negative women

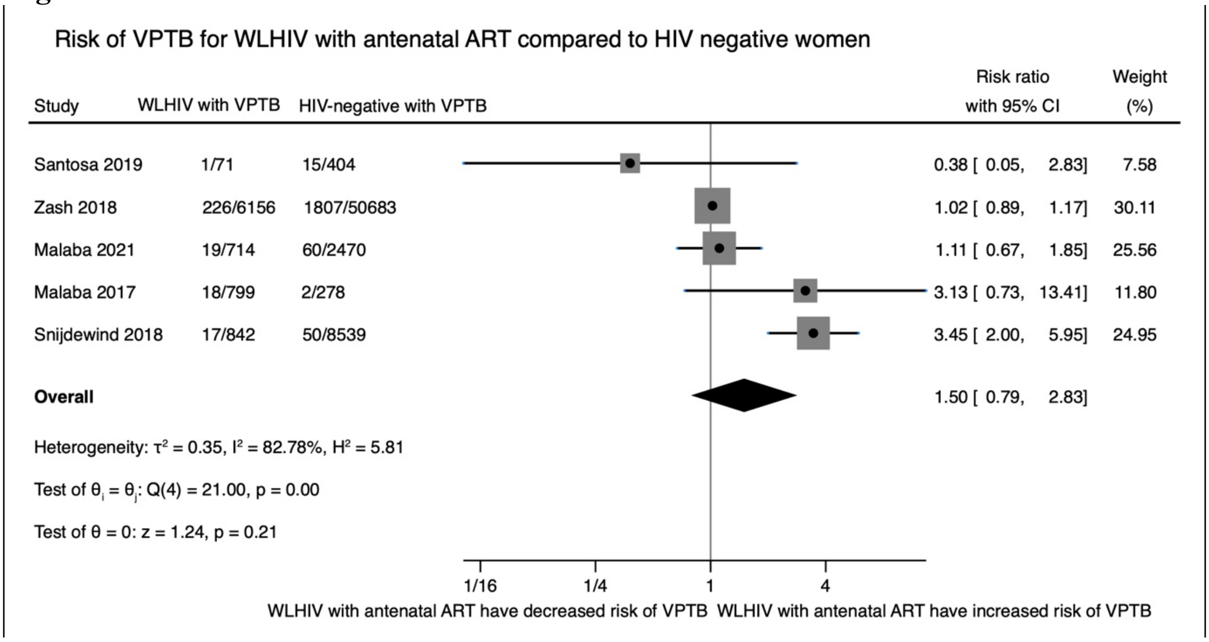

Figure 3.2.3 Low birthweight in women living with HIV receiving antenatal ART vs HIV-negative women

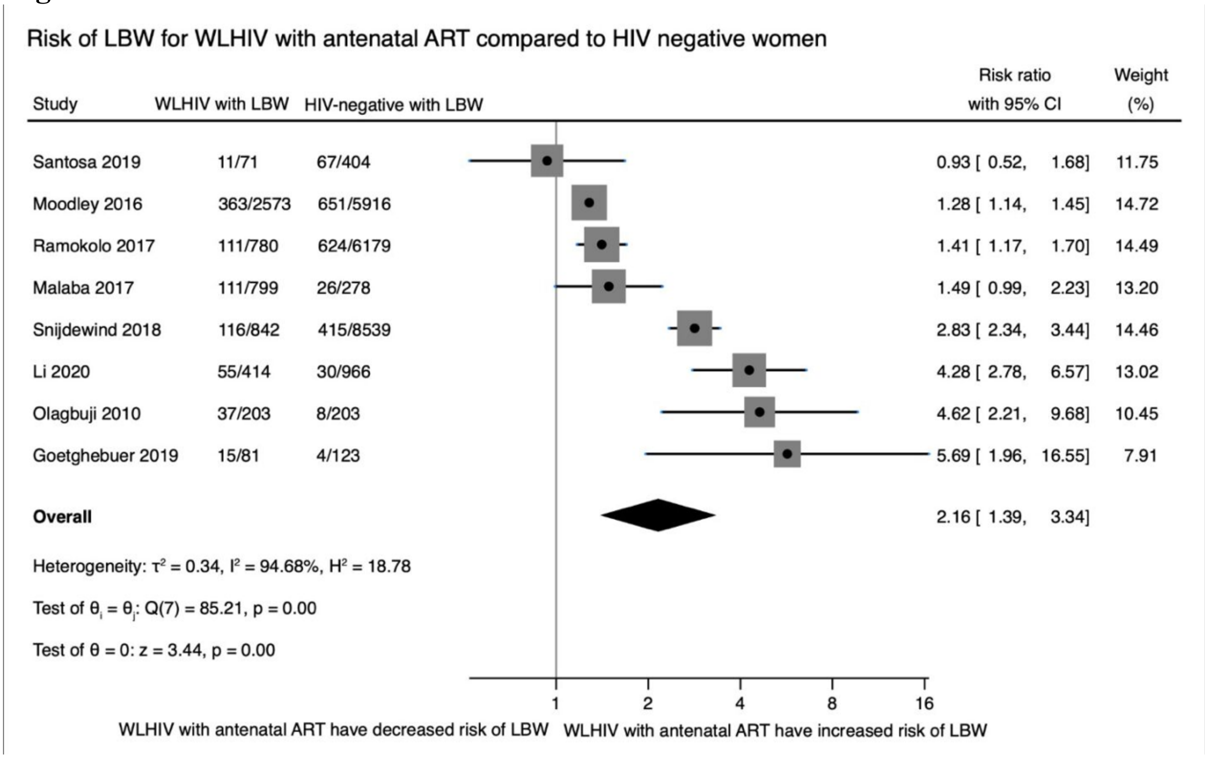

Figure 3.2.4 Very low birthweight in women living with HIV receiving antenatal ART vs HIV-negative women

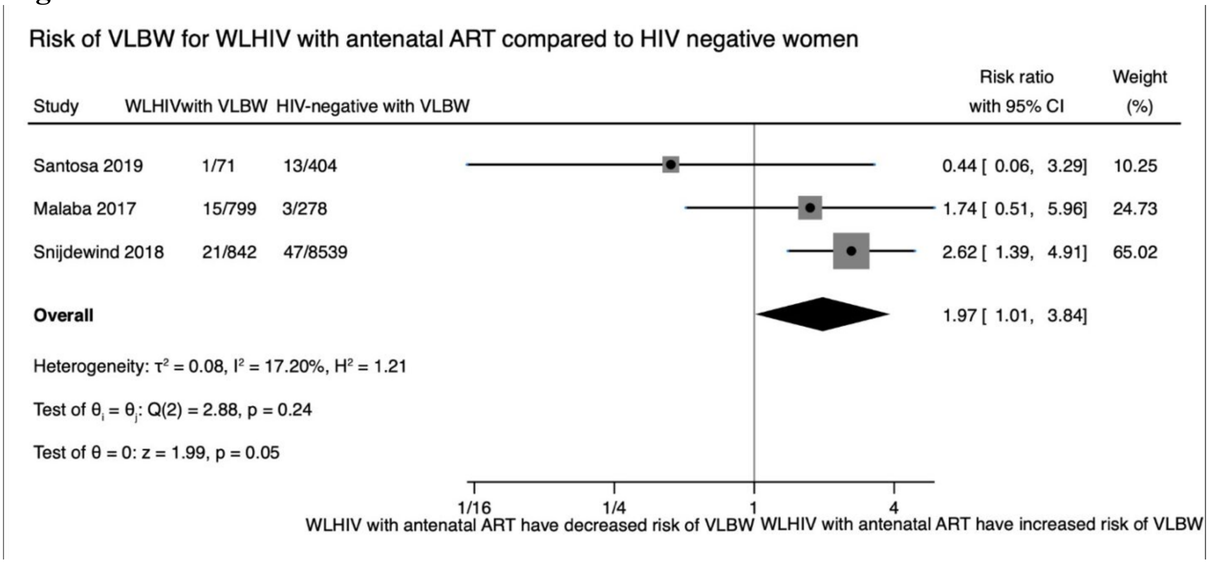

Figure 3.*2*.5 Small for gestational age in women living with HIV receiving antenatal ART vs HIV- negative women

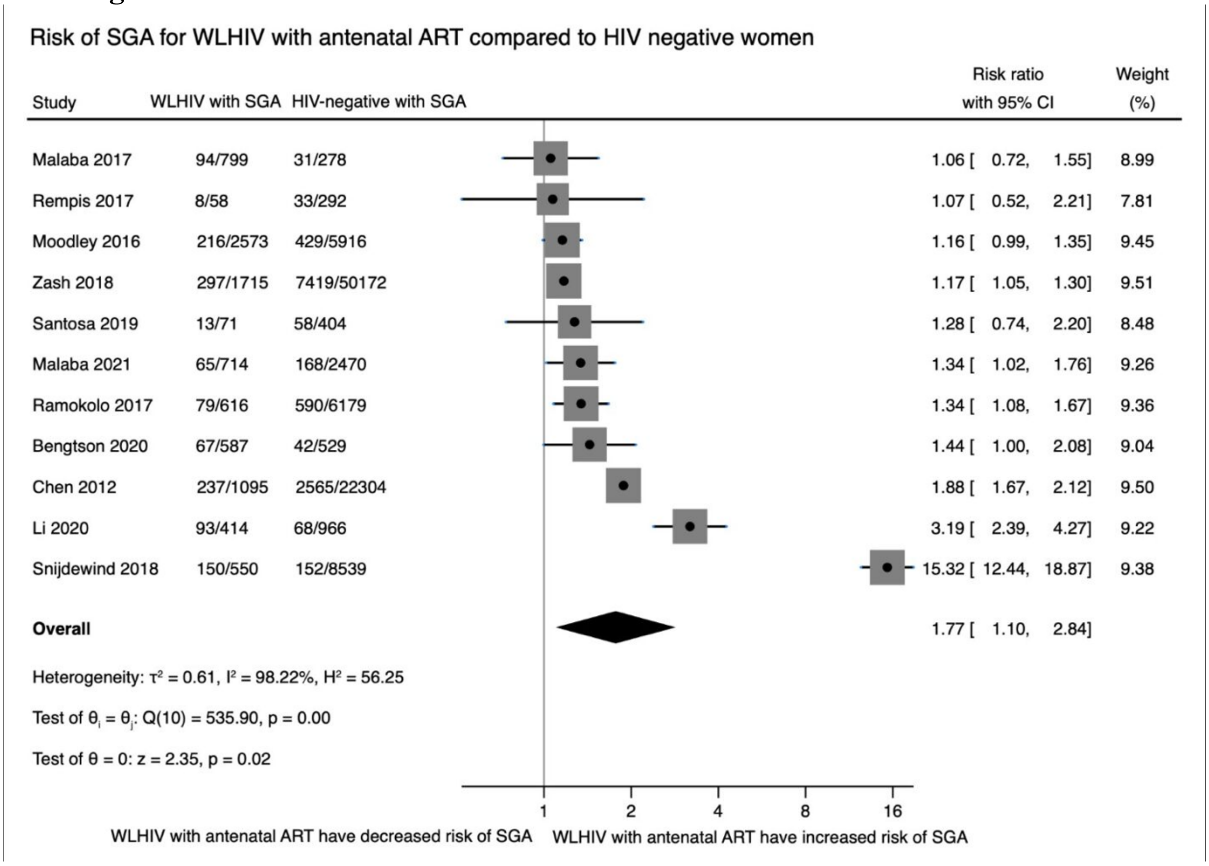

Peters' test for small study effects: p-value= 0.0101

Figure 3.2.6 Very small for gestational age in women living with HIV receiving antenatal ART vs HIV- negative women

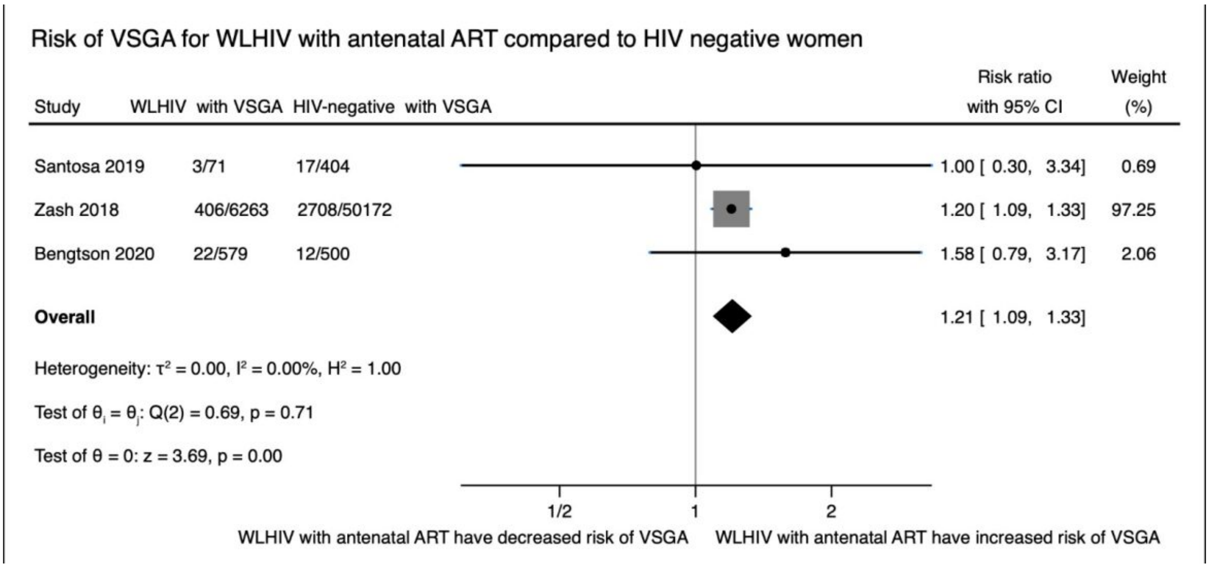

Figure 3.2.7 Neonatal death in women living with HIV receiving antenatal ART vs HIV-negative women

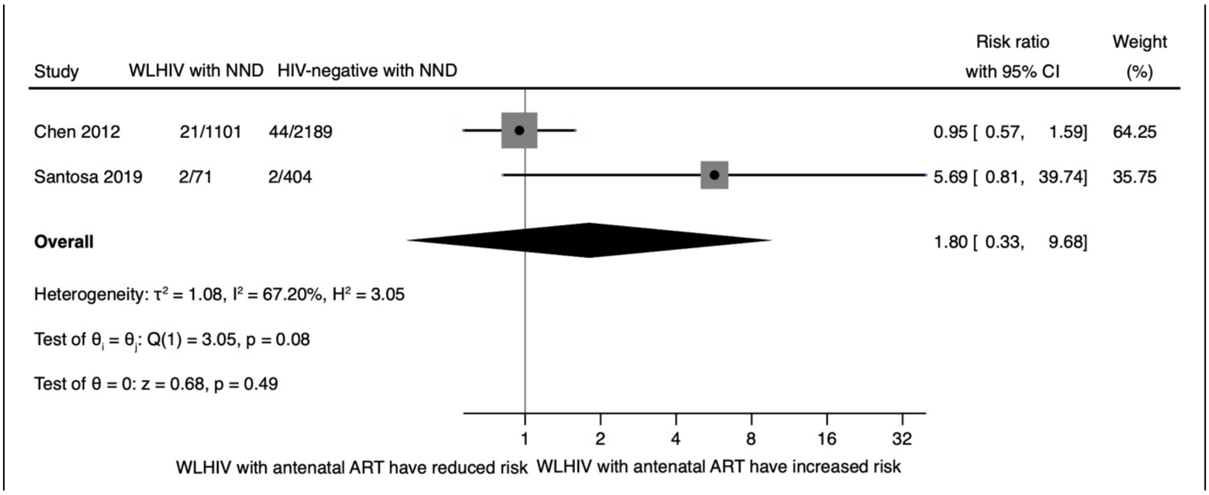

Figure 3.2.8 Spontaneous Preterm Birth and stillbirth in women living with HIV receiving antenatal ART vs HIV-negative women

Outcome	Study	RR	95% CI	P-value	WLHIV with antenatal ART	HIV-negative
sPTB	Malaba 2021	1.08	0.83, 1.41	P=0.57	64/714	205/2470§
SB	Santosa 2019	1.13	0.15, 8.45	P=0.91	12/404	1/38

Appendix 3.3

Women living with HIV receiving preconception ART vs women living with HIV naïve to ART

Figure 3.3.1 Preterm birth in women living with HIV receiving preconception ART vs women living with HIV naïve to ART

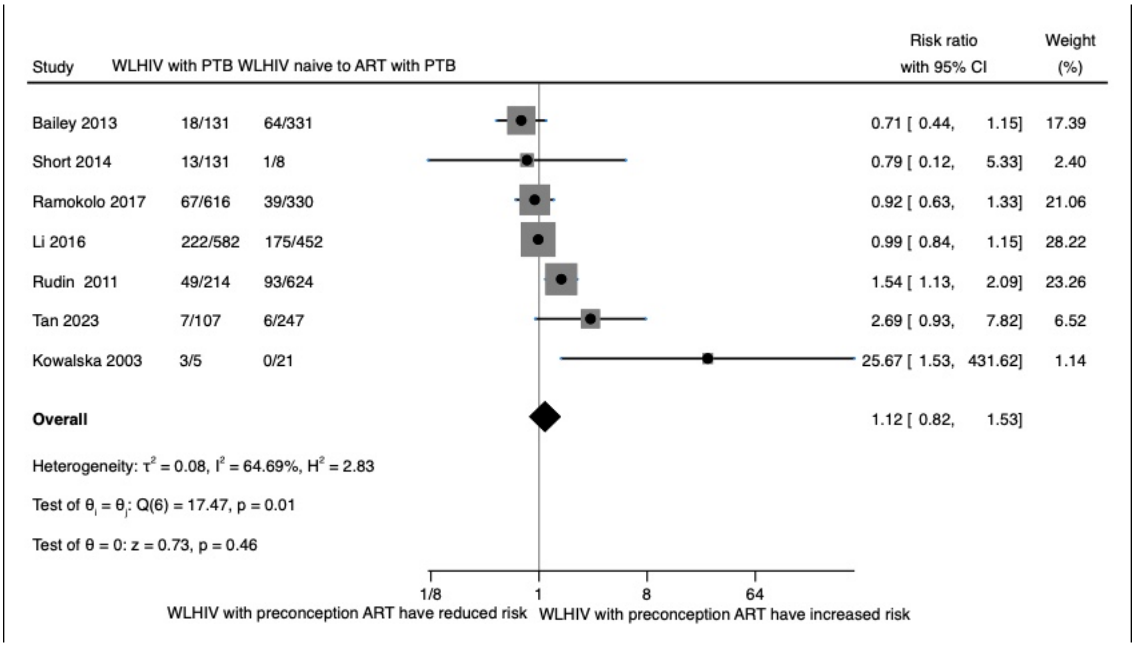

Figure 3.3.2 Low birth weight in women living with HIV receiving preconception ART vs women living with HIV naïve to ART

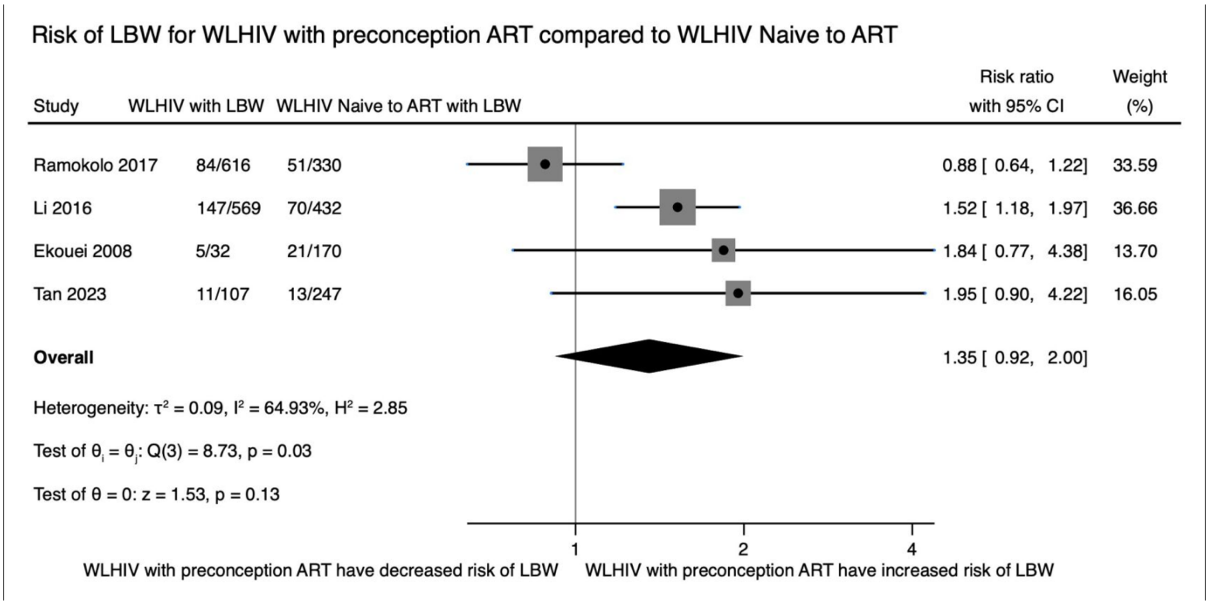

Figure 3.3.3 Small for gestational age in women living with HIV receiving preconception ART vs women living with HIV naïve to ART

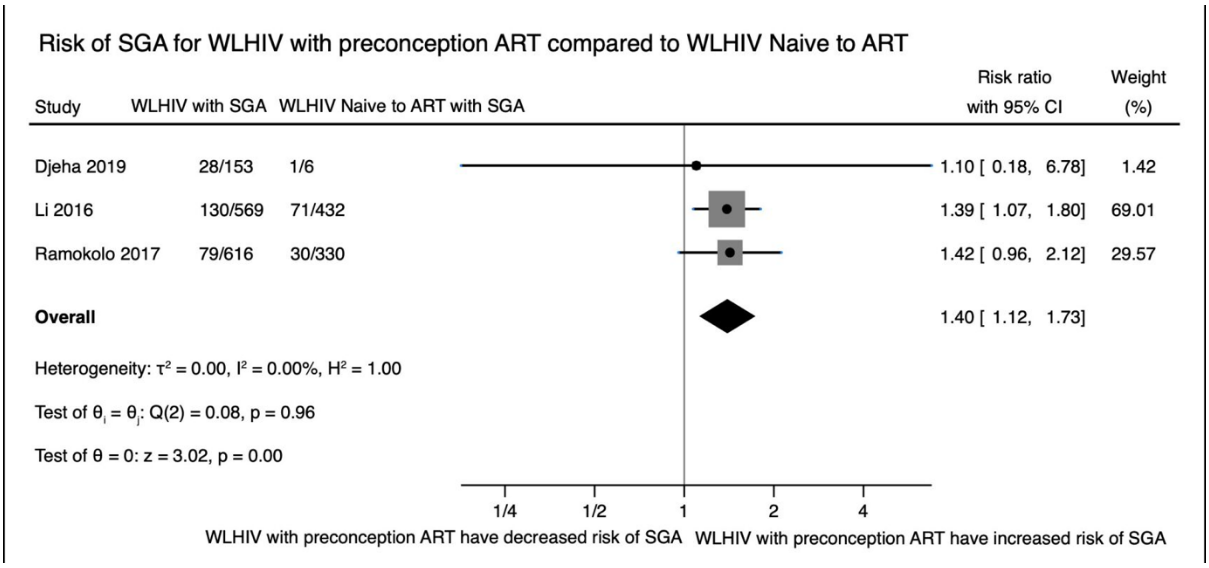

Figure 3.3.4 Very small for gestational age and very preterm birth in women living with HIV receiving preconception ART vs women living with HIV naïve to ART

Outcome	Study	RR	95% CI	P-value	WLHIV Preconception ART	WLHIV naive to ART
VSGA	Li 2016	2.44	1.64, 3.66	P<0.001	90/569	28/432

Appendix 3.4

Women living with HIV receiving antenatal ART vs women living with HIV naïve to ART

Figure 3.4.1 Preterm birth in women living with HIV receiving antenatal ART vs women living with HIV naïve to ART

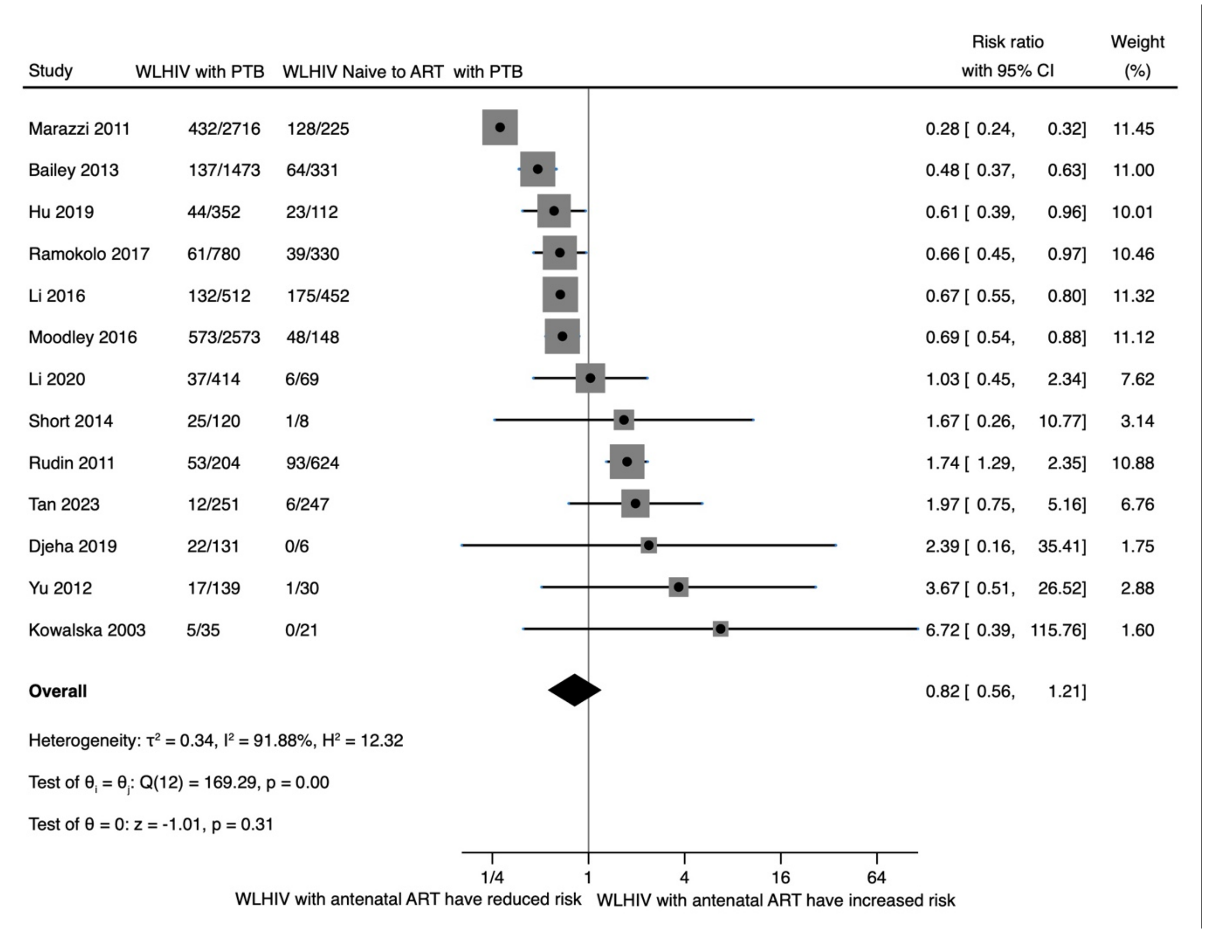

Peters' test for small study effects: p-value<0.001

Figure 3.4.3 Low birth weight in women living with HIV receiving antenatal ART vs women living with HIV naïve to ART

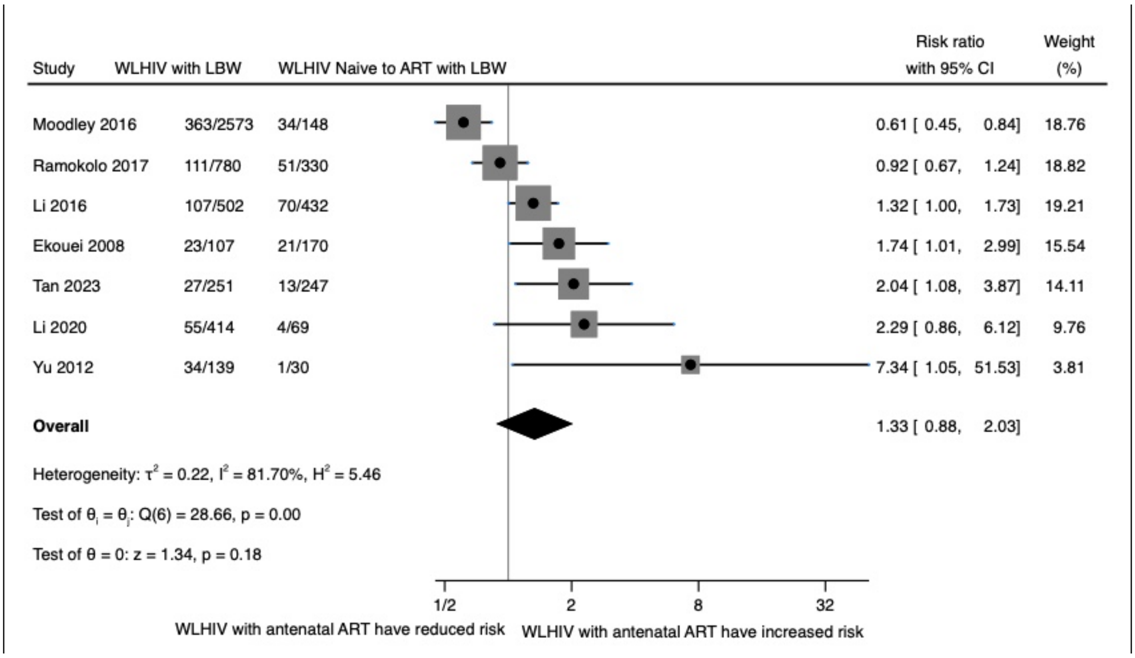

Figure 3.4.4 Small for gestational age in women living with HIV receiving antenatal ART vs women living with HIV naïve to ART

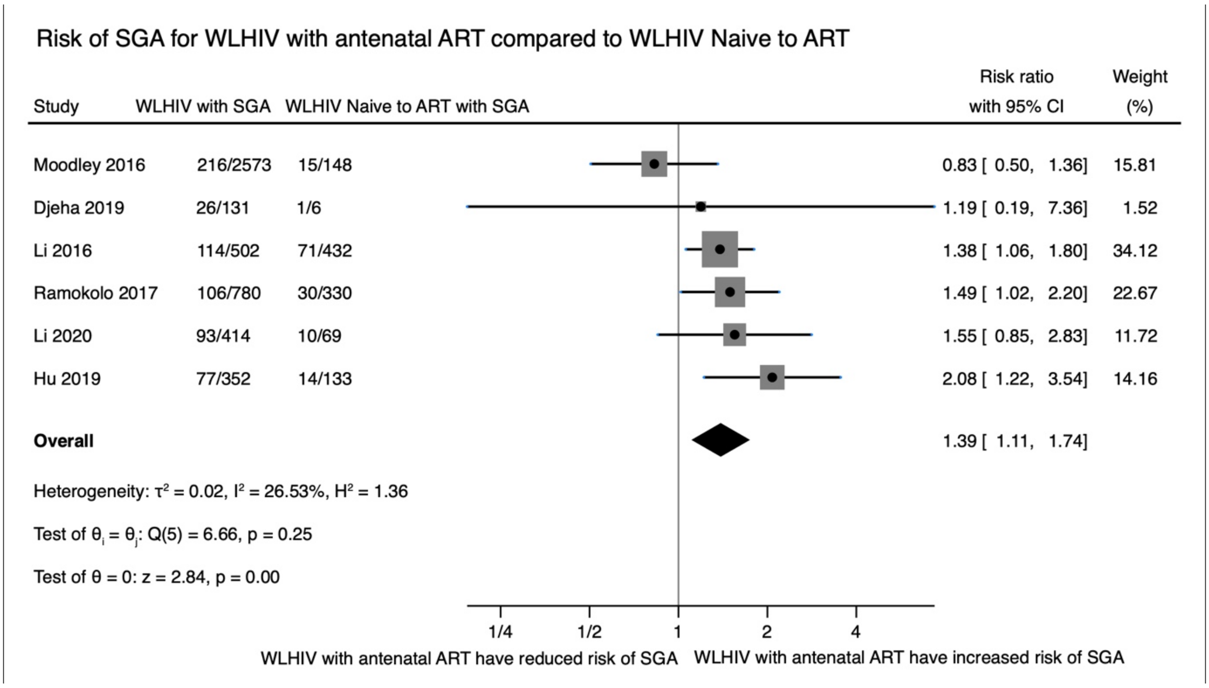

Figure 3.4.5 Very small for gestational age and stillbirth in women living with HIV receiving antenatal ART vs women living with HIV naïve to ART

Outcome	Study	RR	95% CI	P-value	WLHIV antenatal initiation	WLHIV naive to ART
VSGA	Li 2016	2.24	1.48, 3.40	P<0.001	73/502	28/432
SB	Tan 2023	1.23	0.33, 4.53	P=0.76	5/251	4/247

Appendix 4
Subgroup analyses
Appendix 4.1
Subgroup analysis by country income status: risk ratios

		Perinatal outcomes								
		**PTB**	**VPTB**	**sPTB**	**LBW**	**VLBW**	**SGA**	**VSGA**	**Stillbirth**	**NND**
		RR (95%CI)	RR (95%CI)	RR (95%CI)	RR (95%CI)	RR (95%CI)	RR (95%CI)	RR (95%CI)	RR (95%CI)	RR (95%CI)
**HIV- Negative Women**										
**WLHIV with preconception ART vs HIV-negative women**	**High income countries**	1.98 (1.00, 3.90)	6.83 (4.17, 11.20)	NA	3.87 (3.18, 4.70)	7.93 (4.89, 12.46)	15.23 (12.44, 18.87)	NA	NA	NA
	**Low- and Middle-Income Countries**	1.42 (1.23, 1.64)	1.34 (1.18, 1.52)	NA	1.41 (1.18, 1.69)	1.70 (0.65, 4.47)	1.51 (1.20, 1.89)	2.79 (1.04, 7.47)	NA	1.58 (0.66, 3.79)
**WLHIV with antenatal ART vs HIV-negative women**	**High income countries**	1.91 (1.16, 3.14)	3.45 (2.00, 5.95)	NA	3.27 (1.88, 5.69)	2.62 (1.39, 4.91)	15.32 (12.44, 18.87)	NA	NA	NA
	**Low- and Middle-Income Countries**	1.24 (1.10, 1.39)	1.03 (0.91, 1,18)	NA	1.86 (1.12, 3.07)	1.11 (0.31, 3.94)	1.43 (1.16, 1.77)	1.21 (1.09, 1.33)	NA	1.09 (0.76, 1.56)
**ART-naïve WLHIV**										
**WLHIV with preconception ART vs ART-naïve WLHIV**	**High income countries**	1.51 (1.11, 2.15)	NA	NA	NA	NA	1.10 (0.18, 6.78)	NA	NA	NA
	**Low- and Middle-Income Countries**	0.97 (0.85, 1.12)	NA	NA	1.35 (0.92, 2.00)	NA	1.40 (1.13, 1.74)	NA	NA	NA
**WLHIV with antenatal ART vs ART-naïve WLHIV**	**High income countries**	1.75 (1.30, 2.34)	NA	NA	NA	NA	1.19 (0.19, 7.36)	NA	NA	NA
	**Low- and Middle-Income Countries**	0.67 (0.56, 1.21)	NA	NA	1.33 (0.88, 2.03)	NA	1.39 (1.10, 1.79)	NA	NA	NA

A RR > 1 indicates increased risk of a perinatal outcome associated with WLHIV with preconception/antenatal ART initiation. For example, WLHIV with preconception ART initiation are associated with an increased risk of preterm birth compared to HIV negative women (RR 1.98 (1.00, 3.90)) in high income countries.

Abbreviations: ART= antiretroviral therapy, HIV= human immunodeficiency virus, LBW= low birthweight, NND= neonatal death, PTB= preterm birth, RR= risk ratio, SGA= small for gestational age, sPTB= spontaneous preterm birth, VLBW= very low birthweight, VPTB= very preterm birth, VSGA= very small for gestational age.

Appendix 4.2

Subgroup analysis by country income status: number of studies and women analysed

		Perinatal outcomes								
		**PTB**	**VPTB**	**sPTB**	**LBW**	**VLBW**	**SGA**	**VSGA**	**Stillbirth**	**NND**
		Number of studies (number of women analysed)	Number of studies (number of women analysed)	Number of studies (number of women analysed)	Number of studies (number of women analysed)	Number of studies (number of women analysed)	Number of studies (number of women analysed)	Number of studies (number of women analysed)	Number of studies (number of women analysed)	Number of studies (number of women analysed)
**HIV- Negative Women**										
**WLHIV with preconception ART vs HIV-negative women**	**High income countries**	3 (12,874)	1 (9,089)	NA	2 (9,293)	1 (9,089)	1 (9,089)	NA	NA	NA
	**Low- and Middle-Income Countries**	10 (77,482)	4 (43,510)	NA	3 (7,992)	2 (1,197)	7 (75,078)	2 (39,575)	NA	2 (24,432)
**WLHIV with antenatal ART vs HIV-negative women**	**High income countries**	3 (13,164)	1 (9,381)	NA	2 (9,585)	1 (9,381)	1 (9,089)	NA	NA	NA
	**Low- and Middle-Income Countries**	14 (105,881)	4 (61,575)	NA	6 (18,786)	2 (1,552)	10 (96,285)	3 (57,989)	NA	3 (25,949)
**ART-naïve WLHIV**										
**WLHIV with preconception ART vs ART-naïve WLHIV**	**High income countries**	2 (632)	NA	NA	NA	NA	1 (159)	NA	NA	NA
	**Low- and Middle-Income Countries**	6 (2,822)	NA	NA	4 (2503)	NA	2 (1,647)	NA	NA	NA
**WLHIV with antenatal ART vs**	**High income countries**	3 (956)	NA	NA	NA	NA	1 (137)	NA	NA	NA

**ART-naïve WLHIV**	**Low- and Middle-Income Countries**	10 (11,210)	NA	NA	7 (6,192)	NA	5 (5,733)	NA	NA	NA

Abbreviations: ART= antiretroviral therapy, HIV= human immunodeficiency virus, LBW= low birthweight, NND= neonatal death, PTB= preterm birth, SGA= small for gestational age, sPTB= spontaneous preterm birth, VLBW= very low birthweight, VPTB= very preterm birth, VSGA= very small for gestational age.

Appendix 4.3

Subgroup analysis by country income status: I^2 values

		Perinatal outcomes								
		**PTB**	**VPTB**	**sPTB**	**LBW**	**VLBW**	**SGA**	**VSGA**	**Stillbirth**	**NND**
		I^2 (95%CI) (p value)	I^2 (95%CI) (p value)	I^2 (95%CI) (p value)	I^2 (95%CI) (p value)	I^2 (95%CI) (p value)	I^2 (95%CI) (p value)	I^2 (95%CI) (p value)	I^2 (95%CI) (p value)	I^2 (95%CI) (p value)
**HIV- Negative Women**										
**WLHIV with preconception ART vs HIV-negative women**	**High income countries**	86.42% (p<0.001)	NA	NA	0.00% (p=0.47)	NA	NA	NA	NA	NA
	**Low- and Middle-Income Countries**	81.09% (p<0.001)	0.00% P=0.85	NA	0.00% (p=0.75)	0.00% (p=0.95)	91.56% (p<0.001)	84.72% P=0.01	NA	21.15% P=0.26
**WLHIV with antenatal ART vs HIV-negative women**	**High income countries**	77.48% (p=0.01)	NA	NA	37.13% (p=0.21)	NA	NA	NA	NA	NA
	**Low- and Middle-Income Countries**	74.25% P=0.01	0.00% P=0.35	NA	94.43% (p<0.001)	23.56% (P=0.25)	89.06% P<0.001	0.00% P=0.71	NA	0.00% P=0.22
**ART-naïve WLHIV**										
**WLHIV with preconception ART vs ART-naïve WLHIV**	**High income countries**	0.00% P=0.50	NA	NA	NA	NA	NA	NA	NA	NA
	**Low- and Middle-Income Countries**	0.00% P=0.03	NA	NA	64.95% p=0.03	NA	0.00% P=0.92	NA	NA	NA
**WLHIV with antenatal ART vs ART-naïve WLHIV**	**High income countries**	0.00% P=0.97	NA	NA	NA	NA	NA	NA	NA	NA
	**Low- and Middle-Income Countries**	89.50% P<0.001	NA	NA	81.70% P<0.001	NA	33.89% P=0.16	NA	NA	NA

Abbreviations: ART= antiretroviral therapy, HIV= human immunodeficiency virus, LBW= low birthweight, NND= neonatal death, PTB= preterm birth, SGA= small for gestational age, sPTB= spontaneous preterm birth, VLBW= very low birthweight, VPTB= very preterm birth, VSGA= very small for gestational age.

Appendix 4.4

Subgroup analysis by country income status: test of subgroup difference

Subgroup analyses by income status	Perinatal outcomes								
	**PTB**	**VPTB**	**sPTB**	**LBW**	**VLBW**	**SGA**	**VSGA**	**Stillbirth**	**NND**
	p-value	p-value	p-value	p-value	p-value	p-value	p-value	p-value	p-value
**WLHIV preconception ART vs HIV- women**									
**High income vs low/middle income**	P=0.35	P<0.001	NA	P<0.001	P=0.01	P<0.001	NA	NA	NA
**WLHIV antenatal ART vs HIV- women**									
**High income vs low/middle income**	P=0.09	P<0.001	NA	P=0.14	P=0.24	P<0.001	NA	NA	NA
**WLHIV Preconception ART vs WLHIV naïve to ART**									
**High income vs low/middle income**	P=0.01	NA	NA	NA	NA	P=0.79	P=0.96	NA	NA
**WLHIV Antenatal ART vs WLHIV naïve to ART**									
**High income vs low/middle income**	P<0.001	NA	NA	NA	NA	P=0.87	NA	NA	NA

Test of subgroup differences by country income using a random-effects DerSimonian-Laird model. Abbreviations: ART= antiretroviral therapy, LBW= low birthweight, NND= neonatal death, PTB= preterm birth, SGA= small for gestational age, sPTB= spontaneous preterm birth, VLBW= very low birthweight, VPTB= very preterm birth, VSGA= very small for gestational age.

Appendix 4.5

Subgroup analysis by study quality: risk ratios

		Perinatal outcomes								
		PTB	VPTB	sPTB	LBW	VLBW	SGA	VSGA	Stillbirth	NND
		RR (95%CI)	RR (95%CI)	RR (95%CI)	RR (95%CI)	RR (95%CI)	RR (95%CI)	RR (95%CI)	RR (95%CI)	RR (95%CI)
**HIV- Negative Women**										
**WLHIV with preconception ART vs HIV-negative women**	**Good Quality**	1.71 (0.92, 3.18)	1.42 (0.34, 5.97)	NA	1.59 (0.89, 2.82)	4.22 (0.98, 18.18)	1.83 (1.02, 3.28)	5.00 (2.31, 10.82)	NA	5.32 (0.49, 57.38)
	**Average Quality**	1.51 (1.17, 1.95)	2.29 (0.97, 5.39)	NA	2.39 (1.27, 4.50)	1.64 (0.38, 6.98)	2.17 (0.98, 4.81)	1.81 (1.65, 1.99)	NA	1.34 (0.98,1.83)
	**Poor Quality**	1.66 (1.12, 2.46)	NA	NA	NA	NA	0.74 (0.24, 2.28)	NA	NA	NA
**WLHIV with antenatal ART vs HIV-negative women**	**Good Quality**	1.02 (0.54, 1.90)	0.38 (0.05, 2.83)	NA	0.93 (0.52, 1.68)	0.44 (0.06, 3.29)	1.28 (0.74, 2.20)	1.00 (0.30, 3.34)	NA	5.69 (0.81, 39.47)
	**Average Quality**	1.39 (1.13, 1.71)	1.68 (0.86, 3.25)	NA	2.19 (1.39, 3.45)	2.40 (1.37, 4.21)	1.98 (1.06, 3.71)	1.20 (1.09, 1.33)	NA	0.95 (0.57, 1.59)
	**Poor Quality**	1.31 (1.07, 1.61)	NA	NA	4.63 (2.21, 9.68)	NA	1.35 (0.98, 1.88)	1.58 (0.79, 3.17)	NA	NA
**ART-naïve WLHIV**										
**WLHIV with preconception ART vs ART-naïve WLHIV**	**Good Quality**	NA	NA	NA	NA	NA	NA	NA	NA	NA
	**Average Quality**	1.39 (0.50, 3.89)	NA	NA	1.32 (0.74, 2.35)	NA	1.41 (0.95, 2.08)	NA	NA	NA
	**Poor Quality**	1.10 (0.74, 1.64)	NA	NA	1.52 (1.18, 1.97)	NA	1.39 (1.07, 1.80)	NA	NA	NA

**WLHIV with antenatal ART vs ART-naïve WLHIV**	**Good Quality**	NA	NA	NA	NA	NA	NA	NA	NA	NA
	**Average Quality**	0.75 (0.46, 1.23)	NA	NA	1.24 (0.75, 2.07)	NA	1.39 (0.98, 1.99)	NA	NA	NA
	**Poor Quality**	0.97 (0.49, 1.92)	NA	NA	2.34 (0.48, 11.51)	NA	1.38 (1.06, 1.80)	NA	NA	NA

A RR > 1 indicates increased risk of a perinatal outcome associated with WLHIV with preconception/antenatal ART initiation. For example, WLHIV with preconception ART initiation are associated with an increased risk of preterm birth compared to HIV negative women (RR 1.51 (1.17, 1.95)) in average quality studies.

Abbreviations: ART= antiretroviral therapy, HIV= human immunodeficiency virus, LBW= low birthweight, NND= neonatal death, PTB= preterm birth, SGA= small for gestational age, sPTB= spontaneous preterm birth, VLBW= very low birthweight, VPTB= very preterm birth, VSGA= very small for gestational age.

Appendix 4.6

Subgroup analysis by study quality: Number of studies and women analysed

		Perinatal outcomes								
		**PTB**	**VPTB**	**sPTB**	**LBW**	**VLBW**	**SGA**	**VSGA**	**Stillbirth**	**NND**
		Number of studies (number of women analysed)	Number of studies (number of women analysed)	Number of studies (number of women analysed)	Number of studies (number of women analysed)	Number of studies (number of women analysed)	Number of studies (number of women analysed)	Number of studies (number of women analysed)	Number of studies (number of women analysed)	Number of studies (number of women analysed)
**HIV- Negative Women**										
**WLHIV with preconception ART vs HIV-negative women**	**Good Quality**	1 (442)	1 (442)	NA	1 (442)	1 (442)	1 (442)	1 (442)	NA	1 (442)
	**Average Quality**	9 (87,847)	4 (52,599)	NA	4 (16,842)	2 (9,844)	6 (83,407)	1 (39,133)	NA	1 (24,798)
	**Poor Quality**	3 (2,067)	NA	NA	NA	NA	1 (328)	NA	NA	NA
**WLHIV with antenatal ART vs HIV-negative women**	**Good Quality**	1 (475)	1 (475)	NA	1 (475)	1 (475)	1 (475)	1 (475)	NA	1 (475)
	**Average Quality**	12 (115,135)	4 (70,956)	NA	6 (27,490)	2 (10,458)	8 (103,441)	1 (56,435)	NA	1 (22,290)
	**Poor Quality**	4 (3,435)	NA	NA	1 (406)	NA	2 (1458)	1 (1079)	NA	NA
**ART-naïve WLHIV**										
**WLHIV with preconception ART vs ART-naïve WLHIV**	**Good Quality**	NA	NA	NA	NA	NA	NA	NA	NA	NA
	**Average Quality**	2 (1,300)	NA	NA	3 (1502)	NA	2 (805)	NA	NA	NA

	**Poor Quality**	5 (2499)	NA	NA	1 (1001)	NA	1 (1001)	NA	NA	NA
**WLHIV with antenatal ART vs ART-naïve WLHIV**	**Good Quality**	NA	NA	NA	NA	NA	NA	NA	NA	NA
	**Average Quality**	8 (8,523)	NA	NA	5 (5,089)	NA	5 (4,936)	NA	NA	NA
	**Poor Quality**	5 (3,780)	NA	NA	2 (1,103)	NA	1 (934)	NA	NA	NA

Abbreviations: ART= antiretroviral therapy, HIV= human immunodeficiency virus, LBW= low birthweight, NND= neonatal death, PTB= preterm birth, SGA= small for gestational age, sPTB= spontaneous preterm birth, VLBW= very low birthweight, VPTB= very preterm birth, VSGA= very small for gestational age.

Appendix 4.7

Subgroup analysis by study quality: I^2 values

		Perinatal outcomes								
		**PTB**	**VPTB**	**sPTB**	**LBW**	**VLBW**	**SGA**	**VSGA**	**Stillbirth**	**NND**
		I^2 (95%CI) (p value)	I^2 (95%CI) (p value)	I^2 (95%CI) (p value)	I^2 (95%CI) (p value)	I^2 (95%CI) (p value)	I^2 (95%CI) (p value)	I^2 (95%CI) (p value)	I^2 (95%CI) (p value)	I^2 (95%CI) (p value)
**HIV- Negative Women**										
**WLHIV with preconception ART vs HIV-negative women**	**Good Quality**	NA	NA	NA	NA	NA	NA	NA	NA	NA
	**Average Quality**	95.33% p<0.001	92.86% p<0.001	NA	93.55%	78.13%	99.55% P<0.001	NA	NA	NA
	**Poor Quality**	58.76 % p=0.10	NA	NA	p<0.001	NA	NA	NA	NA	NA
**WLHIV with antenatal ART vs HIV-negative women**	**Good Quality**	NA	NA	NA	NA	0.00% P=0.56	NA	NA	NA	NA
	**Average Quality**	93.20% P<0.001	86.08% P<0.001	NA	95.06% P<0.001	NA	98.97% P<0.001	NA	NA	NA
	**Poor Quality**	17.77% P=0.24	NA	NA	NA	NA	0.00% P=0.48	NA	NA	NA
**ART-naïve WLHIV**										
**WLHIV with preconception ART vs ART-naïve WLHIV**	**Good Quality**	NA	NA	NA	NA	NA	NA	NA	NA	NA
	**Average Quality**	71.20% P=0.06	NA	NA	59.72% P-0.07	NA	0.00% P=0.78	NA	NA	NA

	**Poor Quality**	70.77%<br>P=0.01	NA	NA	NA	NA	NA	NA	NA	NA
**WLHIV with antenatal ART vs ART-naïve WLHIV**	**Good Quality**	NA	NA	NA	NA	NA	NA	NA	NA	NA
	**Average Quality**	89.60%<br>P<0.001	NA	NA	85.26%<br>P<0.001	NA	46.41%<br>P=0.16	NA	NA	NA
	**Poor Quality**	92.80%<br>P<0.001	NA	NA	0.00%<br>P=0.21	NA	NA	NA	NA	NA

Abbreviations: ART= antiretroviral therapy, HIV= human immunodeficiency virus, LBW= low birthweight, NND= neonatal death, PTB= preterm birth, SGA= small for gestational age, sPTB= spontaneous preterm birth, VLBW= very low birthweight, VPTB= very preterm birth, VSGA= very small for gestational age.

Appendix 4.8

Subgroup analysis by study quality: test of subgroup difference (interaction)

Subgroup analyses by income status	Perinatal outcomes								
	**PTB**	**VPTB**	**sPTB**	**LBW**	**VLBW**	**SGA**	**VSGA**	**Stillbirth**	**NND**
	p-value	p-value	p-value	p-value	p-value	p-value	p-value	p-value	p-value
**WLHIV preconception ART vs HIV- women**									
**Good vs Average quality vs poor quality**	P=0.88	p=0.58	NA	p=0.35	P=0.37	p=0.28	P=0.01	NA	P=0.26
**WLHIV antenatal ART vs HIV- women**									
**Good vs Average quality vs poor quality**	P=0.64	P=0.17	NA	P<0.001	P=0.11	P=0.52	P=0.71	NA	P=0.08
**WLHIV Preconception ART vs WLHIV naïve to ART**									
**Good vs Average quality vs poor quality**	P=0.68	NA	NA	P=0.66	NA	P=0.96	NA	NA	NA
**WLHIV Antenatal ART vs WLHIV naïve to ART**									
**Good vs Average quality vs poor quality**	P=0.55	NA	NA	P=0.32	NA	P=0.97	NA	NA	NA

Test of subgroup differences by study quality using a random-effects DerSimonian-Laird model. Abbreviations: ART= antiretroviral therapy, LBW= low birthweight, NND= neonatal death, PTB= preterm birth, SGA= small for gestational age, sPTB= spontaneous preterm birth, VLBW= very low birthweight, VPTB= very preterm birth, VSGA= very small for gestational age.

Appendix 5: Sensitivity analyses

Adjusting for confounders in individual studies comparing women living with HIV receiving preconception and antenatal ART with HIV negative women and WLHIV naïve to ART for preterm birth, very preterm birth, low birthweight, small for gestational age, very small for gestational age, stillbirth and neonatal death.

Perinatal outcome															
**Study**		**PTB**		**VPTB**		**LBW**		**SGA**		**VSGA**		**Stillbirth**		**NND**	
		OR/RR (95% CI)	aOR/aRR (95% CI)	OR/RR (95% CI)	aOR/aRR (95% CI)	OR/RR (95% CI)	aOR/aRR (95% CI)	OR/RR (95% CI)	aOR/aRR (95% CI)	OR/RR (95% CI)	aOR/aRR (95% CI)	OR/RR (95% CI)	aOR/aRR (95% CI)	OR/RR (95% CI)	aOR/aRR (95% CI)
**Chen (2012)**[28]	No ART	ref	ref					ref	ref						
	HAART Antenatal Initation	1.5 (1.2–1.8)	1.4 (1.2–1.8)					1.7 (1.4–2.0)	1.5 (1.2–1.9)						
**Ekouevi (2008)**[31]	No ART HIV					ref	ref								
	Antenatal initiation					2.42 (1.07-5.43)	2.12 (1.15-4.65)								
	Preconception					3.17 (1.07-9.46)	2.88 (1.10-7.51)								
**Malaba (2021)**[38]	HIV Negative	ref	ref					ref	ref						
	Preconception on ART	1.02 (0.84-1.24)	0.97 (0.79-1.18)					1.49 (1.13-1.97)	1.39 (1.05-1.84)						
	Antenatal ART	1.11 (0.92-1.34)	1.07 (0.89-1.31)					1.34 (1.01-1.78)	1.36 (1.03-1.78)						
**Moodley (2016)**[40]	WLHIV Naïve to ART	ref	ref			ref	ref	ref	ref						
	Antenatal NVP-based ART	0.68 (0.46-0.98)	0.21 (0.08-0.55)			0.61 (0.4-0.93)	0.52 (0.35-0.78)	0.89 (0.50-1.59)	0.77 (0.44-1.35)						

	initiation														
	Antenatal EFV-based ART initiation	0.56 (0.39-0.8)	0.31 (0.11-0.9)			0.09 (0.03-0.24)	0.12 (0.04-0.37)	0.29 (0.08-1.07)	0.25 (0.07-0.87)						
**Zash (2018)** [55]	HIV-	ref	ref	ref	ref			ref	ref	ref	ref	ref	ref	ref	ref
	HIV+ DTG/EFV-based ART antenatal initiation	1·18 (1·12, 1·25)	1.18 (1.12-1.25)	1.01 (0·88,1.16)	1.01 (0.88-1.16)			1.23 (1.17,1.31)	1.30 (1.23-1.38)	1.21 (1.09,1.34)	1.28 (1.16-1.42)	1.10 (0.92,1.30)	1.08 (0.91, 1.29)	0.94 (0.75,1.18)	0.92 (0.73-1.17)

Abbreviations: ART= antiretroviral therapy, DTG = dolutegravir, EFV = efavirenz, HIV= human immunodeficiency virus, LBW= low birthweight, NND= neonatal death, NVP = nevirapine, PTB= preterm birth, SGA= small for gestational age, VPTB= very preterm birth, VSGA= very small for gestational age.

Appendix 6: Funnel plots

<u>Appendix 6.1: Women living with HIV receiving preconception ART vs HIV-negative women</u>

Figure 6.1.1 Preterm birth in women living with HIV receiving preconception ART vs HIV-women

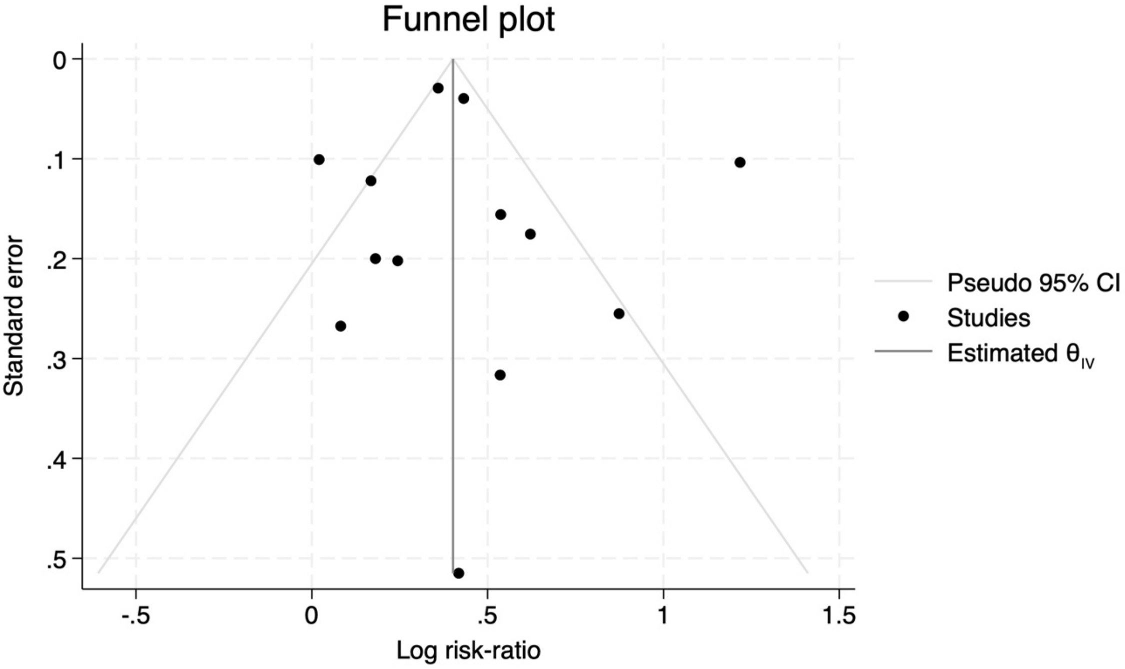

Figure 6.1.2 Very preterm birth in women living with HIV receiving preconception ART vs HIV- negative women

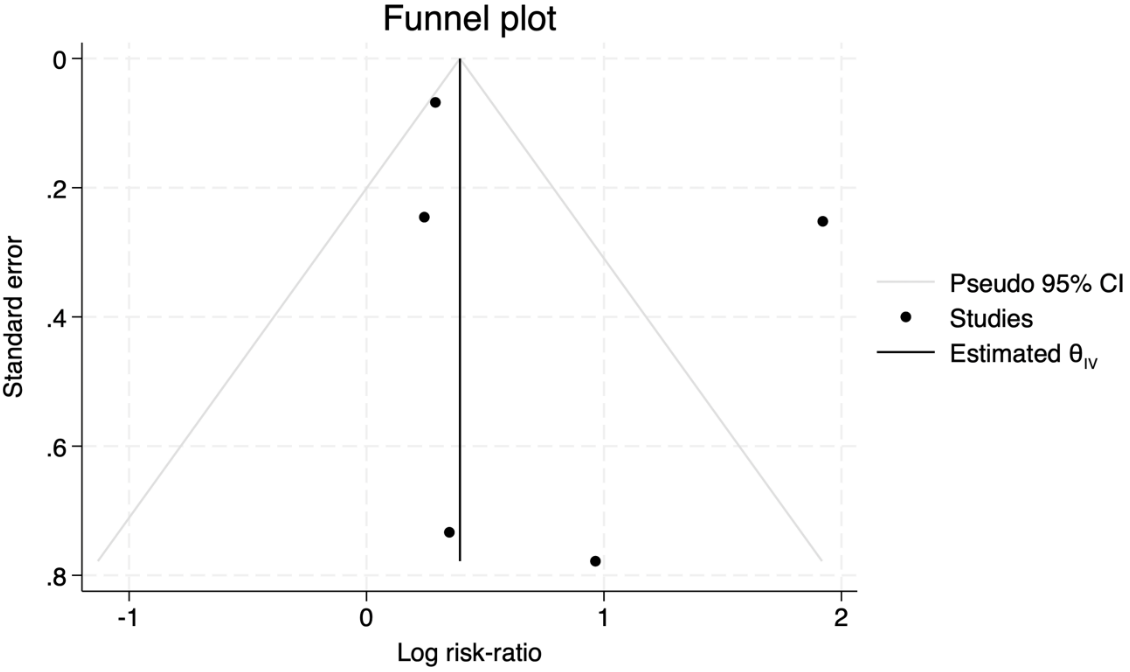

Figure 6.1.3 Low birthweight in women living with HIV receiving preconception ART vs HIV-negative women

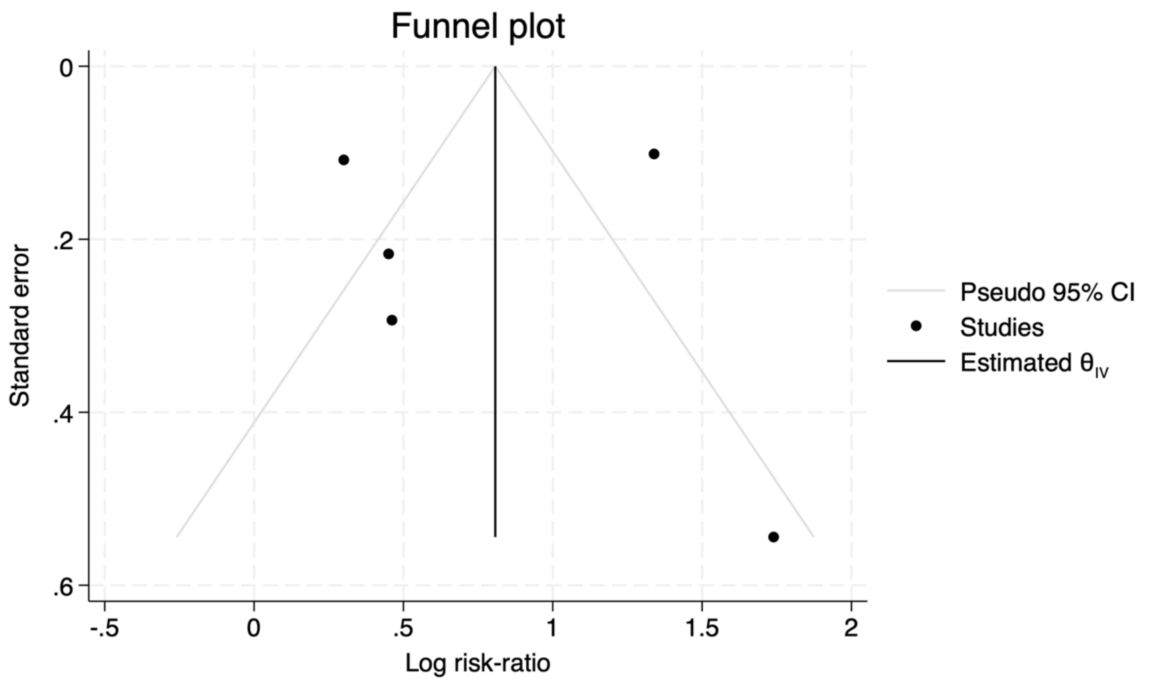

Figure 6.1.4 Very low birthweight in women living with HIV receiving preconception ART vs HIV- negative women

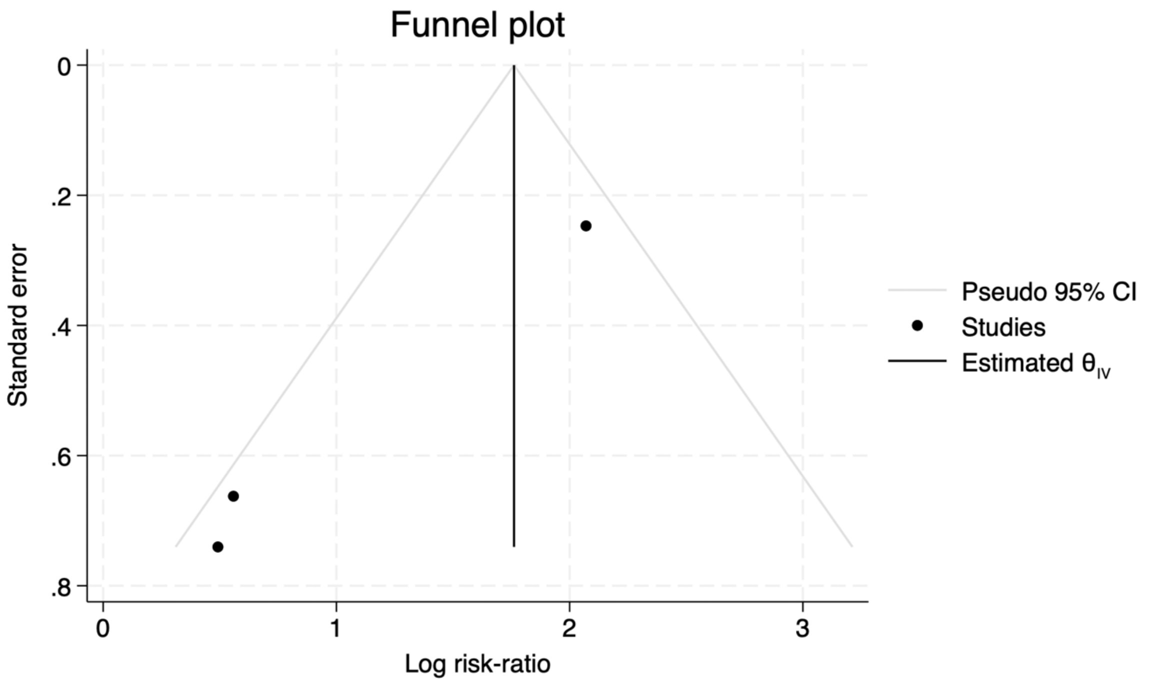

Figure 6.1.5 Small for gestational age in women living with HIV receiving preconception ART vs HIV- negative women

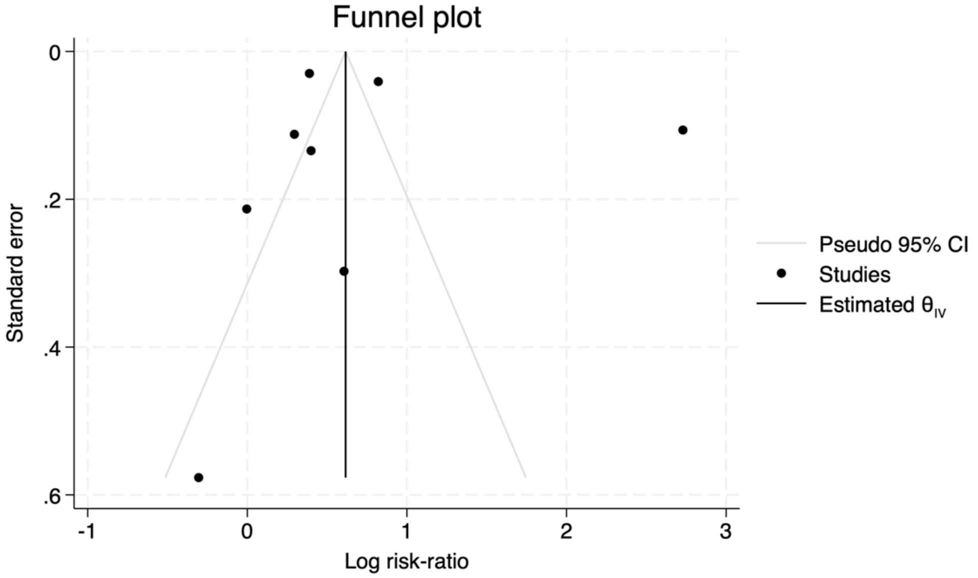

Figure 6.1.6 Very small for gestational age in women living with HIV receiving preconception ART vs HIV- negative women

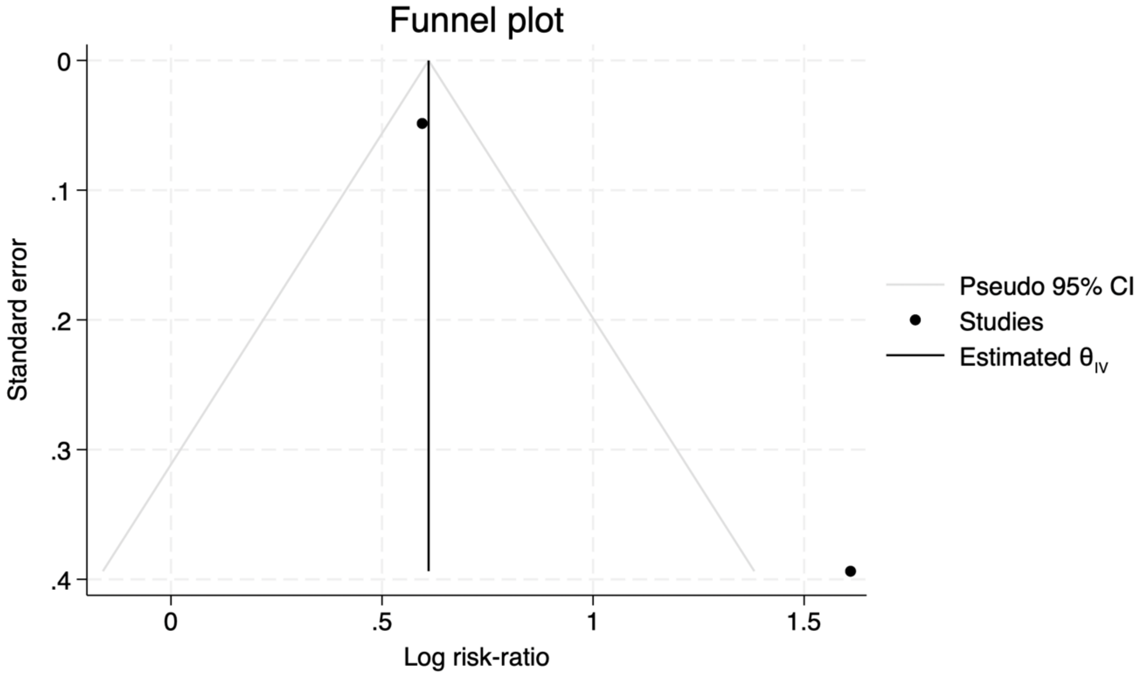

Figure 6.1.7 Neonatal death in women living with HIV receiving preconception ART vs HIV-negative women

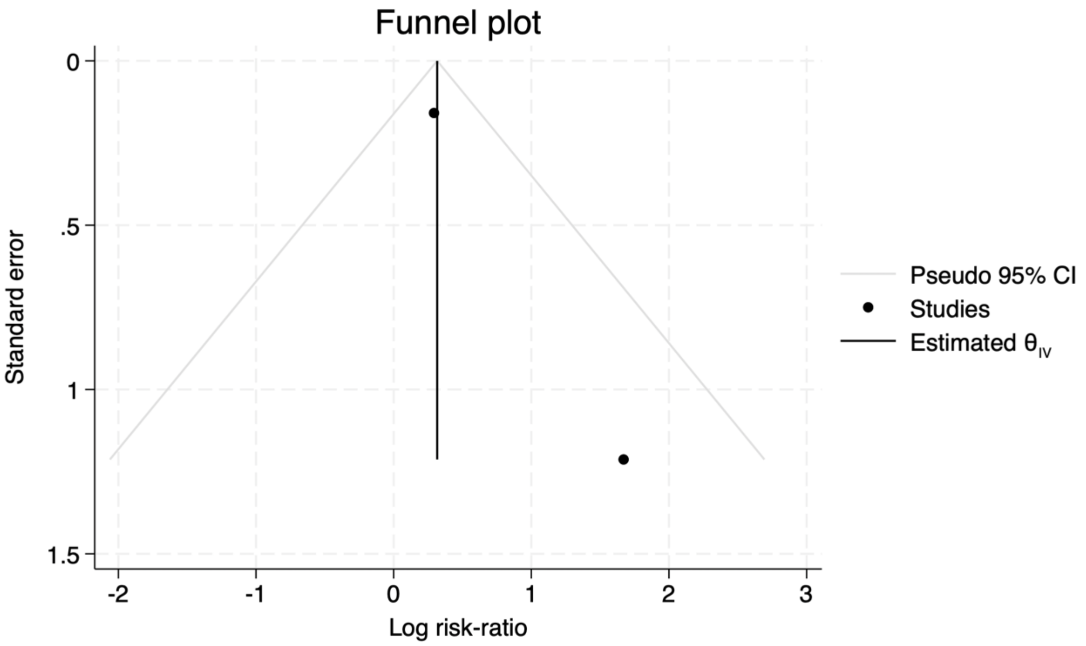

Appendix 6.2: Women living with HIV receiving antenatal ART vs HIV-negative women

Appendix 6.2.1: Preterm birth in women living with HIV receiving antenatal ART vs HIV-women

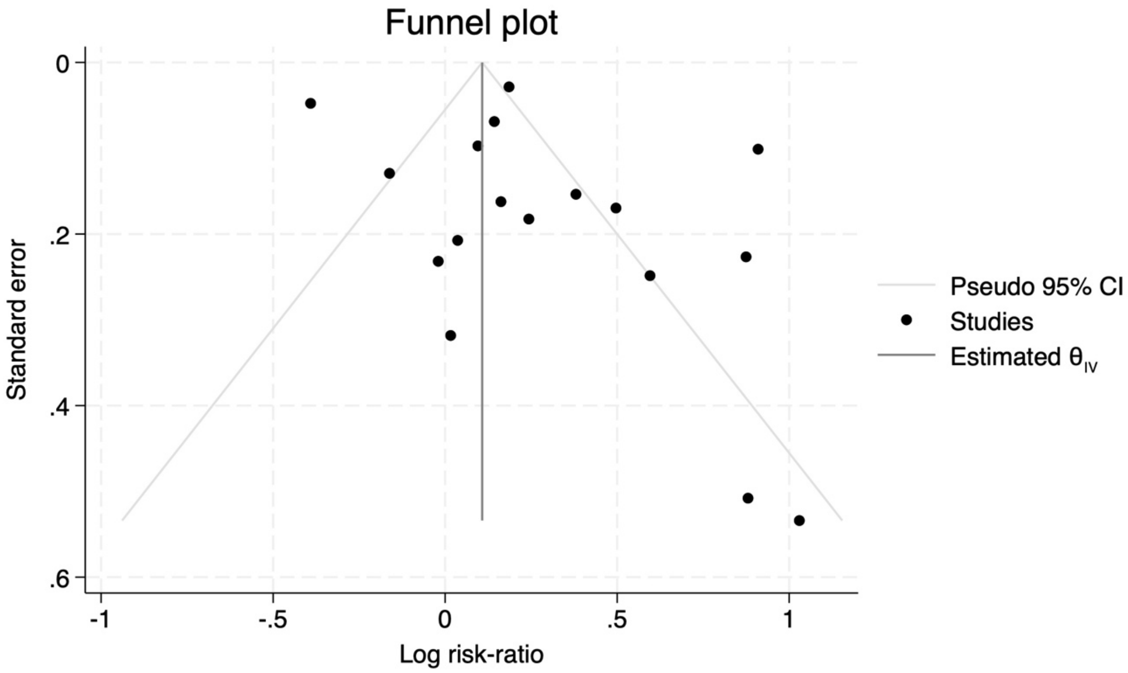

Figure 6.2.2: Very preterm birth in women living with HIV receiving antenatal ART vs HIV-negative women

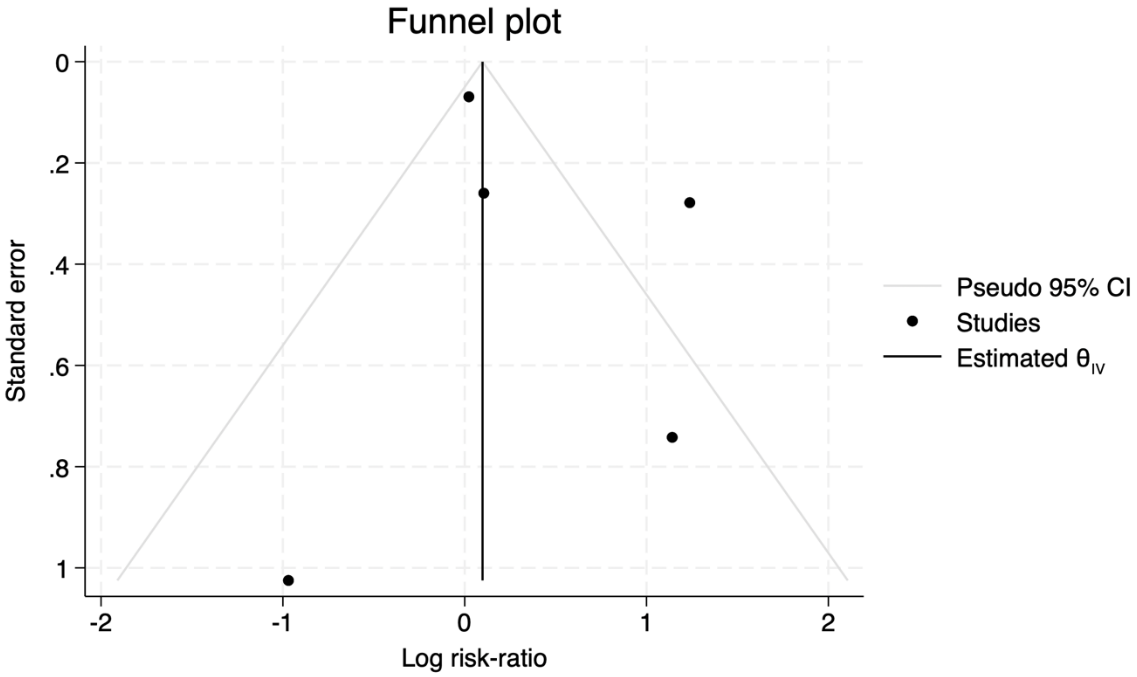

Figure 6.2.3: Low birthweight in women living with HIV receiving antenatal ART vs HIV-negative women

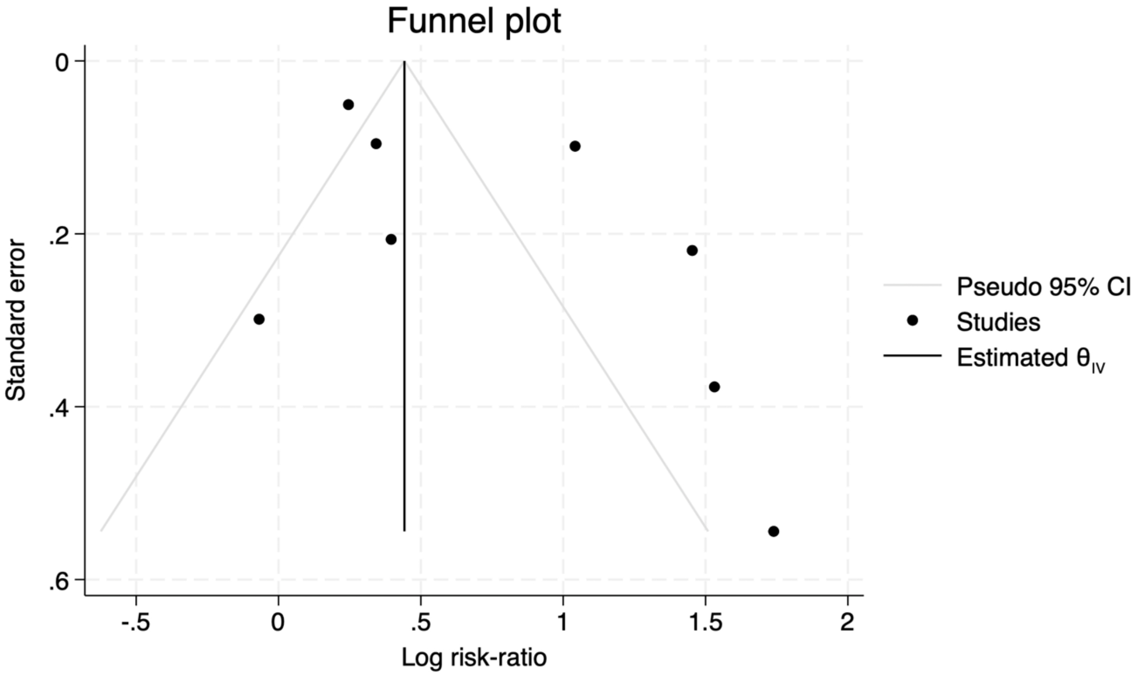

Figure 6.2.4: Very low birthweight in women living with HIV receiving antenatal ART vs HIV-negative women

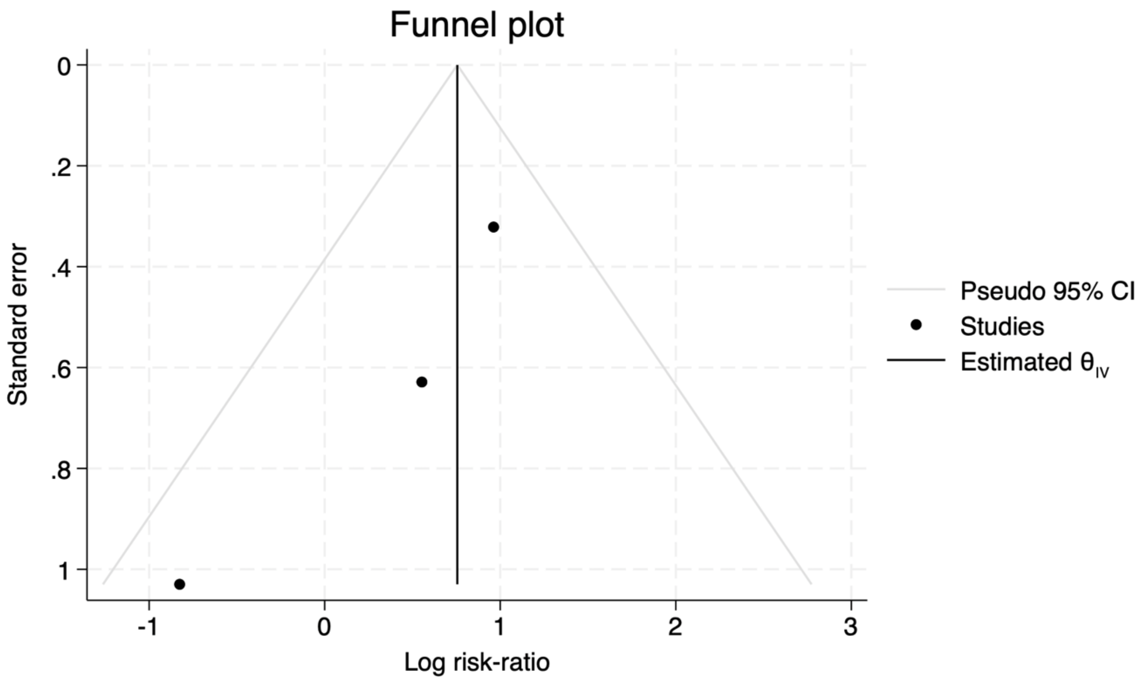

Figure 6.1.5: Small for gestational age in women living with HIV receiving antenatal ART vs HIV- negative women

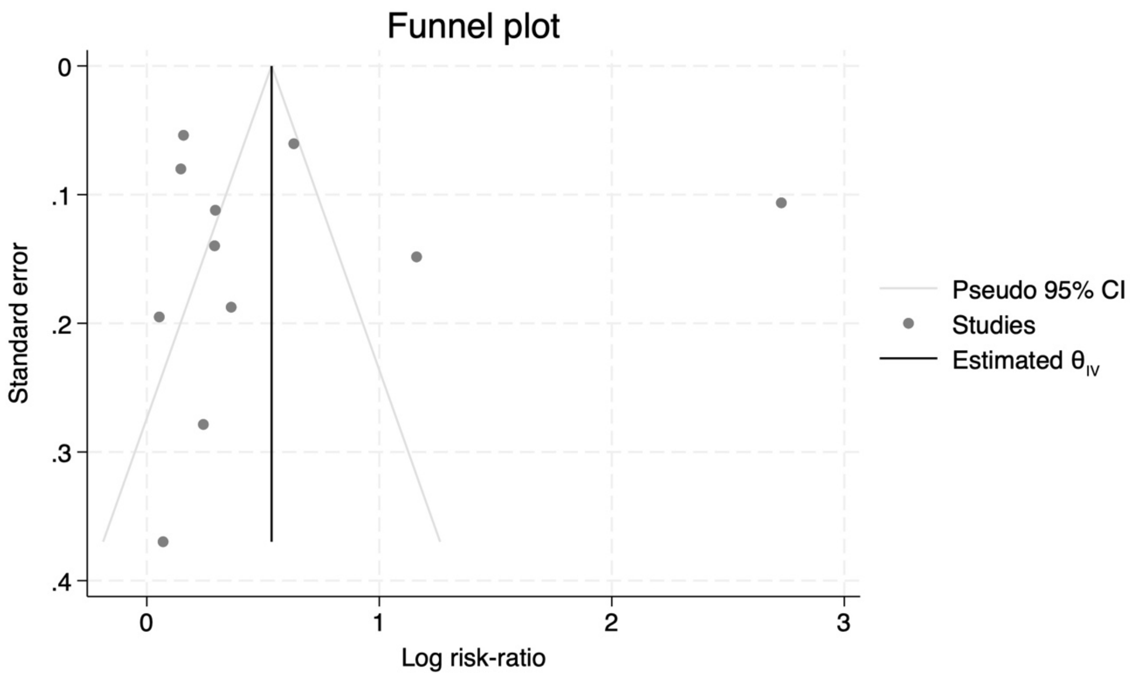

Figure 6.2.6: Very small for gestational age in women living with HIV receiving antenatal ART vs HIV- negative women

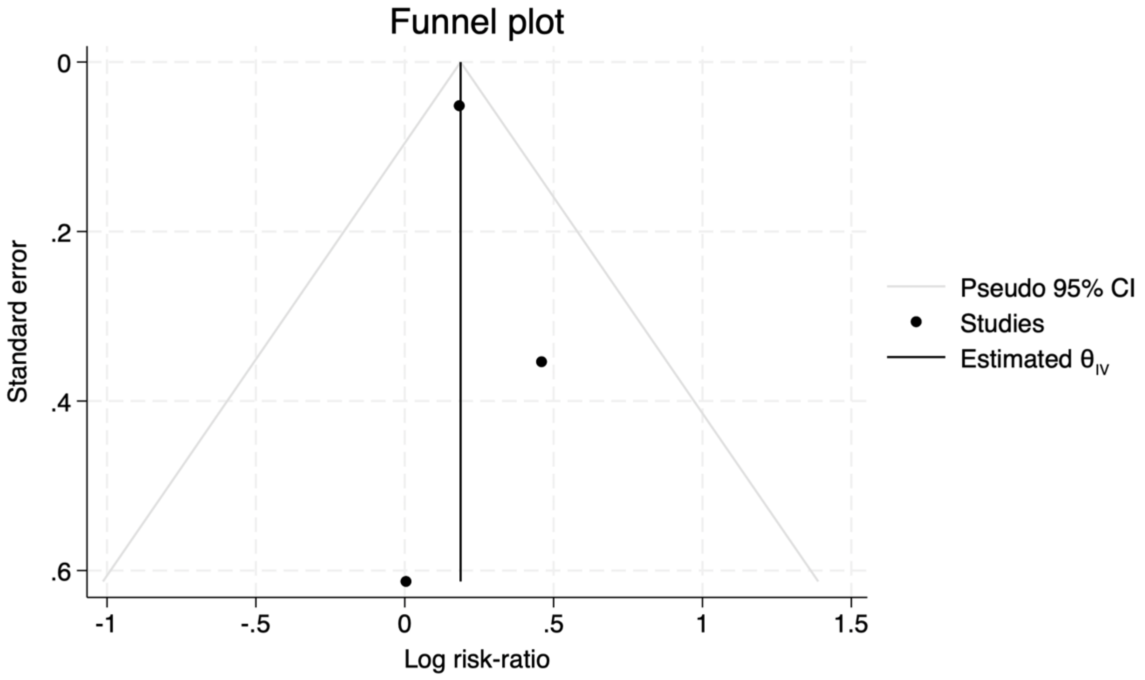

Figure 6.2.7: Neonatal death in women living with HIV receiving antenatal ART vs HIV- negative women

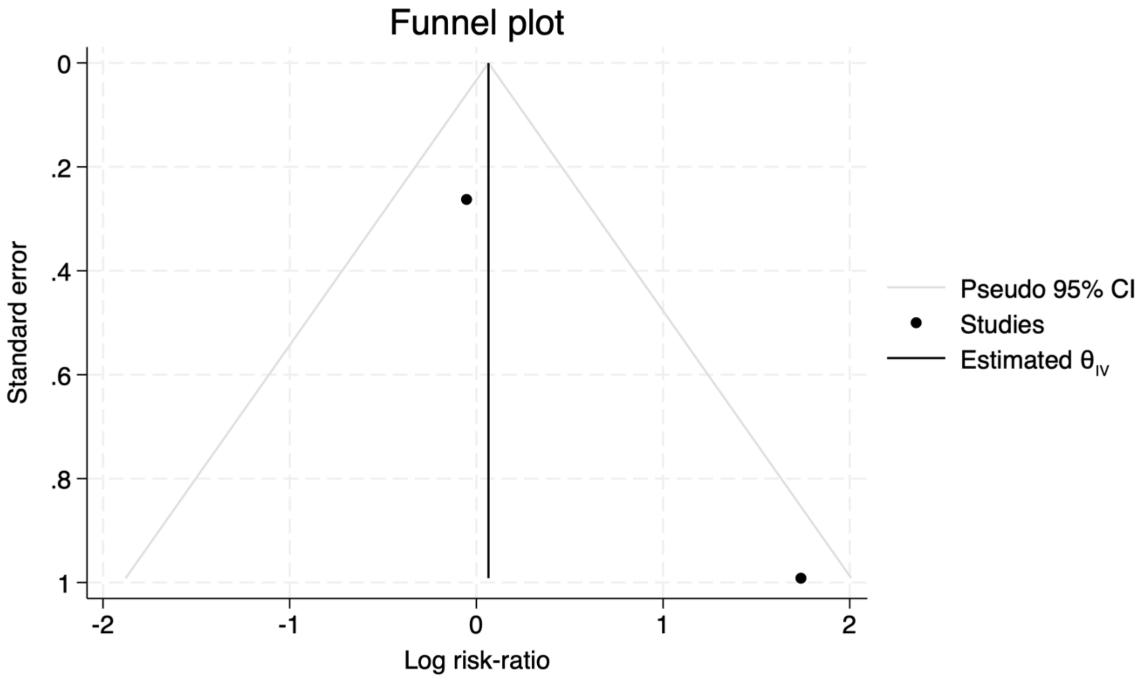

Appendix 6.3: Women living with HIV receiving preconception ART vs women living with HIV naïve to ART

Figure 6.3.1: Preterm birth in women living with HIV receiving preconception ART vs women living with HIV naïve to ART

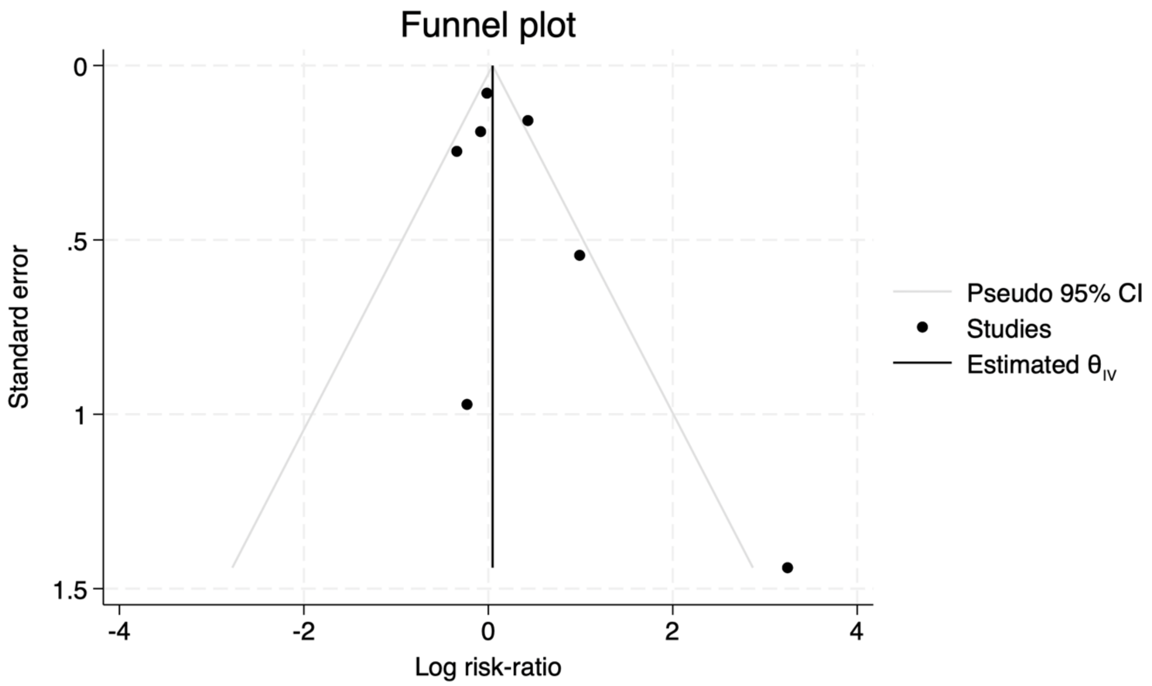

Figure 6.3.2: Low birth weight in women living with HIV receiving preconception ART vs women living with HIV naïve to ART

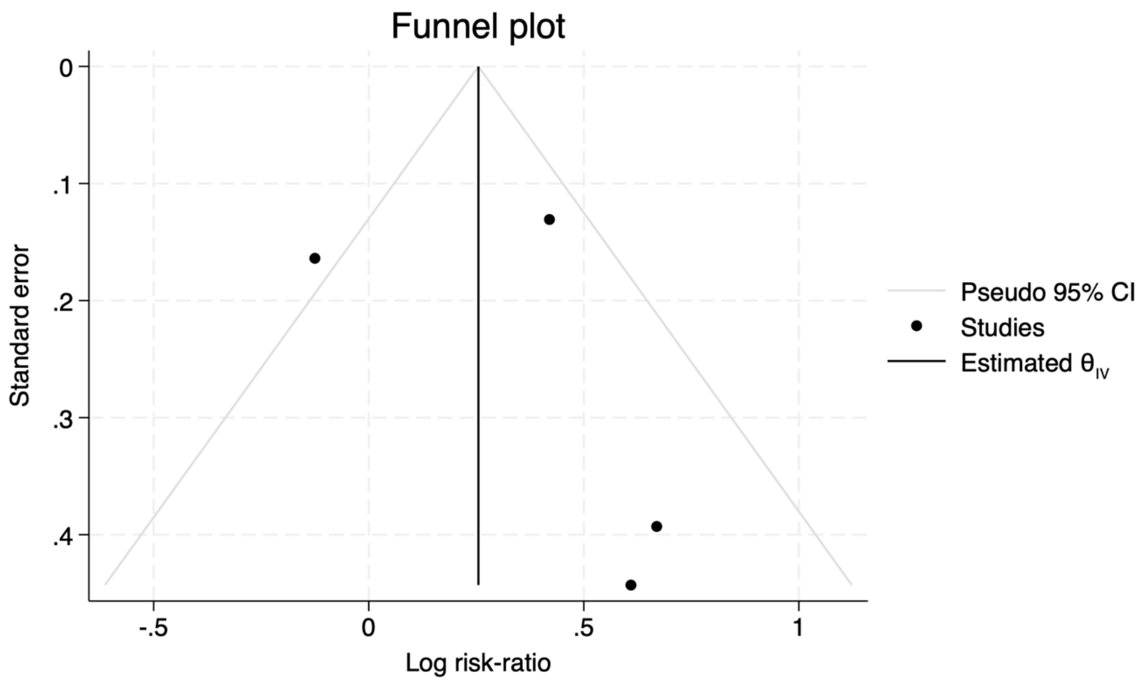

Figure 6.3.3: Small for gestational age in women living with HIV receiving preconception ART vs women living with HIV naïve to ART

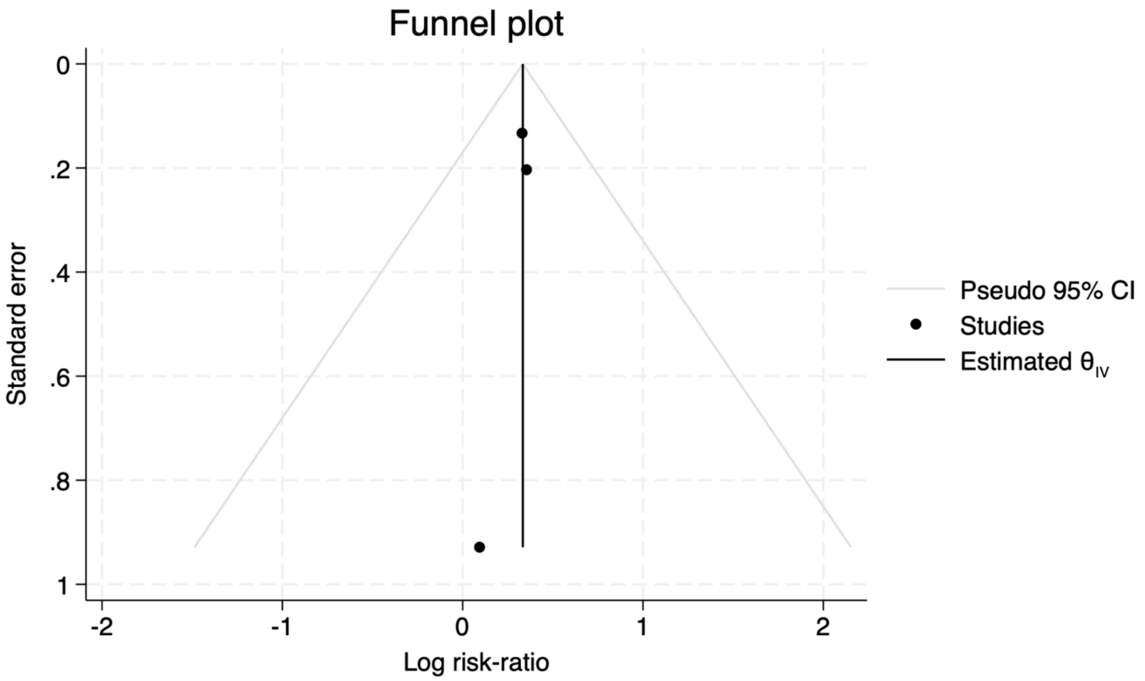

Appendix 6.4: Women living with HIV receiving antenatal ART vs women living with HIV naïve to ART

Figure 6.4.1: Preterm birth in women living with HIV receiving antenatal ART vs women living with HIV naïve to ART

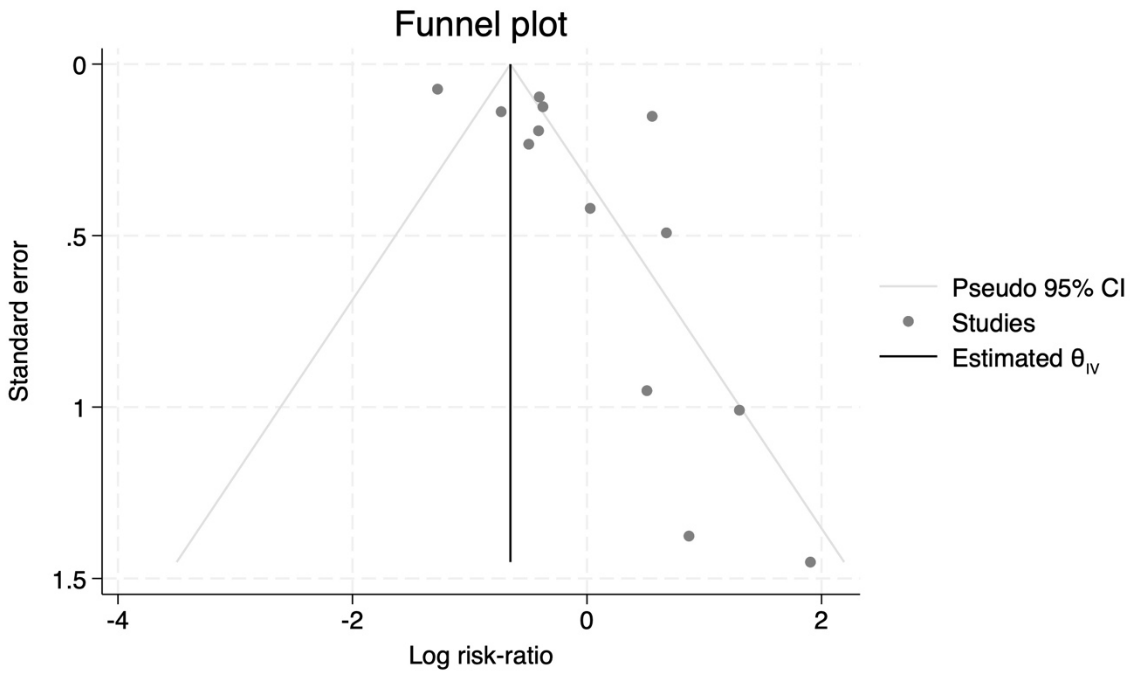

Figure 6.4.2: Low birth weight in women living with HIV receiving antenatal ART vs women living with HIV naïve to ART

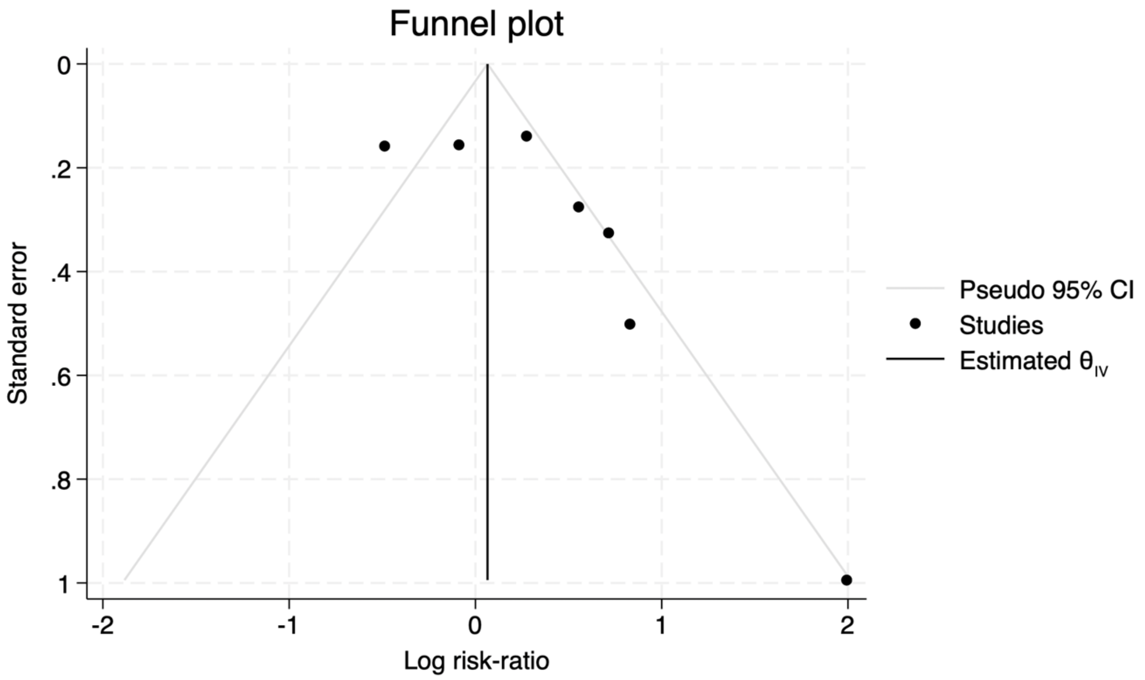

Figure 6.4.3: Small for gestational age in women living with HIV receiving antenatal ART vs women living with HIV naïve to ART

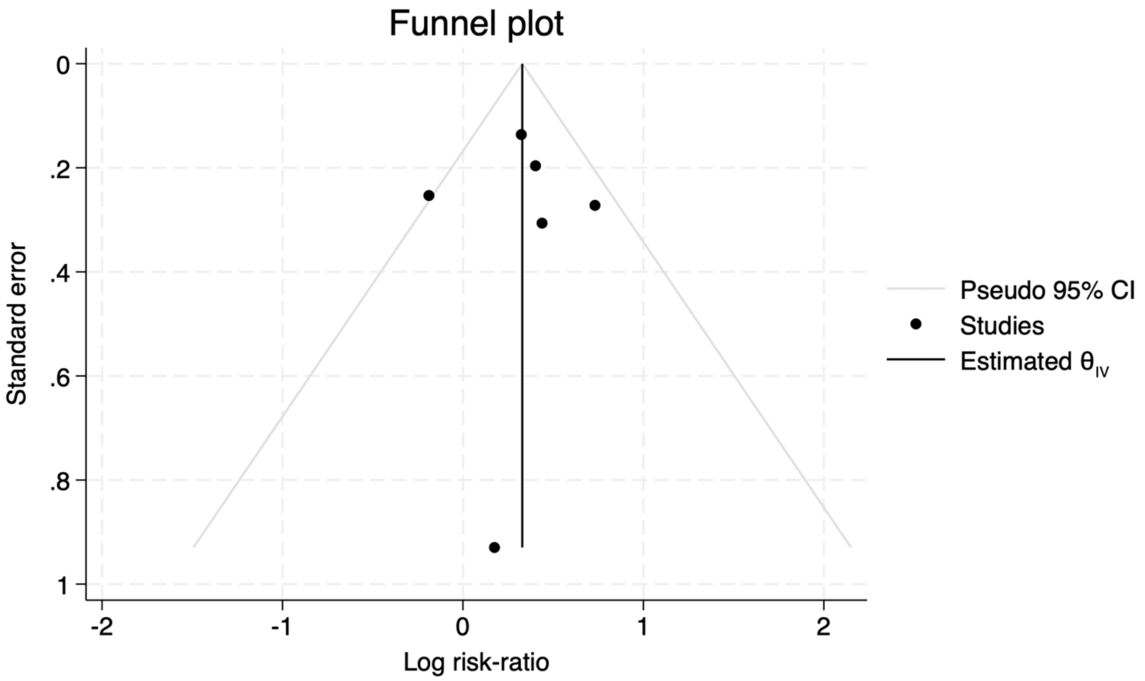

Supplement: Supplemental Digital Content [file aids-39-584-s001.pdf]
